# Cancer Immunotherapy via Disruption of Integrin αvβ3 and CD47 Costabilization on Cancer Cell Surface

**DOI:** 10.1002/advs.202501602

**Published:** 2025-10-30

**Authors:** Peng‐Cheng Yu, Chen‐Xi Yue, Wen‐Zhong Dong, Cui‐Yun Hao, Yi‐Fan Qiao, Di Liu, Xin Zhang, Qi Zhan, Jia‐Bao Yao, Dong‐Ping Wang, Peng Cao, Ying‐Zhe Fan, Ye Yu

**Affiliations:** ^1^ Schools of Basic Medicine and Clinical pharmacy and State Key Laboratory of Natural Medicines China Pharmaceutical University Nanjing 210009 China; ^2^ Interventional Cancer Institute of Chinese Integrative Medicine Putuo Hospital Shanghai University of Traditional Chinese Medicine Shanghai 200062 China; ^3^ Key Laboratory of Preclinical Study for New Drugs of Gansu Province School of Basic Medical Sciences Lanzhou University Lanzhou 730000 China; ^4^ Hospital of Integrated Traditional Chinese and Western Medicine Nanjing University of Chinese Medicine Nanjing 210023 China

**Keywords:** cancer immunotherapy, CD47, immune checkpoint, integrin αvβ3, peptides

## Abstract

CD47/signal‐regulatory protein α (SIRPα) signaling enables malignant cells to evade macrophage‐mediated phagocytosis, offering a promising strategy for cancer therapy via immune checkpoint blockade. However, this strategy is widely debated due to several safety risks revealed by clinical studies, including anemia. Here, a CD47–SIRPα immune checkpoint treatment is investigated that mitigates anemic side effects by selectively interfering with the costabilization of CD47 and integrin αvβ3 on cancer cell surfaces, a phenomenon absent in erythrocytes. Multiplexed immunofluorescence analysis of 119 clinical breast cancer tissues reveals this costabilization. The engineered peptide PSFL‐NK13 effectively disrupts this costabilization, which enhances macrophage phagocytosis and delays tumor growth, without causing anemia or promoting angiogenesis. Thus, a stable interaction is identified between integrin αvβ3 and CD47 on the cancer cell membrane that facilitates immune evasion and demonstrates that targeting this interaction offers a safer therapeutic strategy for various tumors.

## Graphical abstract

This study identifies a cancer‐specific interaction between CD47 and integrin αvβ3 that facilitates immune evasion. A rationally designed peptide, PSFL‐NK13, disrupts this axis, enhancing macrophage‐mediated phagocytosis and suppressing tumor growth without inducing anemia. These findings establish a safer immune checkpoint strategy distinct from conventional CD47‐targeting therapies.

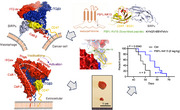

## 1 Introduction

CD47 is highly expressed in a range of malignant cells, such as myeloma, smooth muscle sarcoma, acute lymphoblastic leukemia, non‐Hodgkin's lymphoma, breast cancer, head and neck cancer, and osteosarcoma.^[^ ^1^, ^2^ ^]^ Elevated CD47 expression has been identified as an independent prognostic factor associated with unfavorable clinical outcomes.^[^ ^3^, ^4^, ^5^ ^]^ Macrophages recognize “self” and “nonself” using CD47 expressed on the surface of cancer cells, and CD47 can interact with signal‐regulatory protein α (SIRPα) on macrophages to signal “do not eat me,” thus preventing macrophage‐mediated phagocytosis of tumor.^[^ ^6^, ^7^ ^]^ Phagocytosis of tumor cells by macrophages can be restored by using antibodies to inhibit the CD47–SIRPα signaling pathway (e.g., Hu5F9‐G4 in combination with rituximab in patients with invasive lymphoma).^[^ ^4^, ^8^, ^9^ ^]^ Currently, more than 300 investigational studies are exploring the CD47–SIRPα checkpoint, with 45 entering clinical trial phases.^[^ ^10^ ^]^ However, CD47 also dominates erythrocyte clearance and homeostasis in vivo.^[^ ^11^ ^]^ On the surface of senescent erythrocytes, CD47 expression is reduced, and “eat me” signaling eventually overwhelms the “do not eat me” signaling of CD47, promoting macrophage phagocytosis of senescent erythrocytes.^[^ ^12^ ^]^ Consequently, CD47 antibodies that “kill” tumors may simultaneously cause erythrocyte lysis, leading to dose‐limiting toxicity, such as anemia and thrombocytopenia.^[^ ^1^, ^13^ ^]^ Complicating the therapeutic challenge, the antibodies bind indiscriminately to CD47 expressed on erythrocytes, creating a physiological sink that sequesters the agents and reduces their availability to target tumor cells.

Integrins, ubiquitous heterodimeric transmembrane glycoprotein adhesion receptors, play pivotal roles throughout all stages of cancer, from primary tumor initiation to late‐stage metastasis.^[^ ^14^ ^]^ These receptors serve as mechanotransducers, signaling hubs, and central components of the migratory apparatus, thereby contributing to tumorigenesis through a multiplicity of roles.^[^ ^14^ ^]^ Furthermore, integrins finely discriminate between different microenvironments, thereby determining cellular responses to various signals.^[^ ^15^, ^16^ ^]^ To achieve such exquisite regulations, each integrin consists of an α‐subunit and a β‐subunit, of which 18 α‐subunits and 8 β‐subunits are assembled in a stable, noncovalent manner to produce 24 known functionally distinct heterodimers, respectively. Among these heterodimers, integrins can be divided into two major groups based on their capability of recognizing the Arg–Gly–Asp (RGD) peptide motif.^[^ ^17^, ^18^ ^]^ These heterodimers are subdivided based on their recognition of the RGD tripeptide motif. Eight RGD‐binding integrins—αvβ1, αvβ3, αvβ5, αvβ6, αvβ8, α5β1, α8β1, and αIIbβ3—form a specialized subgroup with well‐documented roles in oncogenesis and metastatic spread.^[^ ^19^, ^20^, ^21^ ^]^ Inhibition of these integrins has yielded promising results in curbing tumor growth, angiogenesis, and metastasis in preclinical models. Regulatory mechanisms, such as conformational changes (inactivation, activation, and a range of intermediate states), interactions with other proteins, and endocytosis/recycling, enable integrins to participate in a wide range of pathophysiological processes.^[^ ^15^, ^22^ ^]^ Notably, integrin αvβ3 is the most well‐studied integrin implicated in tumor development, particularly in tumor angiogenesis.^[^ ^23^, ^24^ ^]^ Although small molecules and peptides targeting integrins are currently used to treat fibrosis and ocular vascular disease,^[^ ^25^, ^26^ ^]^ questions remain to be addressed in terms of safety and efficacy of antitumor agents targeting αv family integrins (αvβ3 and αvβ5).^[^ ^27^, ^28^ ^]^ Notably, low‐dose αvβ3 antagonists may enhance, rather than inhibit, tumor angiogenesis.^[^ ^25^, ^29^ ^]^

Although both αvβ3 and CD47 are abundantly expressed in a variety of malignancies, it is suggested that targeting either CD47 or αvβ3 alone is not very sufficient for cancer treatment.^[^ ^30^, ^31^ ^]^ Human erythrocytes express CD47 but not αvβ3, while in vitro protein immunoprecipitation also revealed direct interaction between αvβ3 and CD47.^[^ ^32^, ^33^, ^34^ ^]^ These findings raise several questions:^[^ ^35^, ^36^ ^]^ are αvβ3 and CD47 on tumor cells functionally connected through direct interactions? Are these interactions static or dynamic? Is αvβ3 in an activated or inactivated state when interacting with CD47 on tumor cells? These observations also lead to a bolder hypothesis. If αvβ3, coexisting with CD47 on the surface of cancer cells, regulates the CD47–SIRPα immune checkpoint, then an immune checkpoint therapy that specifically interferes with CD47/αvβ3 interactions on cancer cells should avoid side effects on red blood cells, which do not express αvβ3, unlike CD47 antibodies that target CD47 alone.^[^ ^37^, ^38^ ^]^ Furthermore, specifically targeting the interfaces that determine CD47/αvβ3 interactions on cancer cells is expected to have fewer side effects compared to targeting CD47 or αvβ3 alone. This is because such targeting can avoid impairing the normal physiological functions of CD47 by affecting its binding to other ligands such as thromboxane‐1 (TSP‐1), vascular endothelial growth factor receptor 2 (VEGFR2), CD36, and Fas (CD95).^[^ ^2^, ^39^ ^]^ Additionally, this approach may avoid the side effects associated with directly inhibiting αvβ3's recognition of extracellular matrix (ECM) proteins containing RGD motifs.^[^ ^19^ ^]^

In this study, we found a pronounced correlation between CD47 and αvβ3 expression in clinical samples of 119 breast cancer tissues and nine cancer cell lines, particularly the triple‐negative breast cancer (TNBC) cell lines MDA‐MB‐231.^[^ ^40^ ^]^ Reducing the surface expression of either CD47 or αvβ3 on tumor cells substantially lowered the distribution of the other on the membrane. This reduction weakened the CD47–SIRPα axis signaling, enhanced macrophage‐mediated phagocytosis of cancer cells, and significantly suppressed tumor growth. The tridecapeptide PSFL‐NK13, specifically designed to target this complex, significantly reduced the surface expression of both CD47 and αvβ3 in tumor cells. This action enhanced phagocytosis by macrophages, delayed the growth of in situ transplanted tumors in mice, and proved effective against various malignancies. Crucially, PSFL‐NK13 does not induce tumor angiogenesis at low doses, a phenomenon observed with certain αvβ3 inhibitors. Moreover, unlike CD47 antibodies associated with erythrocytolysis, PSFL‐NK13 does not pose the risk of serious side effects. Hence, PSFL‐NK13 could be a potentially safer candidate for the treatment of specific cancers.

## 2 Results

### 2.1 Correlation between the Elevated Surface Expression of αvβ3 and CD47 Observed in 119 Clinical Breast Cancer Tissues and across Distinct Cancer Cell Lines

**Figure 1:**
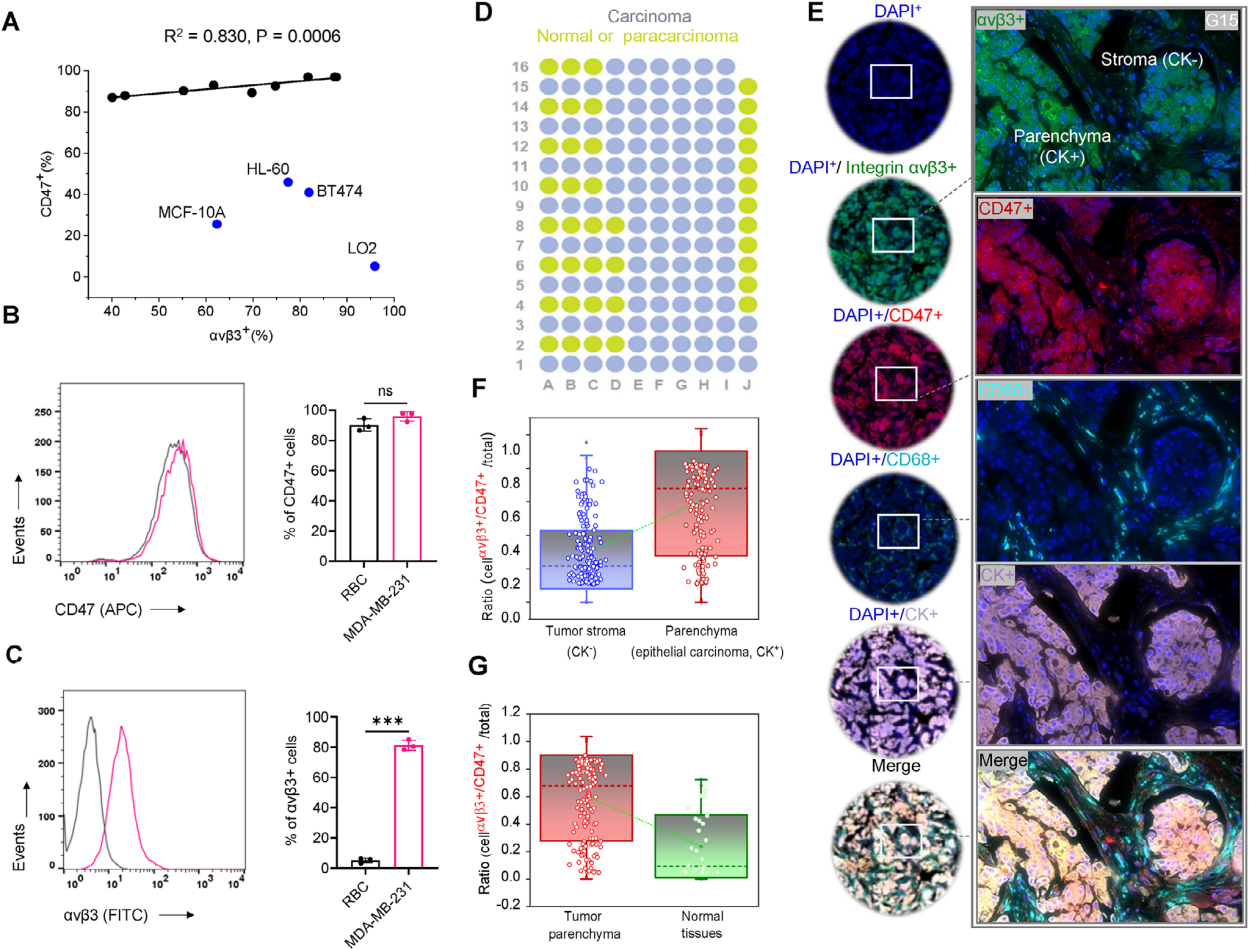
Coexpression of CD47 and integrin αvβ3 on tumor cells and in clinical breast cancer samples. A) A correlation analysis across selected tumor lines—MCF‐7, LoVo, HCT116, A549, MDA‐MB‐231, U87, H23, MDA‐MB‐468, and Hs578T—demonstrated a robust positive relationship between αvβ3 and CD47 expression (*R* ^2^ = 0.830, *p* < 0.0001). Notably, this correlation did not extend to nontumorigenic cell lines MCF‐10A and LO2, the hematopoietic tumor cell line HL60, or the he triple‐positive (ER^+^/PR^+^/HER2^+^) BT474 breast cancer line, which collectively exhibited divergent expression profiles. B,C) MDA‐MB‐231 cells and red blood cells (RBCs) were labeled and analyzed for CD47 and αvβ3 expression by flow cytometry. Pooled data for CD47 and αvβ3 cell surface markers are presented. D) Schematic representation of tissue microarray (TMA) for clinical breast cancer tissue samples along with paired paraneoplastic and normal tissues. E) Representative images of multiplex fluorescent immunohistochemical staining showing DAPI^+^ (blue), αvβ3^+^ (green), CD47^+^ (red), CD68^+^ (sky blue), and CK^+^ (grayish purple) in breast cancer tissue. Enlarged areas within dashed boxes are shown in the right panel. A merged image of all channels is displayed at the bottom. Scale bar = 50 µm. F,G) Statistical analysis of the proportion of αvβ3 and CD47 double‐positive cells in breast cancer and normal/paracarcinoma tissues. All data are expressed as mean ± s.e.m.; ^***^ *p* < 0.001 versus control, unpaired *t*‐test (C, D, G, and H); ns, not significant.

To rigorously evaluate the hypothesized coexpression of integrin αvβ3 and CD47 on the surface of cancer cells, we conducted an extensive flow cytometric analysis spanning a diverse array of human cell lines. These included solid tumor‐derived lines such as NCI‐H23, HCT116, LoVo, A549, U87, MCF‐7, BT474, Hs578T, MDA‐MB‐468, and MDA‐MB‐231, the hematologic cancer line HL‐60, and nontumorigenic epithelial controls MCF‐10A and LO2. Our results demonstrated a pronounced correlation between αvβ3 and CD47 surface expression in the majority of tumor‐derived lines (*R* ^2^ = 0.830, *p* < 0.001; **Figure** **1**A), including those of colorectal, lung, glioblastoma, and breast origin. However, this association was not observed in the nonmalignant MCF‐10A and LO2 lines, nor in HL‐60 or the triple‐positive breast cancer BT474 cell line (estrogen receptor (ER^+^)/progesterone receptor (PR^+^)/proto‐oncogene HER2 (HER2^+^)) (Figure S1, Supporting Information). These findings underscore that CD47 and αvβ3 coexpression is not a pan‐cancer phenomenon, but rather selectively enriched in particular malignancies—most notably in triple‐negative breast cancers (TNBC, ER^−^/PR^−^/HER2^−^) such as MDA‐MB‐231, Hs578T, and MDA‐MB‐468.^[^ ^41^, ^42^ ^]^

To deepen our understanding of the therapeutic relevance of CD47/αvβ3 coexpression, we turned to the extensively characterized TNBC model MDA‐MB‐231. Flow cytometric profiling revealed that CD47 is expressed at similarly high levels on both MDA‐MB‐231 cells and mature erythrocytes (90.2 ± 3.3% vs 95.9 ± 2.5%, *p* > 0.05, Student's *t*‐test), consistent with its ubiquitous role in mediating “self” recognition. However, integrin αvβ3 was significantly underrepresented on erythrocytes relative to MDA‐MB‐231 cells (5.12 ± 1.35% vs 81.4 ± 2.7%, *p* < 0.0001; Figure 1B,C). These findings provide compelling evidence that therapeutic strategies aimed at disrupting the CD47/αvβ3 axis on malignant cells may effectively curtail tumor progression while preserving the CD47–SIRPα immune checkpoint that governs erythrocyte clearance and prevents immunotoxicity.

To investigate the presence of this correlation in human tumor tissues, we conducted multiplexed immunofluorescence staining of 119 clinical samples from breast cancer patients, with 32 cases of paraneoplastic/normal tissues serving as controls. The staining employed 520 nm wavelength green fluorescence for αvβ3‐positive (αvβ3^+^) cells, 570 nm red fluorescence for CD47‐positive (CD47^+^) cells, 650 nm sky blue for CD68‐positive (CD68^+^) macrophages, and 690 nm grayish purple for CK‐positive (CK^+^) adenocarcinoma cells (Figure 1D, during processing, 8 paracarcinoma/normal tissue samples were detached; Figure S2A, Supporting Information). Given the scarcity of clinically accessible TNBC tissue samples, this study concentrated on a heterogeneous collection of breast cancer cell lines without comprehensive characterization of their hormonal receptor (ER/PR) and HER2 expression profiles. Remarkably, the strong correlation in surface expression levels of integrin αvβ3 and CD47 identified in TNBC cell lines such as MDA‐MB‐231, Hs578T, and MDA‐MB‐468 was also evident in other subtypes, including MCF‐7 (ER⁺/PR⁺/HER2−) and MDA‐MB‐453 (ER−/PR−/HER2⁺) (see below and Figures S4, S5 in the Supporting Information).

In the 119 clinically validated breast cancer samples (Figure 1E–G), the coexpression of αvβ3 and CD47 (αvβ3^+^/CD47^+^, 0.61 ± 0.33) was more prevalent in tumor parenchymal cells (4′, 6‐diamidino‐2‐phenylindole^+^ (DAPI^+^)/CK^+^) compared to tumor mesenchymal cells (DAPI^+^/CK^−^, 0.30 ± 0.26) (*p* < 0.01, Student's *t*‐test). Despite the significant tumor heterogeneity among these samples, a majority of αvβ3^+^ cells overlapped with CD47^+^ cells in the tumor tissues of G15 (with more parenchymal but less interstitial tissue) (Figure 1E), G11 (almost entirely with tumor parenchyma) (Figure S2B, Supporting Information), and F12 (with less tumor parenchyma but more interstitial tissue (Figure S2C, Supporting Information). Moreover, within the αvβ3^+^/CD47^+^ tumor cells, αvβ3^+^ green fluorescence and CD47^+^ red fluorescence were evenly distributed around the nucleus (DAPI^+^ blue; Figure 1E; Figure S2B, Supporting Information) with clear overlap, demonstrating a clear correlation between the expression of αvβ3 and CD47 in the patient's tumor cells.

By contrast, the levels of integrin αvβ3 and CD47 were relatively low in the 30 samples identified as paraneoplastic or normal tissue (Figure 1G; Figure S2D, Supporting Information). In some tissue samples clinically proven to be inflammation rather than breast cancer samples (J07), expression of integrin αvβ3^+^ (520 nm green fluorescence) or CD47^+^ (570 nm red fluorescence) was observed, with no appreciable colocalization around the nucleus (Figure S2E, Supporting Information). The average ratio of αvβ3^+^/CD47^+^ cells in all normal or paracancerous tissues was 0.23 ± 0.25 (*n* = 32), significantly lower than the proportion of αvβ3^+^/CD47^+^ cells in tumor parenchymal cells (0.61 ± 0.33, *n* = 119, *p* < 0.01, Student's *t*‐test). These findings collectively demonstrate that αvβ3^+^ and CD47^+^ expressions are closely associated only in parenchymal cells of tumor tissues, not in normal or paracancerous tissues.

### 2.2 Decreasing Surface Expression of αvβ3 or CD47 Leads to a Substantial Decrease of the Other in Breast Carcinoma Cells

**Figure 2:**
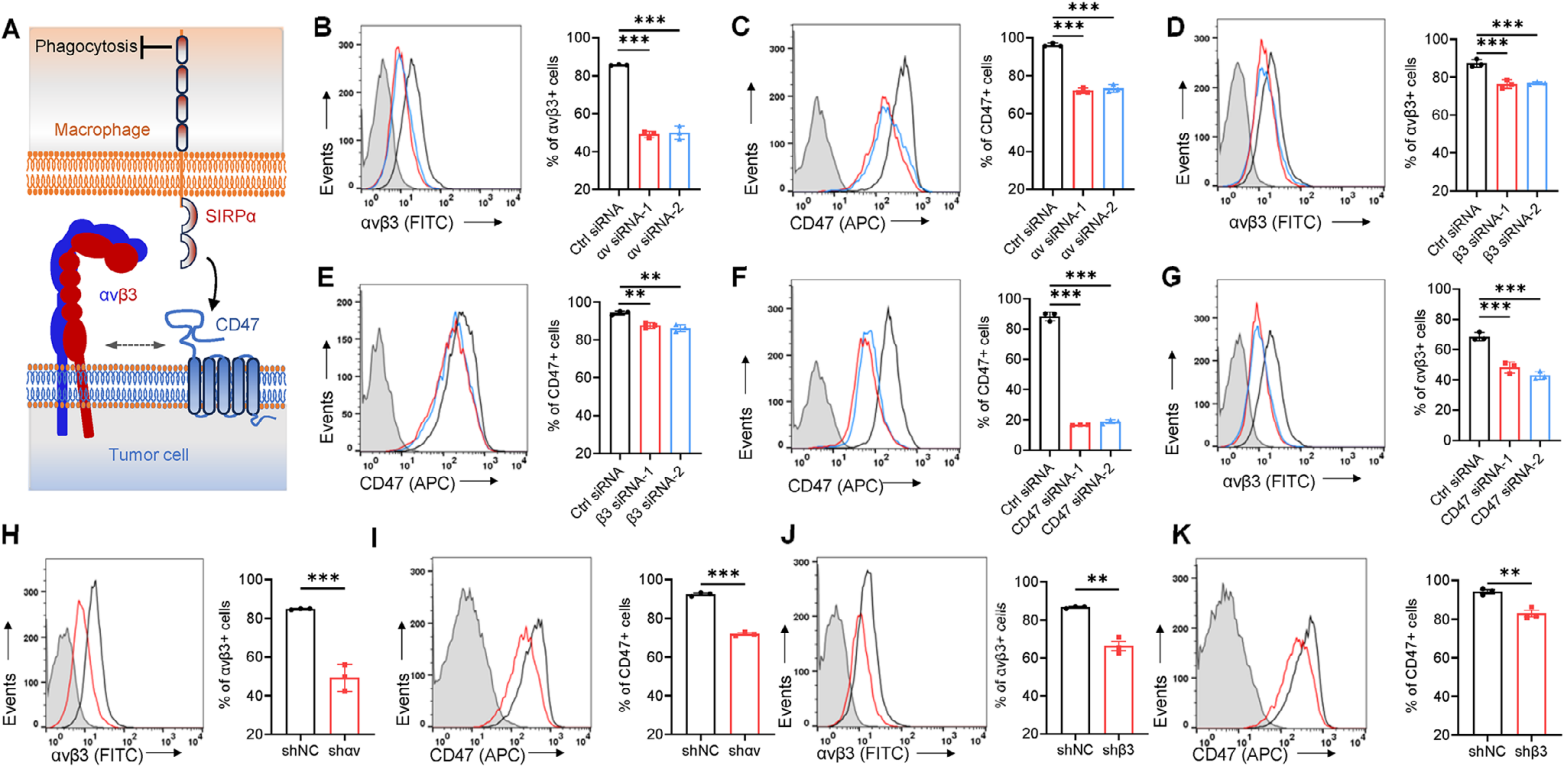
Decreased surface expression of αvβ3 or CD47 significantly reduces the presence of the other on the cancer cell surface. A) Schematic representation of αvβ3 involvement in the CD47–SIRPα axis on the cell surface. B–E) MDA‐MB‐231 cells were transfected with siRNA targeting integrin subunit αv (B,C) or β3 (D,E) for 48 h. Flow cytometry was then performed, and pooled data for cell surface markers αvβ3 and CD47 were analyzed. F,G) Flow cytometry analysis of MDA‐MB‐231 cells transfected with siRNA targeting CD47 for 48 h, followed by pooled data analysis of cell surface markers αvβ3 and CD47. H–K) MDA‐MB‐231 cells were transfected with lentiviruses targeting integrin subunit αv (H, I) or β3 (J, K) to generate stable cell lines. Flow cytometry was performed, and pooled data for cell surface markers αvβ3 and CD47 were analyzed. All data are expressed as mean ± s.e.m., *n* = 3 independent experiments; ^**^ *p* < 0.01, ^***^ *p* < 0.001 versus control, unpaired ‐test (H–K); one‐way ANOVA with Dunnett's post‐hoc test (B–G); (B), (*F* (2, 6) = 264.1, *p* < 0.0001), (C), (*F* (2, 6) = 244.7, *p* < 0.0001), (D), (*F* (2, 6) = 34.96, *p* = 0.0005, (E), (*F* (2, 6) = 25.27, *p* = 0.0012), (F), (*F* (2, 6) = 59.25, *p* = 0.0001), (G), (*F* (2, 6) = 1497, *p* < 0.0001).

To explore the correlation between αvβ3/CD47 distribution on the membrane and function (**Figure** **2**A), we initially focused on TNBC cells, particularly MDA‐MB‐231, which exhibit high expression levels of both αvβ3 and CD47 (Figure 1A). We transiently knocked down αv or β3 subunits with small interfering RNAs of αv (αv siRNA‐1 and ‐2) and β3 (β3 siRNA‐1 and ‐2) (mRNA knockdown efficiency > 85%; Figure S3A,B, Supporting Information). Knocking down the αv subunit with αv siRNA‐1 and ‐2 resulted in a decrease in αvβ3 expression on the membrane from 85.7 ± 0.3% to 49.1 ± 1.7% and 50.0 ± 3.5%, respectively, along with the corresponding decrease in CD47 expression from 96.2 ± 1.0% to 72.1 ± 1.4% and 73.3 ± 2.0%, respectively (*p* < 0.0001 for both, one‐way ANOVA with Bonferroni post‐hoc test; Figure 2B,C). When the β3 subunit was knocked down (Figure 2D,E), the expression level of αvβ3 on the membrane decreased from 87.3 ± 2.0% to 76.3 ± 2.3% and 76.8 ± 0.8% by β3 siRNA‐1 and ‐2, respectively (*p* < 0.001, *F* (2,6) = 34.96), while the CD47 expression levels decreased from 94.3 ± 1.1% to 87.6 ± 1.5% and 86.2 ± 1.8%, respectively, *p* = 0.0012). Notably, transient knockdown of the αv subunit alone or concomitant knockdown of αvβ3 (see below) had a greater effect on CD47 than transient knockdown of β3. The αvβ6 heterodimer on MDA‐MB‐231 cell membranes may also contribute to stabilizing CD47 surface expression, potentially explaining the differential effects observed between αv and β3 subunit knockdowns (see below). Additionally, transient knockdown of CD47 in MDA‐MB‐231 cells significantly reduced CD47 surface expression (*p* = 0.0001, Figure 2F), concomitant with a decrease in αvβ3 expression (*p* < 0.0001, Figure 2G).

Further investigation into the correlation between αvβ3 and CD47 expressions was conducted in stable αv^knockdown(KD)^ or β3^KD^ cell lines derived from MDA‐MB‐231, along with cells with a double knockdown of αvβ3 (αv^KD^/β3^KD^) using lentiviral expression vectors (Figure S3C, Supporting Information). In stable αv^KD^ or β3^KD^ cells, reduced αv and/or β3 expression led to a significant decrease in CD47 protein expression (*p* < 0.01 or 0.001 vs control, Student's *t*‐test; Figure S3E,F, Supporting Information). Flow cytometry analysis revealed varied reductions in surface expression of integrin αvβ3 and CD47 in MDA‐MB‐231 cells following shRNA knockdown of αv, β3, and both: knockdown of αv decreased the percentage of αvβ3^+^ cells from 84.7 ± 0.3% to 49.3 ± 6.9% (*p* = 0.0009, Figure 2H), knockdown of β3 decreased the percentage of αvβ3^+^ cells from 86.6 ± 0.7% to 66.5 ± 4.3% (*p* = 0.0013, Figure 2J), and double knockdown of αvβ3 decreased the percentage of αvβ3^+^ cells from 81.3 ± 2.5% to 42.9 ± 4.6% (*p* = 0.0002, Student's *t*‐test; Figure S3G, Supporting Information). Similar reduction in CD47 expression was also observed, with the percentage of CD47^+^ cells decreasing from 92.4 ± 1.0% to 71.9 ± 1.1% (*p* < 0.0001, Figure 2I) for αv knockdown, from 94.1 ± 1.8% to 82.9 ± 2.9% (*p* = 0.0046, Figure 2K) for β3 knockdown, and from 91.6 ± 3.4% to 67.5 ± 3.4% (*p* = 0.0009, Figure S3H, Supporting Information) for αvβ3 double knockdown (Student's *t*‐test). These results suggest a connection among surface expressions of αv, β3, and CD47, and interfering the expression of one would sequentially affect the expression of the other two.

These findings were further validated in MCF‐7 (ER^+^/PR^+^/HER2^−^) and MDA‐MB‐453 (ER^−^/PR^−^/HER2^+^), where decreased CD47 expression on the cell surface and/or total expression was observed (Figures S4C,E,G,I,K, S5B,C and Table S1 (using siRNA (Figure S4A–C) or shRNA (Figures S4D–K and S5A), Supporting Information); *p* < 0.05, *p* < 0.01, or *p* < 0.001 vs control, one‐way ANOVA with Bonferroni post‐hoc test or Student's *t*‐test). Transient knockdown of αv in other malignant cells, such as colon cancer cells HCT116, non‐small‐cell lung cancer A549, and glioma cells U87, also resulted in a significant decrease in the expression of αvβ3 and CD47 on the membrane of the cancer cells (*p* < 0.01 or *p* < 0.001 compared to control, Student's *t*‐test; Figure S5D–F, Supporting Information). These results underscore a connection between αvβ3 and CD47 in tumor cells, where interfering with the surface expression of one leads to a corresponding change in the other.

### 2.3 αvβ6, Although Expressed in Lower Abundance Compared to αvβ3, Plays a Role in Stabilizing CD47 Expression on Cancer Cell Surfaces

Notably, knocking down the αv subunit resulted in a more pronounced reduction in CD47 surface expression than knocking down the β3 subunit, suggesting the involvement of other integrins that interact with αv to form heterodimers (Figure S6A, Supporting Information) and contribute to CD47 stabilization in cancer cells.^[^ ^19^ ^]^ To explore this further, we examined the gene expression of several integrins (ITGA2B, ITGA5, ITGB1, ITGB6, and ITGB8) in MDA‐MB‐231 cells, revealing relatively high expression levels of ITGA5, ITGB1, and ITGB6 (Figure S6B, Supporting Information). Analysis using the String database indicated potential associations of CD47 with other proteins such as SIRPα, TSP‐1, and integrins αv, β3, and αIIb (Figure S7A,B, Supporting Information). However, the weak association of CD47 with α5, β1, and β6 indicated potential undisclosed interactions. Thus, we investigated the effects of α5, β1, and β6 on CD47 expression in MDA‐MB‐231 cells.

Flow cytometry analysis following transient knockdown of α5, β1, and β6 using siRNAs in MDA‐MB‐231 cells revealed decreased expressions of αvβ3 and CD47 specifically upon knocking down β6 (*p* = 0.001 vs control siRNA; Figure S6F–L, Supporting Information). By contrast, knocking down α5 or β1 had no significant effect on αvβ3 or CD47 expression levels (*p* > 0.05, Student's *t*‐test; Figure S6H–K, Supporting Information). Additionally, knockdown of the β6 subunit also led to reduced αv expression (*p* = 0.0006, Student's *t*‐test; Figure S6L, Supporting Information), while knockdown of α5 or β1 had no significant effect on αv expression (*p* > 0.05, Figure S6M,N, Supporting Information). Similarly, knocking down the β3 subunit led to reduced αv subunit expression (*p* = 0.0040; Figure S6O,P, Supporting Information). Thus, β3 and β6 interact with αv, forming dimers on the cancer cell membrane, thereby impacting CD47 expression. However, given the significantly higher mRNA levels of β3 compared to β6 in MDA‐MB‐231 cells (Figure S6B, Supporting Information), it is likely that a larger proportion of αvβ3 dimers coexist with CD47.

Corroboratively, reverse transcriptase‐q‐polymerase chain reaction (RT‐qPCR) across multiple cancer cell lines revealed poor correlation between mRNA abundance of ITGAV, ITGB3, and CD47 and their respective surface localization (Figure S7C, Supporting Information), suggesting significant posttranscriptional and posttranslational regulatory influences. Moreover, CD47 silencing did not impact membrane levels of integrin αLβ2 (CD11a/CD18) (Figure S7D, Supporting Information), despite its known interaction with CD47,^[^ ^43^ ^]^ further underscoring the complexity of integrin–CD47 regulatory dynamics beyond transcriptional control.

### 2.4 The Interaction between CD47 and αvβ3 on Cancer Cell Surfaces Plays a Pivotal Role in the Tumor–Macrophage CD47/SIRPα Immune Checkpoint

**Figure 3:**
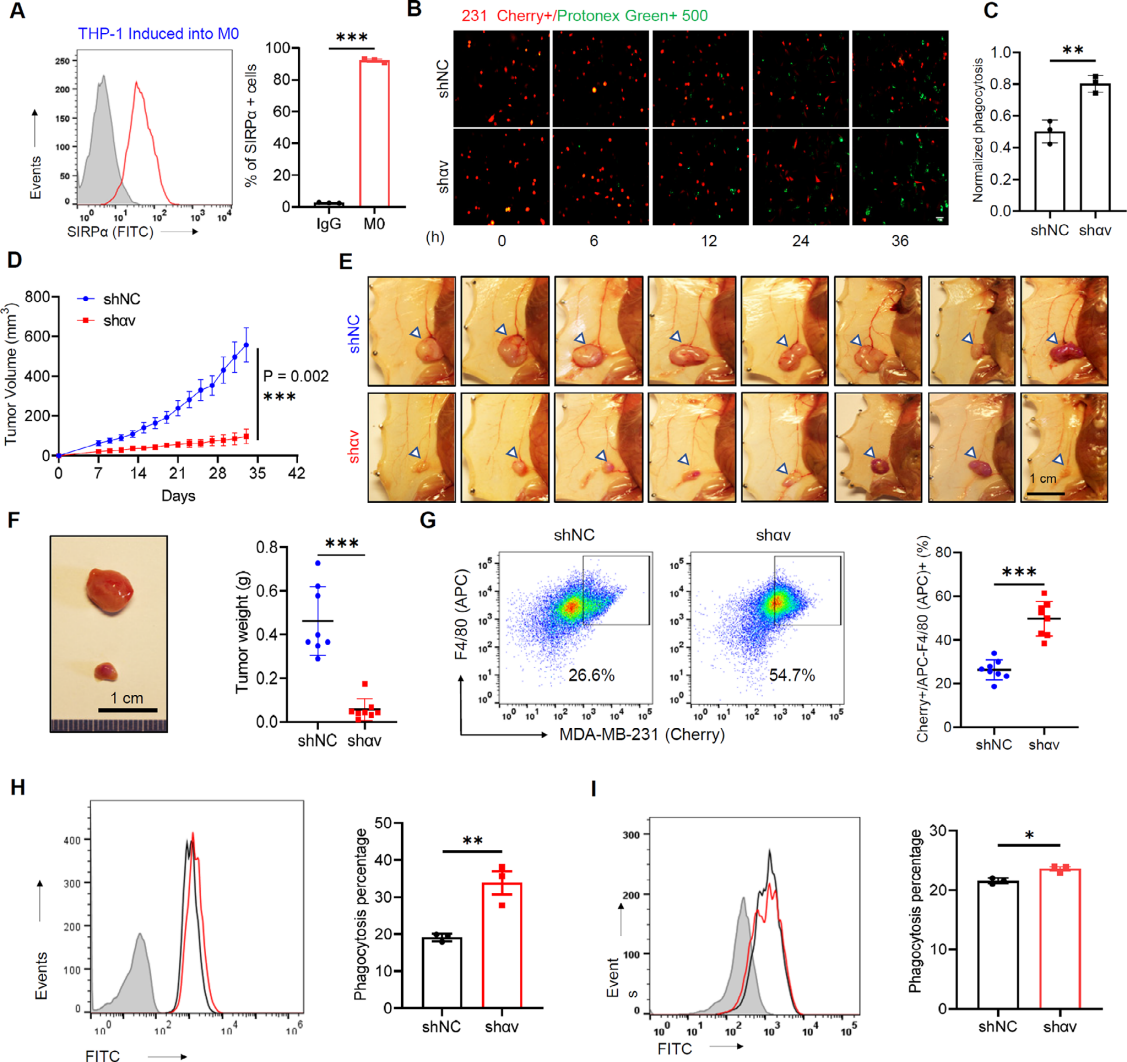
Knockdown of integrin αv expression decelerates orthotopic tumor growth in the mammary fat pad of nude mice. A) THP‐1‐derived macrophages were labeled and analyzed by flow cytometry for SIRPα expression over 48 h, with pooled data for the cell surface marker SIRPα. B,C) siRNA‐mediated knockdown of the integrin αv subunit enhances phagocytosis by THP‐1‐derived macrophages on MDA‐MB‐231‐Cherry cells. (B) Representative fluorescence microscopy images of phagocytic activity and (C) quantitative analysis of the mean fluorescence intensity signal in confocal images. Scale bar = 50 µm. D) MDA‐MB‐231 shNC or MDA‐MB‐231 shαv tumor cells (2 × 10^6^) were implanted in the mammary fat pad of female nude mice. Tumor growth kinetics were compared over 33 days (8 mice per group). E) Representative images of mice transplanted with MDA‐MB‐231 shNC versus MDA‐MB‐231 shαv tumors on day 33 (images represent two independent experimental groups, 8 mice per group). Scale bar = 1 cm. F) Representative images of tumor size and tumor weight analysis of MDA‐MB‐231 shNC and MDA‐MB‐231 shαv tumors. Scale bar = 1 cm. G) Representative flow cytometry plots showing macrophage phagocytosis of Cherry+ MDA‐MB‐231 shNC tumors (left) versus MDA‐MB‐231 shαv tumors (middle). Numbers indicate the frequency of phagocytic events in all macrophage infiltrates. Quantification of all macrophage infiltrates (right) was assessed for MDA‐MB‐231 (Cherry)+/F4/80 (APC)+. Eight experimental replicates are represented. H,I) Flow cytometric analysis of phagocytic activity by bone‐marrow‐derived (H) and primary peritoneal (I) macrophages from NSG mice toward ITGAV‐stably silenced MDA‐MB‐231 cells and control counterparts. All data are expressed as mean ± s.e.m.; *p < 0.05, ^**^ *p* < 0.01, ^***^ *p* < 0.001 versus control, unpaired *t*‐test (A, C, D, F–I).

CD47, highly expressed on tumor cells, binds to SIRPα on macrophages, forming CD47–SIRPα immune checkpoints that facilitate tumor evasion from macrophage phagocytosis.^[^ ^2^ ^]^ Given this, we hypothesized that the concomitant presence of CD47 and αvβ3 on cancer cell membranes might have functional implications for the CD47–SIRPα axis. To explore this hypothesis, we cocultured phagocytic macrophages (M0) and MDA‐MB‐231 cells and analyzed the effect of M0 phagocytosis of tumors. Differentiation of human monocytes THP‐1 and peripheral mononuclear blood cells (PBMCs) into macrophages M0 and Mφ, respectively, was induced using phosphomycin (PMA) and human macrophage colony‐stimulating factor (hM‐CSF) (**Figure** **3**A; Figure S8A (Supporting Information)).The percentage of SIRPα^+^ cells in both M0 and Mφ cells was significantly increased (*p* < 0.0001, 2.60 ± 0.3% vs 92.1 ± 1.1%, IgG vs M0 (induced by THP‐1); *p* < 0.0001, 1.95 ± 0.8% vs 89.9 ± 1.6%, IgG vs Mφ (induced by PBMC), Student's *t*‐test; Figure 3A; Figure S8A, Supporting Information), indicating sufficient SIRPα production to establish a CD47–SIRPα axis with CD47 on tumor cell membranes. Given the superior receptiveness of THP‐1 cells over human‐blood‐derived ones, the THP‐1–M0 system was chosen for tumor cell coculture in further experiments. Notably, M0 cells possibly require a prolonged coculture duration to achieve tumor cell phagocytosis (following 6 h of coculturing, discernible alterations in phagocytic activity compared to the shNC group are observed, with these changes becoming increasingly pronounced over time (see below)), compared to Mφ cells.

Then, in coincubation experiments with M0 macrophages and tumor cells, we prelabeled MDA‐MB‐231‐Cherry+ (red) shNC control cells and cells knocked down for αv or β3 with a pH‐sensitive green fluorescent dye (protonex Green 500) to measure phagocytosis efficiency (the green dye is also conveniently distinguishable from Cherry+ red cells). A significant increase in green fluorescence was observed in the coincubation group with αv knockdown compared to the control group (shNC) (shNC vs shαv, *p* = 0.0043, Student's *t*‐test; Figure 3B,C), suggesting that MDA‐MB‐231‐Cherry+ cells are more susceptible to phagocytosis by macrophages when αv is knocked down, possibly due to a decrease in CD47 surface expression and the subsequent recognition of cancer cells as phagocytic targets. Furthermore, knockdown of β3 (by shβ3) or double knockdown of αvβ3 (by shαvβ3) in MDA‐MB‐231 cells also increased the proportion of tumor cells phagocytosed by macrophages (shNC vs shβ3, *p* = 0.037; shNC vs shαvβ3, *p* = 0.0013, Student's *t*‐test; Figure S8B–E, Supporting Information). These findings support the notion that reduced expression of αv and β3 integrins could enhance the phagocytosis activity of macrophages toward cancer cells.

Moving forward, we examined the roles of αvβ3 integrins on the phagocytic activity of macrophages in the MDA‐MB‐231 orthotopic tumor model. Since αv knockdown caused a more pronounced reduction in CD47 when compared to β3 knockdown (Figure 2I,K), we implanted MDA‐MB‐231 shNC or MDA‐MB‐231 shαv tumors in the mammary fat pad of nude mice and evaluated tumor growth on day 33 postimplantation. The αv knockdown group exhibited significantly decelerated tumor growth (*p* = 0.002, tumor volume of shαv vs shNC, Student's *t*‐test; Figure 3D,E) and a considerable reduction in tumor weight when compared to the shNC group (*p* < 0.0001, shNC vs shαv, Student's *t*‐test; Figure 3F).

Moreover, the percentage of infiltrating macrophages in tumor tissue was significantly increased in the shαv group (Cherry+/F4/80+: shNC vs shαv, *p* < 0.0001, Student's *t*‐test; Figure 3G), which enhanced phagocytosis of tumor cells (*p* < 0.0001, Cherry+/CD11b+: shNC vs shαv, Figure S8F, Supporting Information), contributing to the reduced tumor size observed in the shαv group. Additionally, a higher level of macrophage infiltration was observed in the peripheral blood of the shαv group (*p* < 0.0001, shNC vs shαv; Figure S8G, Supporting Information), implying activation of the monocyte–macrophage system in the peripheral blood after αv knockdown.

While murine SIRPα exhibits a recognition profile for human CD47 distinct from that of human SIRPα, a measurable degree of cross‐species interaction persists.^[^ ^44^ ^]^ To interrogate this phenomenon, primary murine bone marrow cells were differentiated into macrophages using M‐CSF and subjected to phagocytosis assays. ITGAV knockdown in MDA‐MB‐231 cells resulted in significantly increased phagocytic uptake by these macrophages (*p* < 0.01, unpaired *t*‐test; Figure 3H). A comparable enhancement in phagocytosis was observed with primary murine peritoneal macrophages (*p* < 0.05; Figure 3I), validating the role of ITGAV in tumor immune evasion mechanisms. These results highlight the functional interplay between αv integrins and CD47 in modulating immune surveillance across species. Furthermore, disruption of the CD47/αvβ3–SIRPα signaling in human‐tumor‐bearing NSG mice led to attenuated tumor growth and significantly prolonged survival, thereby providing further evidence of the pivotal role that membrane colocalization and structural integrity of CD47/αvβ3–SIRPα complexes play in tumor immune escape (see below).

### 2.5 Perturbing CD47 and αvβ3 Costabilization on Cancer Cell Surface by Antibodies and Small Molecules

Subsequently, we pursued alternative strategies, including antibodies or small molecules, to disrupt the costabilization of CD47 and αvβ3. MDA‐MB‐231 cells were treated with CD47 antibody (B6H12) or antibodies against αv (Arg108) or β3 (Ile114) for 48 h. Subsequently, the tumor cells were labeled with the pH‐sensitive red fluorescent dye protonex Red 600 and cocultured with macrophages, after which the average red fluorescence intensity was quantified. Notably, the CD47 antibody (B6H12) significantly increased phagocytosis of MDA‐MB‐231 cells by macrophages, demonstrating approximately a twofold rise compared to the IgG control group (phagocytosed/total: B6H12 vs control, *p* < 0.001, Student's *t*‐test; Figure S9A,B, Supporting Information). Similarly, antibodies targeting αv (Arg108) or β3 (Ile114) alone also led to a significant increase in red fluorescence associated with phagocytosis, indicating their potential to disrupt the CD47–αvβ3 costabilization (Arg108 or lle114 vs control, *p* < 0.05, Student's *t*‐test; Figure S9C–F, Supporting Information).

**Figure 4:**
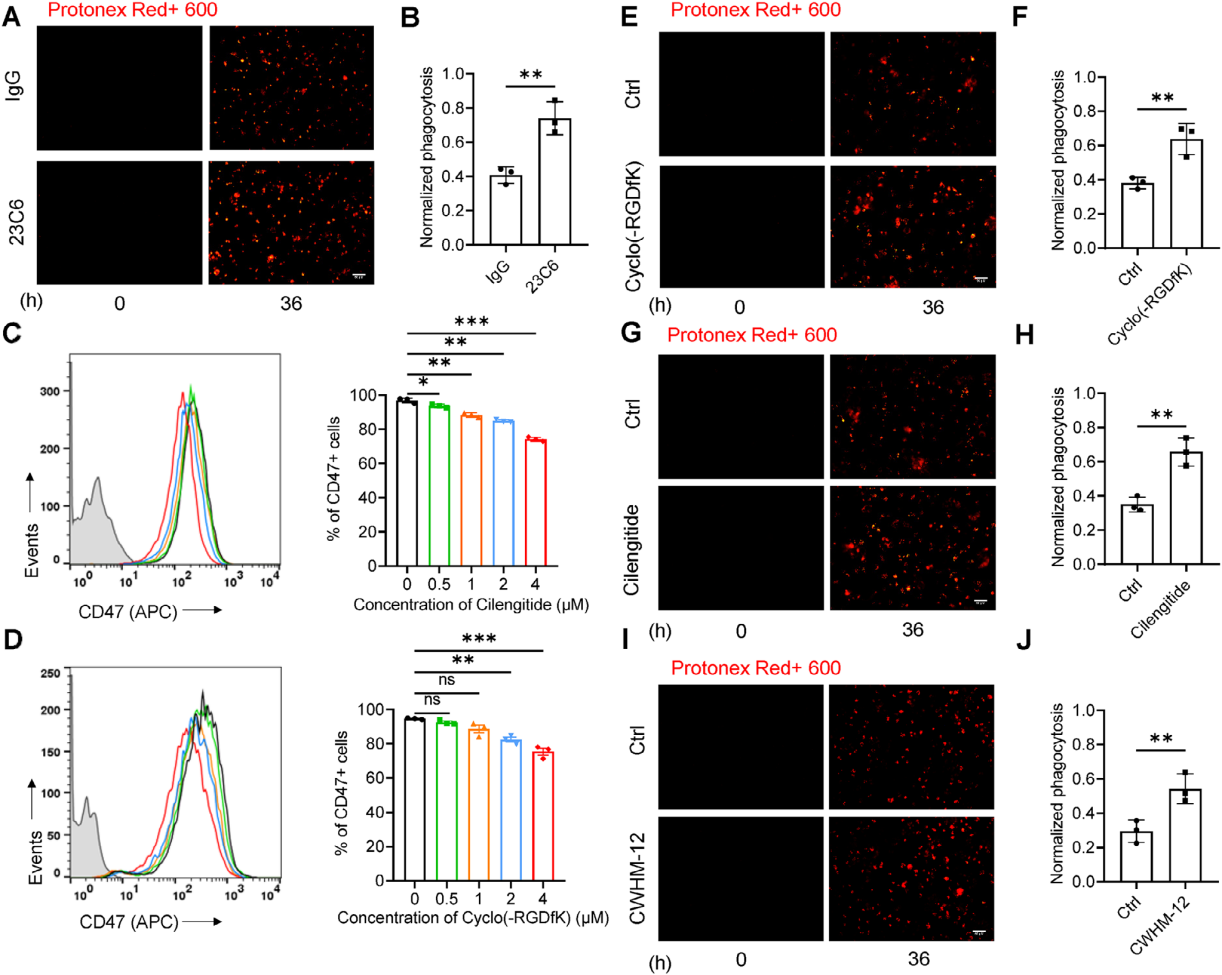
Integrin αvβ3‐specific inhibitors reduce CD47 expression and enhance macrophage‐mediated tumor phagocytosis. A,B) Representative fluorescence microscopy images of MDA‐MB‐231 cell phagocytosis by macrophages (Protonex Red+, red) after 36 h of coculture with either IgG control or integrin αvβ3 activating antibody (23C6, 10 µg mL^−1^) (A), and quantitative analysis of mean fluorescence intensity in confocal images (B). Scale bar: 50 µm. C,D) Flow cytometry analysis of CD47 surface expression in MDA‐MB‐231 cells treated with various concentrations of the integrin αvβ3 inhibitors cilengitide (C) or cyclo(─RGDfK) (0, 0.5, 1, 2, and 4 µm) (D) for 48 h, including pooled data. E–H) Representative images from live cell microscopy phagocytosis assays of Protonex Red+ MDA‐MB‐231 cells treated with the integrin αvβ3 inhibitor cyclo(─RGDfK) (E) or cilengitide (4 µm) (G) for 36 h, and quantitative analysis of mean fluorescence intensity in confocal images (F, H). Scale bar: 50 µm. I,J) Representative images from live cell microscopy phagocytosis assays of Protonex Red+ MDA‐MB‐231 cells treated with the integrin αv inhibitor CWHM‐12 (4 µm) (I), and quantitative analysis of mean fluorescence intensity in confocal images (J). Scale bar: 50 µm. All data are expressed as mean ± s.e.m., *n* = 3 independent experiments; ^*^ *p* < 0.05, ^**^ *p* < 0.001, and ^***^ *p* < 0.001 versus control, unpaired *t*‐test (B, F, H and J); one‐way ANOVA with Dunnett's post‐hoc test (C and D); (C), (*F* (4, 10) = 162.8, *p* < 0.0001), (D), (*F* (4, 10) = 25.18, *p* < 0.0001); ns, not significant.

To validate this phenomenon, we individually treated MDA‐MB‐231 cells with the CD47 antibody (B6H12) (Figure S9A,H, Supporting Information) and an antibody targeting αvβ3 (23C6), as well as their combination, for 48 h, followed by labeling with pHrodo red dye and flow cytometry analysis (**Figure** **4**A; Figure S9G, Supporting Information). Both B6H12 and 23C6, whether used alone or combined, significantly boosted macrophage‐mediated phagocytosis efficiency of tumor cells by 4.18‐fold, 3.85‐fold, and 5.89‐fold, respectively (B6H12 23C6, B6H12 + 23C6 vs control, *p* < 0.001, Student's *t*‐test; Figure S9G–I, Supporting Information). Notably, the CD47 antibody B6H12, known for its interference with the CD47/SIRPα axis, further enhanced phagocytosis when combined with the αvβ3 antibody 23C6 (B6H12 vs B6H12 + 23C6, *p* < 0.001, Student's *t*‐test; Figure S9I, Supporting Information), indicating the involvement of αvβ3 in the regulation of the tumor–macrophage CD47/SIRPα axis.

Synthetic peptides or small molecules, such as cyclo(─RGDfK) and cilengitide, have been developed to target αvβ3 specifically. In our evaluation of their effects, MDA‐MB‐231 cells were treated with various concentrations of cyclo(─RGDfK) and cilengitide (0, 0.5, 1, 2, and 4 µm) for 48 h. Neither cyclo(─RGDfK) nor cilengitide demonstrated a direct inhibitory effect on the proliferation of MDA‐MB‐231 cells when compared to the control group (*p* > 0.05 vs control, one‐way ANOVA with Bonferroni post‐hoc test; Figure S10A,B, Supporting Information). However, at a concentration of 4 µm for 48 h, both cyclo(─RGDfK) and cilengitide decreased the total protein expression levels of αvβ3 and CD47 (*p* < 0.05, Student's *t*‐test; Figure S10C,D, Supporting Information). Subsequent analysis via flow cytometry revealed that both peptides could reduce the expression of αvβ3 on the membranes of MDA‐MB‐231 cells in a concentration‐dependent manner (*p* < 0.05, 0.01, or 0.001, one‐way ANOVA with Bonferroni post‐hoc test; Figure S10E,F, Supporting Information), as well as that of CD47 (*p* < 0.01 or 0.001, for cyclo(─RGDfK) and cilengitide, one‐way ANOVA with Bonferroni post‐hoc test; Figure 4C,D).

Meanwhile, following the treatment of MDA‐MB‐231 cells with 4 µm cyclo(─RGDfK) and cilengitide for 48 h, MDA‐MB‐231 cells were labeled with the pH‐sensitive dye protonex Red 600 and cocultured with macrophages. This led to a significant increase in macrophage‐mediated phagocytosis of cancer cells compared to the control group (*p* < 0.01 vs controls, Student's *t*‐test; Figure 4E–H). Additionally, the small molecule inhibitor CWHM‐12, which selectively targets the αv subunit, also significantly enhanced phagocytosis of tumor cells by macrophages (*p* < 0.01 vs control, Student's *t*‐test; Figure 4I,J). These results support the hypothesis that the αv subunit is the primary mediator of αvβ3‐mediated regulation of CD47 expression.

The costabilization of CD47 and integrin αvβ3 at the plasma membrane of malignant cells constitutes a functionally significant and therapeutically tractable interaction. Beyond gene interference approaches such as siRNA or shRNA, pharmacological disruption of the CD47/αvβ3 axis using monoclonal antibodies, peptide inhibitors, or small‐molecule compounds might offer a promising avenue to potentiate macrophage‐driven phagocytosis of malignant cells, thereby advancing strategies aimed at overcoming tumor immune evasion.

### 2.6 The Dominant Conformers of αvβ3, Coexisting Stably with CD47 on Cancer Cell Surfaces, May Represent Specific Conformations Distinct from the Inactivated and Activated States

**Figure 5:**
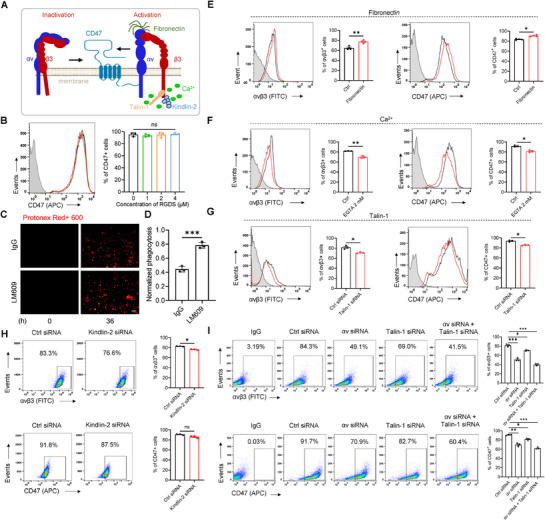
Blocking αvβ3 activation reduces surface expression of αvβ3 and CD47 on MDA‐MB‐231 cells. A) Schematic representations of the activated and inactive states of integrin αvβ3. B) Flow cytometry analysis of CD47 surface expression on MDA‐MB‐231 cells treated with various concentrations of the integrin αvβ3 inhibitor RGDS (0, 1, 2, and 4 µm) for 48 h, including pooled data. C,D) Representative fluorescence microscopy images of in vitro phagocytosis of MDA‐MB‐231 cells by macrophages (Protonex Red+, red) after 36 h of coculture with either IgG control or the integrin αvβ3 nonactivating antibody (LM609, 10 µg mL^−1^) (C), and quantitative analysis of mean fluorescence intensity in confocal images (D). Scale bar: 50 µm. E,F) Flow cytometry analysis of αvβ3 and CD47 surface expression on MDA‐MB‐231 cells treated with fibronectin (FN, 20 µg mL^−1^) (E) or EGTA (2 mm) (F) for 48 h, with pooled data of surface markers αvβ3 and CD47. G) Flow cytometry analysis of αvβ3 and CD47 surface expression on MDA‐MB‐231 cells after siRNA‐mediated knockdown of Talin‐1 for 48 h, including pooled data of surface markers αvβ3 and CD47. H) Flow cytometry analysis of αvβ3 and CD47 surface expression in MDA‐MB‐231 cells 48 h after siRNA‐mediated knockdown of Kindlin‐2, with pooled data of surface markers αvβ3 and CD47. I) Flow cytometry analysis of αvβ3 and CD47 surface expression in MDA‐MB‐231 cells 48 h after combined siRNA‐mediated knockdown of the αv subunit and Talin‐1, with pooled data for surface markers αvβ3 and CD47. All data are expressed as mean ± s.e.m., *n* = 3 independent experiments; ^*^ *p* < 0.05, ^**^ *p* < 0.001, ^***^ *p* < 0.001 versus control, unpaired *t*‐test (D–H); one‐way ANOVA with Dunnett's post‐hoc test (B and I); (B), (*F* (3, 8) = 0.4324, *p* = 0.7356), (I), (*F* (3, 8) = 134.3, *p* < 0.0001), (*F* (3, 8) = 71.77, *p* < 0.0001). ns, not significant.

Unveiling the precise state of αvβ3 interaction with CD47 is crucial for developing immune checkpoint therapies targeting their mutually stable coexistence. Integrin αvβ3 heterodimers exhibit dynamic fluctuations between a low‐affinity bent‐closed (inactivated) conformation and a high‐affinity extended open (activated) conformation.^[^ ^45^ ^]^ This transition, defined as integrin activation (**Figure** **5**A; Figure S11A,B, Supporting Information), is accompanied by the formation of multiple intermediate conformers (Figure S11C, Supporting Information). Notably, antibodies like 23C6, potentially targeting the αvβ3 activation state,^[^ ^46^, ^47^ ^]^ and αvβ3 inhibitors such as peptides cyclo(─RGDfK) and cilengitide, significantly interfere with the mutually stable coexistence of αvβ3 and CD47 on cancer cell surfaces (Figure 4; Figure S10, Supporting Information). These results imply that αvβ3 primarily interacts with CD47 on cancer cell surfaces when activated.

To further confirm these assumptions, MDA‐MB‐231 cells were incubated with varying concentrations of the αvβ3 inhibitor peptide RGDS (Figure S12A,B, Supporting Information), which competes with extracellular RGD‐sequence‐containing αvβ3 activators like fibronectin (FN).^[^ ^48^ ^]^ RGDS (0.5, 1, 2, and 4 µm) showed no direct effect on cell proliferation or αvβ3 expression on cell membranes (*p* > 0.05, one‐way ANOVA with Bonferroni post‐hoc test; Figure S12B, Supporting Information). Interestingly, RGDS also did not reduce αvβ3 expression on MDA‐MB‐231 cell membranes (*p* > 0.05, one‐way ANOVA with Bonferroni post‐hoc test; Figure S12A, Supporting Information). The surface expression of CD47 was also unaffected (*p* > 0.05; Figure 5B). However, phagocytosis efficiency increased by ≈30% when using antibody LM609, which predominantly binds to nonactivated state αvβ3 (*p* < 0.01 vs control, Student's *t*‐test; Figure 5C,D), and the antibody 23C6 bound to activated‐state αvβ3 (*p* < 0.01 vs control, unpaired Student's *t*‐test; Figure 4A,B). Hence, defining the coexistence of αvβ3 and CD47 solely based on its activated conformation might be oversimplified.

Next, after treating MDA‐MB‐231 cells with 20 µg mL^−1^ αvβ3‐activating ligand fibronectin (Figure 5A) for 48 h, the surface expression of αvβ3 and CD47 was increased by ≈12% and 7%, respectively, compared to the control (*p* < 0.05, Student's *t*‐test; Figure 5E). Similarly, chelation of extracellular Ca^2+^ (associated with integrin activation^[^ ^49^ ^]^) with 2 mm ethylene glycol‐bis(*b*‐aminoethylether)‐*N*,*N*,*N9*,*N9*‐tetraacetic acid (EGTA) resulted in a decrease in surface expression of αvβ3 and CD47 by ≈12% and 9%, respectively (*p* < 0.05 vs control, Student's *t*‐test; Figure 5G,H). Additionally, the use of siRNA effectively transiently knocked down integrin‐activation‐associated scaffolding proteins Talin and Kindlin, particularly Talin, resulting in a reduction in the surface expression of αvβ3 and CD47 by ≈3–10 % (*p* < 0.05 vs control, unpaired *t*‐test; Figure 5H). Thus, these factors, namely EGTA/Ca^2+^ and Talin‐1 siRNA, contributed minimally, accounting for approximately one‐third of the observed effect (Table S2, Supporting Information), compared to the significant reduction in αvβ3 and CD47 expression on the cell surface by about 36% and 24%, respectively, resulting from the transient suppression of αv using αv‐siRNA (Figure 2B,C; Table S2, Supporting Information).

Flow cytometry analysis of αvβ3 and CD47 surface expression in MDA‐MB‐231 cells following individual knockdown of αv and Talin‐1, as well as the combined knockdown of αv and Talin‐1, yielded consistent results. Treatment with Talin‐1 siRNA led to a decrease in αvβ3 expression from 81.5 ± 3.2% to 70.8 ± 1.8%, and CD47 expression from 91.0 ± 1.4% to 81.9 ± 2.7%. Upon αv siRNA knockdown, αvβ3 expression decreased from 81.5 ± 3.2% to 50.9 ± 3.2%, and CD47 expression decreased from 91.0 ± 1.4% to 69.9 ± 3.7%. Combining αv siRNA and Talin‐1 siRNA resulted in further reductions in αvβ3 expression, from 81.5 ± 3.2% to 39.8 ± 2.8%, and CD47 expression from 91.0 ± 1.4% to 62.1 ± 2.2% (*p* < 0.05, 0.01, or 0.001, compared to control, one‐way ANOVA with Bonferroni post‐hoc test, *p* < 0.0001; Figure 5I). By contrast, flow cytometry analysis of αvβ3 and CD47 surface expression in MDA‐MB‐231 cells did not reveal statistically significant changes after knockdown of Talin‐1 and Kindlin‐2 alone, or when combined (*p* > 0.05 compared with Talin‐1 siRNA, one‐way ANOVA with Bonferroni post‐hoc test; Figure S12F,G, Supporting Information).

These findings suggest that these factors may influence the coexistence of αvβ3 and CD47 in specific conformations or dynamic transitional processes of αvβ3, rather than solely initiating a cascade of signaling events associated with activation. It is therefore plausible that the fundamental conformation in which αvβ3 and CD47 stably coexist on the surface of cancer cells resides in one or more intermediate states, likely in the form of a bent/extended closed intermediate conformation (Figure S11B,C, Supporting Information and see below). Targeting interventions specific to the predominant conformation where αvβ3 and CD47 stably coexist in tumors, without directly interfering with CD47 or αvβ3, could potentially mitigate side effects such as hemolysis or the promotion of tumor angiogenesis (see below).

### 2.7 Possible Biophysical Mechanisms by Which the CD47/αvβ3 Complex on Tumor Cells Interacts with SIRPα on Macrophages to Regulate the CD47–SIRPα Immune Checkpoint

**Figure 6:**
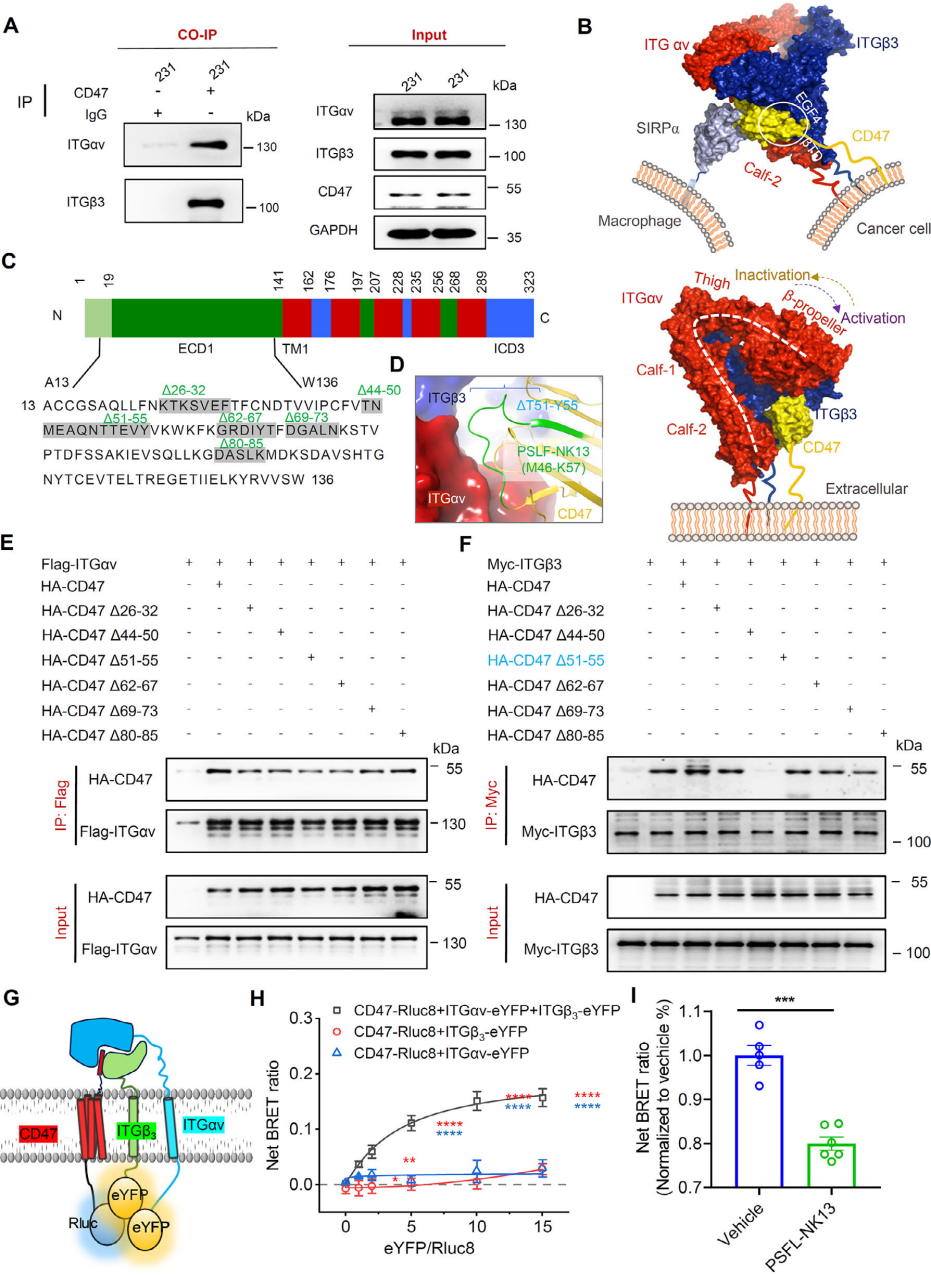
Direct interaction between CD47 and integrin αvβ3 in cancer cells. A) MDA‐MB‐231 cell lysates were immunoprecipitated with control IgG antibody and anti‐CD47 antibody. Co‐immunoprecipitation (Co‐IP) revealed the presence of ITGαv and ITGβ3 in proteins precipitated by the CD47 antibody, indicating a direct interaction between CD47 and integrin αvβ3 in CD47‐expressing MDA‐MB‐231 cells. B) Optimized model showing CD47 (yellow) interacting with integrin αvβ3 (red and blue) on the same cell, or with SIRPα (grey) on macrophages through its IgV domain. C) Schematic diagram of the extracellular region sequence of CD47. D) Optimized model of CD47 interaction with αvβ3 obtained using Z‐dock and conformation‐enhanced sampling. This model identified the T51–Y65 region as a key site in CD47 for interaction with αvβ3, leading to the design of an interfering peptide (green, PSFL‐NK13^M46‐K57^). E,F) Lysates of HEK293 cells expressing Flag–ITGαv or Myc–ITGβ3 and HA–CD47 (HA–CD47: K26–F32, T44–N50, T51–Y55, G62–T67, D69–N73, D80–K85) were analyzed. HA signal was detected in proteins precipitated with Flag or Myc antibodies. Co‐IP signal for Myc was reduced in cells coexpressing Myc–ITGβ3 and HA–CD47^Δ51–55^. These results were consistently observed in three independent experiments. G) Schematic diagram illustrating the detection of the CD47, ITGαv, and ITGβ3 ternary complex using BRET (Bioluminescence Resonance Energy Transfer). H) Regression curves presenting the net BRET signals in HEK293T cells transfected with CD47–Rluc8, ITGαv–eYFP, and ITGβ3–eYFP, *n* = 3–7; *p < 0.05, ***p < 0.001, ****p < 0.0001 when compared to BRET signals without ITGαv–eYFP or ITGβ3–eYFP, *F* (10, 66) = 6.538, Two‐way ANOVA. I) Evaluation of the impact of PSFL‐NK13 (20 µm) on the BRET signal corresponding to the CD47–Rluc8, ITGαv–eYFP, and ITGβ3–eYFP ternary complex. *n* = 5–6; ****p < 0.0001 compared to vehicle, unpaired *t*‐test. All data are presented as mean ± s.e.m.

In pursuit of strategies to specially disrupt the CD47/αvβ3 interaction, we delved into the biophysical mechanisms governing the presence of this complex on tumor cells. CD47, initially identified as an integrin‐associated protein binding to integrin αvβ3 in placental tissue and platelets,^[^ ^32^ ^]^ has raised questions regarding its interaction with αvβ3 in tumor cells. Our immunoprecipitation (IP) assays of MDA‐MB‐231 cell lysates using IgG and CD47 antibodies unveiled a robust interaction between endogenously expressed αvβ3 and CD47 protein (**Figure** **6**A). Further, co‐immunoprecipitation (Co‐IP) experiments with stable MDA‐MB‐231 cell lines featuring αv knockdown demonstrated a notable reduction in both total αvβ3 protein levels and CD47 binding compared to control shNC (Figure S13A, Supporting Information). To bolster these findings, we constructed plasmids harboring Flag‐tagged αv subunits, Myc‐tagged β3 subunits, and HA‐tagged CD47 (Figure S13B, Supporting Information), followed by their cotransfection into HEK293 cells. Subsequent immunoprecipitation with HA‐CD47 beads consistently revealed the coprecipitation of αv and β3 proteins (Figure S13C–H, Supporting Information). These collective results affirm a direct interaction between endogenously expressed CD47 and αvβ3 in MDA‐MB‐231 cells, as well as exogenously overexpressed CD47 and αvβ3 in HEK293 cells, underscoring the robustness of this association. This experimental framework provides crucial insights into the CD47/αvβ3 interaction, laying a foundation for targeted disruption strategies with potential implications in cancer therapeutics.

The extracellular region of CD47 has been identified as crucial for its interaction with αvβ3,^[^ ^32^ ^]^ prompting our focus on understanding the biophysical mechanisms underlying the mutual stability and coexistence of these proteins on cancer cell membranes. Analyzing the crystallographic structures of CD47 (PDB ID: 2JJT) and αvβ3 (PDB ID: 3IJE), we determined their spatial orientations relative to the cell membrane. Notably, the uppermost amino acid of the extracellular domain of CD47 is ≈40 Å from the cell membrane (Figure S14A, Supporting Information), while αvβ3 resides 70–110 Å away, regardless of its extended activation conformer (Figures S11B and S14A, Supporting Information). These observations suggest a specific interaction involving the αv subunit's Calf‐2 region and the βTD and EGF‐4 domains of the β3 subunit, regardless of whether αvβ3 is in the activated or inactivated state (Figures S11B and S14A, Supporting Information).

To delve deeper, we conducted an extensive computational analysis using the Z‐dock algorithm,^[^ ^50^ ^]^ incorporating structural data of the extracellular structural domain of CD47 (CD47‐ECD)/SIRPα complex (PDB ID: 2JJT)^[^ ^51^ ^]^ and the closed conformer of αvβ3 (PDB ID: 3IJE).^[^ ^52^ ^]^ Exclusion of certain αvβ3 domains distant from the membrane, such as β‐propeller, Thigh, β I‐like, PSI, etc. (Figure S14A,B, Supporting Information), yielded a plausible interaction model between macrophage SIRPα and CD47/αvβ3‐ECD on tumor cells (Figure 6B; Figure S14C, Supporting Information). This model underwent further optimization using the Replica Exchange with Solute Tempering (REST) method,^[^ ^53^, ^54^ ^]^ which helps to enhance conformational sampling over the temperature range of 300–372 K, involving 32 replicas with exchange probabilities exceeding 0.3 (Figure S15A–D, Supporting Information). Additionally, an ≈500 ns conventional molecular dynamics (CMD) simulation was performed (Figure S15D, Supporting Information). In both REST and CMD optimized models, CD47‐ECD was found to be inserted at a skewed angle into the interface formed by the Calf‐2 region of the αv subunit and the I‐EGF4 and βTD domains of the β3 subunit (Figure S15E, Supporting Information). Furthermore, SIRPα on macrophage establishes a direct contact with the CD47/αvβ3‐ECD complex on tumor cells, with an angle of ≈80°–90° (Figure 6B; Figure S14C, Supporting Information). This comprehensive computational approach yields critical structural insights into the interaction between macrophage SIRPα and the tumor cell CD47 immune checkpoint, elucidating the role of αvβ3 in stabilizing CD47 distribution on the cell membrane (Figure 6B,D).

Given the crucial role of flexible loop regions in protein–protein interactions, we conducted truncation experiments using the CD47–HA‐tag constructs to delineate the regions within CD47 involved in binding to αvβ3. Specifically, our focus centered on loops within the CD47/αvβ3‐ECD interface, encompassing the K26–F32, T44–N50, T51–Y55, D69–N73, G62–T67, and D80–K85 loops (Figure 6C). Cotransfection of six HA–CD47 truncations with Flag–ITGAV or Myc–ITGB3 plasmids in HEK‐293 cells facilitated subsequent immunoprecipitation using Flag‐ and Myc‐beads, respectively. Remarkably, the HA–CD47^Δ51–55^ truncation exhibited a significant attenuation in its ability to directly bind to αvβ3 (Figure 6E,F). Consistent to impaired immunoprecipitation of αvβ3 by HA–CD47^Δ51–55^ truncation, the T51–Y55 loop is situated within the central region that participates in the CD47/αvβ3‐ECD interaction in the optimal model (Figure 6D).

To provide further supporting evidence, we have included bioluminescence resonance energy transfer (BRET) assays. BRET is an approach that detects molecular interactions by measuring the energy transfer between a luciferase donor and a fluorescent protein acceptor, which is widely used for protein–protein interaction studies.^[^ ^55^ ^]^ To directly observe the interaction between the CD47/αvβ3 complex, we engineered the luciferase donor Rluc8 at the C‐terminus of CD47 and the fluorescent protein eYFP at the C‐termini of ITG αv and ITG β3 (Figure 6G). As the relative ratios of eYFP and Rluc8 were increased upon transfection, we detected a significant BRET signal between the heterodimers consisting of CD47–Rluc8 and ITGαv–eYFP and ITGβ3–eYFP (^*^ *p* < 0.05, ^***^ *p* < 0.001, ^****^ *p* < 0.0001, *F* (10, 66) = 6.538, Two‐way ANOVA, compared to BRET signals without ITGαv–eYFP or ITGβ3–eYFP; Figure 6H), indicating that the physical distance between CD47 and αvβ3 heterodimers is less than 100 Å,^[^ ^55^ ^]^ facilitating the formation of a ternary complex. Importantly, coexpression of CD47 with either αv or β3 individually did not induce any BRET signal, highlighting the specificity of the interaction between CD47 and the ITGαv/ITGβ3 heterodimers (Figure 6H). These findings provide direct evidence for the interaction between CD47 and the ITGαv/ITGβ3 heterodimer, reinforcing previous observations that CD47 stabilizes the formation of ITGαv/ITGβ3 heterodimers on the cell membrane.

### 2.8 PSFL‐NK13, a Peptide Specifically Engineered to Target the CD47/αvβ3 Interaction, Showing Promise in Weakening the CD47–SIRPα Immune Checkpoint

**Figure 7:**
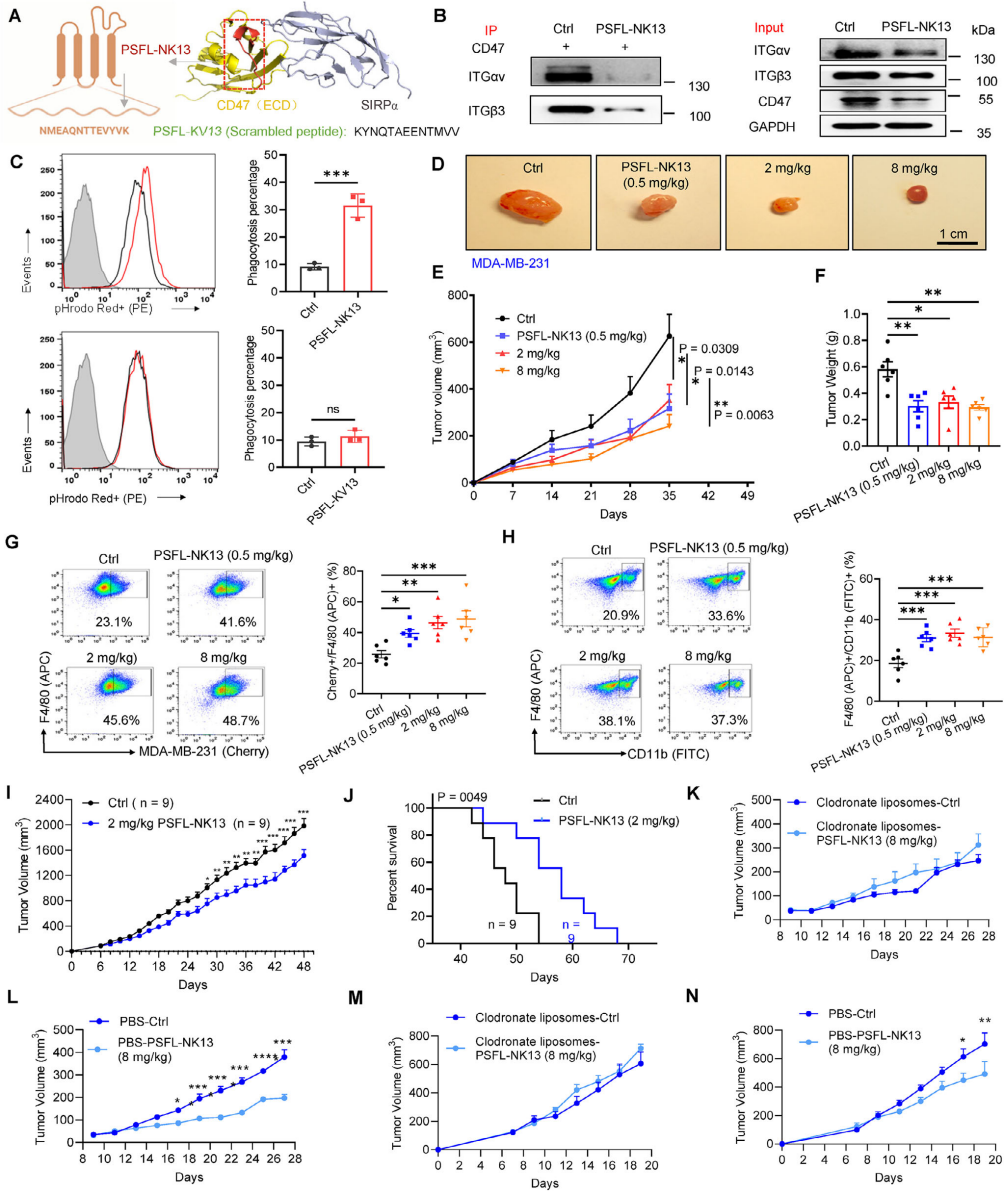
Suppression of tumor development using the specifically engineered inhibitory peptide PSFL‐NK13. A) Design of the interfering peptide based on the αvβ3–CD47 interaction. The position (highlighted in red) and sequence (in orange font) of the interfering peptide PSFL‐NK13M46‐K57. A control peptide, PSFL‐KV13 (in green font), was synthesized with amino acids randomly assigned based on the PSFL‐NK13 sequence. B) Peptide PSFL‐NK13 significantly reduced the interaction between CD47 and αvβ3 in transfected MDA‐MB‐231 cells (*n* = 3). C) Quantitative flow cytometry analysis of macrophage phagocytosis after 48 h incubation with peptide PSFL‐NK13 or PSFL‐KV13 (4 µm), with pooled data (right, *n* = 3). PSFL‐NK13, but not PSFL‐KV13, significantly increased macrophage phagocytosis of tumors. D–F) MDA‐MB‐231 shNC cells (2 × 10^6^) were implanted in the mammary fat pad of female nude mice, and tumor growth rates in mice treated with or without PSFL‐NK13 were compared. Images of four independent experimental cohorts are shown (D). Scale bar = 1 cm (D). Tumor volume (E) and weight (F) were measured after four weeks of PSFL‐NK13 treatment (0.5, 2, and 8 mg kg^−1^) (6 mice per group). G) Representative flow cytometry showing macrophage phagocytosis in Cherry+ MDA‐MB‐231 shNC tumors (6 mice per group) and quantitative assessment of macrophage infiltration by flow cytometry, i.e., MDA‐MB‐231(Cherry)+/F4/80(APC)+. H) Representative flow cytometry analysis of F4/80+/CD11b+ macrophages in peripheral blood mononuclear cells (PBMCs) and the ratio of macrophages assessed as F4/80(APC)+/CD11b(FITC)+ by flow cytometry quantification (*n* = 6). I,J) Following orthotopic implantation of MDA‐MB‐231 cells (4 × 10⁶) into the inguinal mammary fat pads of NSG mice, peritumoral subcutaneous administration of PSFL‐NK13 (2 mg kg^−1^) significantly delayed tumor progression (I) and prolonged survival (J). Data are expressed as mean ± s.e.m. (*n* = 9). Statistical significance was determined using two‐way ANOVA followed by Dunnett's multiple comparisons test: *p < 0.05, **p < 0.01, ***p < 0.001; (J) *F* (22, 365) = 3.33, *p* < 0.0001; survival log‐rank test: *p* = 0.0049. K–N) In NSG mice rendered macrophage‐deficient, PSFL‐NK13 failed to suppress tumor growth following MDA‐MB‐231 inoculation (K), whereas vehicle‐treated controls displayed robust tumor inhibition (L). Similarly, in Balb/C mice orthotopically engrafted with 4T1 cells (4 × 10⁶), macrophage depletion nullified PSFL‐NK13‐mediated tumor control (M), in contrast to pronounced suppression observed in PBS‐treated controls (N). PSFL‐NK13 was administered at 8 mg kg^−1^. All data are expressed as mean ± s.e.m. (*n* = 3–6); ^*^ *p* < 0.05, ^**^ *p* < 0.001, ^***^ *p* < 0.001 versus control, unpaired *t*‐test (C); one‐way ANOVA with Dunnett's post‐hoc test (E–N); (E), (*F* (3, 20) = 5.756), (F), (*F* (3, 20) = 9.91, *p* = 0.0003), (G), (*F* (3, 20) = 7.862, *p* = 0.0012), (H) (*F* (3, 20) = 11.90, *p* = 0.0001; (K) *F* (9, 82) = 0.9349, *p* = 0.4998; (L) *F* (9, 80) = 13.27, *p* < 0.0001; (M) *F* (7, 77) = 0.5828, *p* = 0.7679; (N) *F* (7, 69) = 2.391, *p* = 0.0299.

Truncation experiments pinpointed the T51–Y55 loop in CD47 as pivotal in this interaction. To preserve this critical region, we extended the sequence by 2 and 6 amino acids from both ends, resulting in the successful synthesis of the tridecapeptide PSFL‐NK13, with the sequence NMEAQNTTEVYVK (Figures 6D and 7A; Figure S15E, Supporting Information). As a comparative control, we synthesized PSFL‐KV‐13, featuring a randomly disrupted amino acid sequence relative to PSFL‐NK13 (**Figure** **7**A). Subsequent Co‐IP analysis on endogenously expressed proteins in MDA‐MB‐231 cells demonstrated PSFL‐NK13's efficacy in disrupting the binding of αvβ3 with CD47 (Figure 7B, left). Furthermore, PSFL‐NK13 induced a moderate reduction in the expression levels of both αvβ3 and CD47 (Figure 7B, right).

Furthermore, we utilized PSFL‐NK13 to investigate its capacity to disrupt the CD47–αvβ3 interaction within the ternary complex. HEK293T cells pretreated with 20 µm PSFL‐NK13 for 12 h post‐transfection exhibited an ≈20% reduction in BRET signal intensity (^****^ *p* < 0.0001 vs control, unpaired *t*‐test; Figure 6I). This result suggests that PSFL‐NK13 impairs the association between CD47 and the ITGαv–ITGβ3 heterodimer, thereby diminishing the stability of the ternary complex.

Incubation of MDA‐MB‐231 cells with increasing concentrations of PSFL‐NK13 (0, 0.5, 1, 2, and 4 µm) resulted in a concentration‐dependent decrease in the surface expression of αvβ3 and CD47 (*p* < 0.05, 0.01, or 0.001, vs control, one‐way ANOVA with Bonferroni post‐hoc test; Figure S16A, Supporting Information). By contrast, 4 µm of the control peptide PSFL‐KV‐13 showed no significant effect on αvβ3 and CD47 expression (*p* > 0.05 vs control, Student's *t*‐test; Figure S16B, Supporting Information). These findings underscore the potential of PSFL‐NK13 to disrupt the interaction between αvβ3 and CD47, likely through specific interactions with the interface between these proteins.

We then investigated the potential of PSFL‐NK13 to modulate macrophage‐mediated phagocytosis of tumor cells. MDA‐MB‐231 cells labeled with pH‐sensitive dye probes were cocultured with macrophages, revealing a nearly twofold increase in average fluorescence intensity in the peptide‐treated group compared to controls postphagocytosis, indicative of enhanced tumor cell phagocytosis by macrophages (*p* < 0.01 vs control, Student's *t*‐test; Figure S16C–F, Supporting Information). Flow cytometry analysis further confirmed a significant, nearly threefold increase in tumor cell phagocytosis by macrophages upon PSFL‐NK13 treatment (control vs PSFL‐NK13, *p* = 0.0010, Student's *t*‐test; Figure 7C), which was not observed with the control peptide PSFL‐KV‐13 (*p* = 0.2981; Figure 7C).

The effect of PSFL‐NK13 on αvβ3 activation was also evaluated. When FN at a concentration of 20 ng mL^−1^ was applied to MDA‐MB‐231 cells, intracellular Ca^2+^ levels were elevated (control vs FN, *p* = 0.0013, Student's *t*‐test; Figure S17A, Supporting Information), indicating that αvβ3 was activated. Furthermore, the αvβ3 peptide inhibitor cyclo(─RGDfK), but not PSFL‐NK13, inhibited the FN‐induced increase in αvβ3 surface expression in cancer cells (FN vs FN + cyclo(─RGDfK), *p* = 0.0084; FN vs FN + PSFL‐NK13, *p* = 0.6191, one‐way ANOVA with Bonferroni post‐hoc test; Figure S17B, Supporting Information). Similarly, cyclo(─RGDfK) inhibited the Ca^2+^ elevation during αvβ3 activation, whereas PSFL‐NK13 did not (FN vs FN + cyclo(─RGDfK), *p* = 0.0027; FN vs FN + PSFL‐NK13, *p* = 0.6186, one‐way ANOVA with Bonferroni post‐hoc test; Figure S17C, Supporting Information). These results suggest that PSFL‐NK13 exerts its effects not through direct αvβ3 activation but rather by targeting the interface between αvβ3‐ECD and CD47‐ECD (Figure 6B), consistent with our hypothesis (Figure 6B,D).

### 2.9 PSFL‐NK13 Markedly Inhibits Tumor Progression across Diverse Mouse Models

To further assess its antitumor efficacy, in vivo evaluations were performed. Two weeks postinjection of MDA‐MB‐231 cells into the mammary fat pads of nude mice, PSFL‐NK13 was administered via subcutaneous topical application at doses of 0.5, 2, and 8 mg kg^−1^, 3 times a week. After four weeks of treatment, tumor volume and weight were compared. Notably, the control group exhibited larger tumors, with significantly higher tumor weights compared to all treatment groups (*p* < 0.05 or 0.01, one‐way ANOVA with Bonferroni post‐hoc test; Figure 7D,E).

Furthermore, we observed increased infiltration and phagocytosis of tumors by macrophages in the PSFL‐NK13 administered groups across all three doses compared to the control group (Cherry+/F4/80+: *p* = 0.0012, Cherry+/CD11b: *p* = 0.003, vs control, one‐way ANOVA with Bonferroni post‐hoc test; Figure 7G; Figure S18A, Supporting Information). There was also an increased F4/80+/CD11b+ expression of macrophages in the peripheral blood (*p* = 0.0001; Figure 7H). These findings underscore the ability of PSFL‐NK13 to enhance macrophage‐mediated phagocytosis of tumor cells, thereby inhibiting tumor growth.

We next established a subcutaneous xenograft model by inoculating MDA‐MB‐231 cells (4 × 10⁶) into the flank of NSG mice (NOD.Cg‐PrkdcscidIl2rgem1Smoc, NM‐NSG‐001). Once tumors reached ≈200 mm^3^, mice were randomized into treatment groups and administered 2 mg kg^−1^ PSFL‐NK13 (Figure 7I) via peritumoral subcutaneous injection every other day. Ethical endpoints were defined as tumor diameter exceeding 20 mm or tumor volume surpassing 2000 mm^3^, with mortality observed starting around day 40 postinoculation. Both 2 mg kg^−1^ PSFL‐NK13 significantly prolonged survival in tumor‐bearing mice (Kaplan–Meier analysis: *p* = 0.0049; Figure 7J). Notably, tumor volume in the 2 mg kg^−1^ PSFL‐NK13 group began to diverge significantly from the control group as early as day 28 (phosphate‐buffered saline (PBS)‐Ctrl = 910 ± 113 mm^3^ vs PSFL‐NK13 = 754 ± 84 mm^3^) and remained statistically distinct by day 48 (PBS‐Ctrl = 1984 ± 119 mm^3^ vs PSFL‐NK13 = 1385 ± 154 mm^3^; ^*^ *p* < 0.05, ^**^ *p* < 0.01, ^***^ *p* < 0.001, two‐way ANOVA with Dunnett's post‐hoc test; (B), *F* (22, 356) = 5.12, *p* < 0.0001).

To verify that the observed attenuation in tumor growth following PSFL‐NK13 treatment was indeed mediated by macrophage‐driven phagocytosis. NSG mice were inoculated with 4 × 10⁶ MDA‐MB‐231 cells subcutaneously into the left flank, followed by administration of the respective treatments. No significant differences in tumor growth were observed between the macrophage depletion agent‐Ctrl group and the macrophage depletion agent‐8 mg kg^−1^ NK13 group (Figure 7K). Conversely, the PBS (lipid nanoparticle control)‐8 mg kg^−1^ PSFL‐NK13 group exhibited a notable deceleration in tumor growth when compared to the PBS‐Ctrl group (two‐way ANOVA with Dunnett's post‐hoc test, *F* (9, 80) = 13.27, ^*^ *p* < 0.05, ^***^ *p* < 0.001, Figure 7L). Statistically significant differences were detected as early as day 17 (PBS‐Ctrl = 122 ± 23 mm^3^, PSFL‐NK13 = 86.3 ± 5.2 mm^3^), and persisted through day 27 (PBS‐Ctrl = 320 ± 65 mm^3^, PBS‐NK13 = 197 ± 16 mm^3^).

To further substantiate the role of macrophages in mediating the antitumor effects of PSFL‐NK13, we employed a murine syngeneic tumor model in which 4 × 10⁶ 4T1 mammary carcinoma cells were subcutaneously inoculated into the left flanks of BALB/c mice. Following randomization, animals were subjected to macrophage depletion or vehicle control, in conjunction with 8 mg kg^−1^ PSFL‐NK13 treatment, and tumor burden was monitored longitudinally. No statistically significant differences in tumor progression were observed between macrophage‐depleted control and macrophage‐depleted PSFL‐NK13–treated mice (Figure 7M). By contrast, in the context of intact macrophage populations, PSFL‐NK13 treatment led to a pronounced suppression of tumor growth relative to PBS control (*F* (7, 69) = 2.391, *p* = 0.0299; ^*^ *p* < 0.05, ^**^ *p* < 0.01; Figure 7N), with significant tumor volume divergence noted on day 17 (PBS‐Ctrl: 613 ± 132 mm^3^ vs PBS‐PSFL‐NK13: 448 ± 112 mm^3^) and sustained through day 19 (702 ± 156 mm^3^ vs 491 ± 174 mm^3^). Collectively, results from both the MDA‐MB‐231 xenograft model in NSG mice and the syngeneic 4T1 model in immunocompetent BALB/c mice provide compelling evidence that the antitumor activity of PSFL‐NK13 is critically dependent on macrophage functionality.

In order to evaluate the generalizability of PSFL‐NK13‐mediated modulation of the CD47/αvβ3 axis, we examined its efficacy in HCT‐116 colorectal carcinoma and A549 non‐small‐cell lung cancer cell lines. Treatment with 4 µm PSFL‐NK13 resulted in statistically significant reductions in the surface expression levels of αvβ3 and CD47 (*p* < 0.05 or 0.01, Student's *t*‐test; Figure S20A,B, Supporting Information). When benchmarked against MDA‐MB‐231 cells—where an ≈26% and ≈19% decrease in αvβ3 and CD47 expression, respectively, was observed (Figure S16A, Supporting Information), along with an ≈40% increase in macrophage‐mediated phagocytosis (Figure S16C–F, Supporting Information)—the suppressive effect of PSFL‐NK13 in HCT‐116 and A549 cells appeared less robust, accompanied by relatively attenuated enhancement of phagocytic activity. These data suggest that the molecular coupling and costabilization of CD47 and αvβ3 at the tumor cell surface may be context‐dependent, varying across tumor types due to distinct regulatory architectures.

Following subcutaneous transplantation of HCT116 in nude mice, treatment with local subcutaneous injections of PSFL‐NK13 at a dose of 2 mg kg^−1^ per day significantly delayed the increase in tumor growth volume one week posttreatment (control vs 2 mg kg^−1^ PSFL‐NK13, *p* = 0.0063, Student's *t*‐test; Figure S19A,B, Supporting Information). Moreover, a significant reduction in tumor weight was observed after three weeks compared to the control group (*p* = 0.0002, Student's *t*‐test, measured at day 21, Figure S19C, Supporting Information). Notably, PSFL‐NK13 treatment also promoted macrophage infiltration of HCT116 tumor tissue and enhanced macrophage phagocytosis, as indicated by increased CD11b expression (control vs 2 mg kg^−1^ PSFL‐NK13, *p* = 0.0197, Student's *t*‐test; Figure S18B, Supporting Information), alongside an increase in macrophage infiltration in peripheral blood (31.2 ± 9.0% vs 45.0 ± 10.6%, *p* = 0.0347; Figure S18C, Supporting Information). These findings collectively suggest that while PSFL‐NK13 may exert relatively weaker effects on tumor phagocytosis by macrophages at the cellular level compared to its action on TNBC, it remains effective against other subcutaneously grafted tumors.

We also employed a breast cancer cell model 4T1 in the mammary fat pads of immunocompetent BALB/c female mice. After establishing in situ tumor models using the 4T1 breast cancer cell line in the mammary fat pads of immunocompetent BALB/c female mice, mice were evenly grouped based on tumor size one‐week posttransplantation. Following two weeks of subcutaneous topical administration of PSFL‐NK13 at a dosage of 2 mg kg^−1^ per day, treated mice exhibited a significant reduction in tumor volume compared to controls (*p* = 0.0042 vs control, Student's *t*‐test, see Figure S19D,E in the Supporting Information). Similarly, a significant difference in tumor weight was evident (control vs 2 mg kg^−1^ PSFL‐NK13, *p* = 0.0005, Student's *t*‐test; Figure S19D,F, Supporting Information), accompanied by enhanced macrophage infiltration in tumor tissue (*p* = 0.0098; Figure S18D, Supporting Information) and an increased proportion of macrophages in peripheral blood (*p* = 0.0273; Figure S18E, Supporting Information). These results further support the efficacy of PSFL‐NK13 in tumor suppression, extending its effectiveness to immunocompetent mouse models.

### 2.10 PSFL‐NK13 Demonstrates No Hemolytic Effects Associated with CD47 Antibodies, nor Does It Promote Tumor Angiogenesis Caused by Low Concentrations of αvβ3 Inhibitors

**Figure 8:**
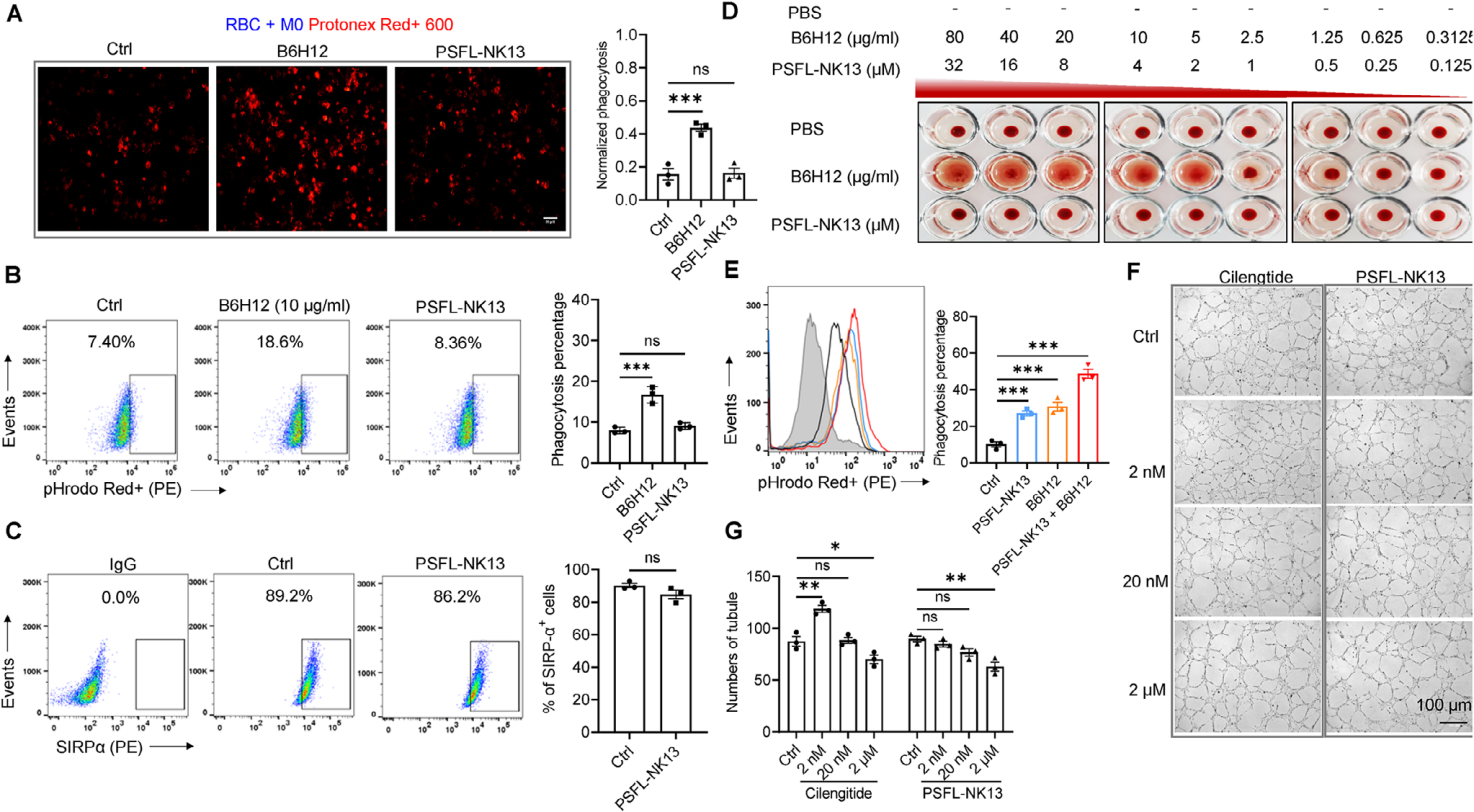
PSFL‐NK13 demonstrates minimal side effects comparable to CD47 antibodies and αvβ3 Inhibitors. A) Representative fluorescence microscopy images depicting macrophages (Protonex Red+, red) phagocytosing erythrocytes in vitro after 36 h of coculture with PSFL‐NK13 peptide (4 µm) and CD47 antibody (B6H12, 10 µg mL^−1^), accompanied by quantitative analysis of mean fluorescence intensity in confocal images. Scale bar = 50 µm. B) Representative flow cytometry plots illustrating erythrocyte phagocytosis following treatment with PSFL‐NK13 peptide (4 µm) or CD47 antibody (B6H12, 10 µg mL^−1^), alongside summarized data (right panel). C) Flow cytometry analysis of SIRPα surface expression on M0 macrophages treated with PSFL‐NK13 peptide (4 µm) for 48 h, with pooled data. D) Erythrocyte suspensions seeded in 96‐well circular (U) bottom plates and incubated with CD47 antibody (B6H12) and PSFL‐NK13 peptide. Antibodies were diluted in two gradients from 80 to 0.3125 µg mL^−1^, and peptides were diluted in two gradients from 32 to 0.125 µm. E) Representative flow cytometry histograms depicting RBC phagocytosis after coincubation with PSFL‐NK13 peptide (4 µm) or CD47 antibody (B6H12, 10 µg mL^−1^), with pooled data (right). F,G) HUVEC plated on matrigel‐coated 48‐well plates in the presence of 50 ng mL^−1^ VEGF and treated with various concentrations of PSFL‐NK13 peptide or cilengitide (2, 20 nm, and 2 µm) for 6 h. Representative images are displayed in (F). The formed capillary‐like tube structures were observed under an inverted microscope, and the number of tubes per image was calculated using the Angiogenesis Analyzer plugin for ImageJ (G). All data are expressed as mean ± s.e.m., *n* = 3 independent experiments; ^*^ *p* < 0.05, ^**^ *p* < 0.001, ^***^ *p* < 0.001 versus control, unpaired *t*‐test (C); one‐way ANOVA with Dunnett's post‐hoc test (A, B, E, G); (A), (*F* (2, 6) = 29.47, *p* = 0.0008), (B), (*F* (2, 6) = 38.50, *p* = 0.0004), (E), (*F* (3, 8) = 61.05, *p* < 0.0001), (G), (*F* (3, 8) = 28.37, *p* = 0.0001), (*F* (3, 8) = 11.86, *p* = 0.0026). ns, not significant.

To assess potential side effects of PSFL‐NK13, we investigated its impact on erythrocyte phagocytosis by macrophages.^[^ ^11^, ^12^ ^]^ Despite its targeting of the CD47/αvβ3 interface on cancer cell membranes, PSFL‐NK13 did not alter CD47 protein expression on human erythrocytes (control vs 4 µm PSFL‐NK13, *p* = 0.6336, Student's *t*‐test; Figure S21A, Supporting Information), nor did it enhance erythrocyte phagocytosis by M0 macrophages, in contrast to the effect observed with the CD47 antibody B6H12 (ratio of erythrocytes labeled by red pH‐sensitive dye protonex Red 600: *p* = 0.9798 vs control, **Figure** **8**A). This lack of enhancement was confirmed by flow cytometry quantification (PSFL‐NK13 (4 µm, *p* = 0.4184) and B6H12 (*p* = 0.0004), respectively; one‐way ANOVA with Bonferroni post‐hoc test; Figure 8B).

Additionally, coculturing MDA‐MB‐231 cells with Mφ macrophages derived from human PBMC revealed that PSFL‐NK13 facilitated the phagocytosis of cancer cells by macrophages, similar to the effect observed with B6H12 (*p* = 0.0016, one‐way ANOVA with Bonferroni post‐hoc test; Figure S21B, Supporting Information). Furthermore, PSFL‐NK13 exhibited no direct toxicity on MDA‐MB‐231 cells, as evidenced by consistent cell viability across concentrations (one‐way ANOVA with Bonferroni post‐hoc test, *p* = 0.3883; Figure S21C, Supporting Information). Importantly, PSFL‐NK13 did not affect the expression of SIRPα protein on macrophage membranes (control vs 4 µm PSFL‐NK13, *p* = 0.3992; Figure 8C), reinforcing its specificity for CD47/αvβ3 interactions on cancer cell membranes without impacting SIRPα on macrophage membranes.

The hemolytic effects of CD47 antibody B6H12 were examined across a concentration range of 0.3125–80 µg mL^−1^, with concentrations over 5 µg mL^−1^ resulting in observable hemolysis (Figure 8D). Conversely, PSFL‐NK13, tested at concentrations of 0.125–32 µm, did not induce hemolysis, even at higher concentrations such as 100 and 200 µm (Figure S21D, Supporting Information). Additionally, antibodies targeting αv, β3, and αvβ3, namely Arg108, Ile114, and 23C6, respectively, demonstrated no hemolytic effects on human erythrocytes (Figure S21E, Supporting Information). These findings suggest that CD47 primarily serves to prevent phagocytosis by macrophages^[^ ^12^ ^]^ and that αvβ3 is not directly involved in this process. PSFL‐NK13, while lacking hemolytic properties, disrupts CD47/αvβ3 interactions on cancer cell membranes without affecting erythrocytes. Additionally, when combined with CD47 antibody B6H12, 4 µm PSFL‐NK13 enhanced macrophage phagocytosis of tumor cells in MDA‐MB‐231 and M0 coculture system, compared to B6H12 alone (*p* < 0.0001 vs control, one‐way ANOVA with Bonferroni post‐hoc test; Figure 8E), making PSFL‐NK13 a promising candidate for therapeutic use.

Finally, considering the paradoxical effects of αvβ3 inhibitors like cilengitide on tumor angiogenesis (e.g., 2 nm),^[^ ^56^ ^]^ it is crucial to assess potential side effects. We investigated whether PSFL‐NK13 affects angiogenesis in human umbilical vein endothelial cells (HUVEC) cells, known to express high levels of αvβ3 upon activation.^[^ ^57^ ^]^ Remarkably, PSFL‐NK13 did not significantly alter αvβ3 or CD47 expression in HUVEC cells (*p* > 0.05 vs control, Student's *t*‐test; Figure S21F,G, Supporting Information), nor did it affect intracellular Ca^2+^ concentration in HUVEC cells (control vs 4 µm PSFL‐NK13, as measured by the Fluo‐4 AM probe, *p* > 0.05, Student's *t*‐test; Figure S21H, Supporting Information). By contrast, cilengitide exhibited dual effects on tubule formation by endothelial cells, promoting at low concentrations (e.g., 2 nm) but inhibiting at higher concentrations (the number of tubules: 87 ± 8, 118 ± 5, 88 ± 5, and 70 ± 7 for control, 2, 20 nm and 2 µm cilengitide, respectively; *p* < 0.01 or 0.001, one‐way ANOVA with Bonferroni post‐hoc test; Figure 8F,G). Conversely, PSFL‐NK13 did not promote tubule formation at any tested concentration (2, 20 nm and 2 µm), with an inhibitory effect being observed at higher concentrations (89.7 ± 4.5, 84.7 ± 4.7, 76.7 ± 6.1, and 63.0 ± 7.5 for control, 2, 20 nm and 2 µm, respectively; *p* > 0.05 or *p* = 0.0026 for 2 µm; Figure 8F,G, Supporting Information), indicating its efficacy in enhancing macrophage phagocytosis of tumor cells while avoiding hemolytic side effects and maintaining endothelial cell function. This dual action enhances both safety and efficacy, making PSFL‐NK13 a promising candidate for therapeutic use.

## 3 Discussion

Here, we introduce a novel mechanism wherein integrin αvβ3 and the CD47–SIRPα axis stabilize each other on tumor cell surfaces, enhancing tumor immune evasion. Our analysis of protein expression in clinical tumor tissues reveals a significantly higher proportion of integrin αvβ3 and CD47 double‐positive cells compared to normal tissues (Figure 1; Figure S2, Supporting Information). Through siRNA and shRNA experiments, we demonstrate the mutual stabilization of αvβ3 and CD47 on cancer cell membranes. However, as αvβ3 is absent on human erythrocyte membranes, this costabilization represents only one aspect of tumor cells' evasion of macrophage‐mediated phagocytosis (Figures 1, 2, 3, 4; Figures S3–S10, Supporting Information). Supporting this notion, our investigations show that the αvβ3/CD47 interfering peptide PSFL‐NK13 disrupts the αvβ3/CD47–SIRPα axis without inducing the hemolytic side effects associated with CD47 antibodies (Figures 6, 7, 8), presenting a promising avenue for safe and effective cancer immunotherapy. Notably, this therapeutic strategy diverges from previous peptide‐based efforts targeting the CD47–SIRPα axis,^[^ ^58^, ^59^, ^60^, ^61^ ^]^ representing a novel avenue for the development of safe and effective immunotherapies.

Although αvβ3 on tumor cell membranes can switch between activated and inactivated states,^[^ ^15^ ^]^ the mechanism by which αvβ3 interacts with CD47 and stabilizes the CD47–SIRPα axis appears to rely more on its bent closed conformation or extended closed intermediate conformers (Figure S11, Supporting Information). Several lines of evidence support this idea. First, the CD47‐ECD belongs to the IgV domain,^[^ ^39^ ^]^ whose 3D structure consists of multiple rigid β‐sheets (Figure S14A, Supporting Information), except for the connecting regions or loops between the β‐sheets. Furthermore, the CD47‐ECD is ≈40 Å away from the cell membrane and can only reach the EGF4 and βTD regions of the β3 subunit and the Calf‐2 of the αv subunit (Figure 6B; Figure S14A, Supporting Information). Therefore, the interaction between CD47 and αvβ3 may occur through an “induced fitting” mechanism, adjusting the ECD/IgV spatial orientation to allow linking regions or loops between the β‐sheets to interact with regions of αvβ3 close to the membrane (Figure 6D; Figure S14A,C, Supporting Information). Moreover, the EGF4 and βTD regions of ITG‐β3, as well as the Calf‐2 of αv, can accommodate CD47‐ECD only when αvβ3 is in a bent closed or extended closed intermediate conformer (Figure S11, Supporting Information), as these regions are much more distant in the activated state and can no longer interact with CD47 (Figure S11B, Supporting Information). Second, while factors influencing αvβ3 activation, such as alterations in calcium concentration,^[^ ^49^ ^]^ the presence of the activating ligand FN and the key intracellular proteins Talin and Kindlin,^[^ ^62^ ^]^ affect the stable coexpression of αvβ3 and CD47 on the membrane of cancer cells, their effect is only one‐third of that of the effect of αv‐siRNA knockdown (Figure 5; Figure S12, Supporting Information). Thus, other nonactivated αvβ3 conformers are more frequently involved (Figure S11, Supporting Information). Third, the FN activation of αvβ3 was found to enhance the coexistence of CD47 with αvβ3 (Figure 5E), whereas knockdown of Talin‐1 and Kindlin‐2 (Figure 5G–I), both of which are key proteins for integrin activation, had an attenuated or unchanged effect, respectively. These findings imply that specific conformations or states of αvβ3 play a pivotal role in determining its interaction with CD47, surpassing mere dependence on its activation state. Fourth, both activating and inactivating antibodies to αvβ3 could affect the coexistence state of αvβ3 and CD47 on cancer cell membranes (Figures 4 and 5). Moreover, although the RGDS peptide can inhibit αvβ3 activation, it does not affect the αvβ3/CD47 coexistence state (Figure 5B; Figure S12A,B, Supporting Information). This suggests that characterizing the conformer of αvβ3 interacting with CD47 cannot be exclusively based on the activated or inactivated states alone. Finally, although PSFL‐NK13 significantly altered the coexistence of αvβ3 and CD47 on cancer cell membranes, it did not affect FN‐induced activation of αvβ3 (Figure S17B,C, Supporting Information). By contrast, the αvβ3 inhibitor cilengitide does affect this activation (Figure S17B,C, Supporting Information). Thus, activation of αvβ3 is not essential for its coexistence with CD47 on the cancer cell membrane.

The coexistence of αvβ3 and CD47 strengthens the CD47–SIRPα axis and presents a novel mechanism for immune checkpoint therapy using the interfering peptide PSFL‐NK13. This approach may offer advantages over antibodies, inhibitors, or siRNA targeting CD47, αvβ3, and SIRPα. Effective CD47 antibodies must possess both target‐blocking activity and Fc effector function;^[^ ^2^ ^]^ retaining only the former reduces efficacy. Additionally, some CD47 antibodies are limited to hematological tumors and require combination with other antibodies.^[^ ^31^, ^63^ ^]^ IgG4 antibodies against CD47 are ineffective against solid tumors, possibly due to CD47's expression on erythrocytes, which depletes the antibody before it can reach tumor cells. Integrin αvβ3 is highly expressed in activated endothelial cells, neovasculature, and various tumor cells.^[^ ^1^, ^12^ ^]^ Although antibodies or inhibitors targeting αvβ3 can inhibit tumor angiogenesis, they have shown limited efficacy in clinical trials.^[^ ^14^ ^]^ Moreover, certain inhibitors may promote tumor angiogenesis at low concentrations.^[^ ^64^ ^]^ Similarly, while SIRPα antibodies do not affect erythrocytes or cause hemolysis, their Fc effects can induce cytotoxicity in various myeloid cells, including neutrophils and monocytes.^[^ ^65^ ^]^ By contrast, PSFL‐NK13 disrupts the CD47–SIRPα axis by interfering with the coexistence of αvβ3 and CD47, specifically enhancing macrophage‐mediated phagocytosis of tumor cells without affecting erythrocyte function (Figure 8), making it a safer alternative to the aforementioned strategies. Furthermore, PSFL‐NK13 demonstrated efficacy comparable to CD47 antibodies in enhancing phagocytosis (Figures 7 and 8). Future applications could include combining PSFL‐NK13 with other antibodies, as it has shown synergy with the CD47 antibody B6H12.

The potential impact of PSFL‐NK13 on the coexistence of CD47 and integrins under normal conditions must be thoroughly considered. In some normal tissues or during mild inflammation, CD47 may bind to integrin αvβ3 via its IgV domain, thereby activating integrins and initiating subsequent intracellular signaling.^[^ ^34^, ^36^, ^39^ ^]^ This activation could enhance the binding of integrins to various ligands such as fibronectin and fibrinogen.^[^ ^32^, ^66^, ^67^ ^]^ The link between integrin αvβ3 and CD47 has been demonstrated in both mouse models of osteoarthritis and patients with osteoarthritis,^[^ ^36^ ^]^ where synergistic signaling by CD47 and αvβ3 resulted in enhanced activation and inflammatory responses in synoviocytes and monocyte macrophages. These findings suggest that CD47 and integrin αvβ3 are involved in both physiological and inflammatory responses.^[^ ^36^ ^]^ Therefore, the potential effects of PSFL‐NK13 intervention on CD47 and integrins in normal tissues warrant discussion. Two points suggest that PSFL‐NK13 might exhibit greater specificity and safety in cancer therapy compared to its impact on normal tissues. First, while prior research focused on the influence of CD47 on αvβ3 activation,^[^ ^34^, ^36^, ^39^ ^]^ our findings indicate that αvβ3 and CD47 on the membrane surface of cancer cells predominantly coexist in an inactivated conformer. This suggests that PSFL‐NK13 exerts minimal impact on tissues other than tumors. Consistent with this, our data show no significant influence of PSFL‐NK13 on FN‐induced elevation of intracellular Ca^2+^ concentration, no erythrocyte lysis side effects, and a lack of protumor angiogenic side effects (Figure 8; Figures S16–S21, Supporting Information). Second, clinical investigations suggest that antitumor approaches targeting αvβ3 or CD47 exhibit a notable safety profile, with exceptions such as hemolysis and modified platelet activity associated with CD47 antibodies and protumor angiogenesis observed at low doses of αvβ3 inhibitors.^[^ ^68^, ^69^ ^]^ This suggests that PSFL‐NK13 is unlikely to induce additional toxicity, even when interacting with the αvβ3–CD47 complex in noncancerous cells.

Additionally, although disrupting αvβ3/CD47 interactions on the membranes of different cancer cells can enhance macrophage phagocytosis of tumors, but the effectiveness varies (Table S1, Supporting Information). This variability may be due to differences in the abundance and types of αv‐family integrins expressed on the membranes of different cancer cells, as well as their likelihood of coexisting with CD47 and the strength of their binding interactions. Additionally, other key proteins associated with the CD47–SIRPα axis, such as SHP‐1, SHP‐2, TSP‐1, other integrin proteins (e.g., αIIbβ3, α2β1, α4β1, and α6β1), other membrane proteins (e.g., VEGFR2, CD36, and Fas), and intracellular ligands (e.g., Gi‐proteins, BNIP3, Src, and MEK), exhibit differences across various types of cancer cells.^[^ ^70^ ^]^ One study confirmed the tumor‐killing effect of CD47 antibody by activating CD8+ T cells and dendritic cells.^[^ ^71^ ^]^ The phagocytosis of tumor cells and the subsequent processing of specific antigens for presentation to CD8+ T cells activate the tumor‐specific adaptive immune system. Although αvβ3 and CD47 coexist in most tumor cells and reinforce immune escape from macrophages, the interactions between macrophages and T cells may differ among cancer types.

Finally, our study extensively examines the functional interplay between CD47/αvβ3 heteromeric complexes at the tumor cell membrane and the CD47–SIRPα immune checkpoint. Pharmacological intervention with cyclo(─RGDfK) or Cilengitide results in a pronounced reduction in membrane‐localized CD47/αvβ3, paralleled by a decline in total protein abundance. The mechanistic basis for these observations is multifactorial. First, the maintenance of adequate membrane expression of transmembrane proteins necessitates a threshold level of total protein synthesis to ensure proper trafficking to the cell surface. Second, the CD47/αvβ3 complex is stabilized through specific conformational arrangements, which may be modulated at transcriptional, translational, or posttranslational levels. Cyclo(─RGDfK) and Cilengitide are likely to perturb the costable conformational state of CD47/αvβ3, triggering alterations in downstream intracellular signaling pathways that compromise membrane stability and promote endocytosis or lysosomal degradation. These findings align with the modulatory influence of established αvβ3 integrin regulators—such as fibronectin, talin‐1, and extracellular calcium ions—on CD47 membrane localization (Figure 5E–I; Figure S12, Supporting Information). Furthermore, the membrane‐resident CD47/αvβ3 complex is mechanistically coupled to the CD47–SIRPα immune checkpoint. Analogous to G‐protein‐coupled receptors and ion channel receptors, small variations in membrane distribution (on the order of 5–20%) can substantially alter receptor‐mediated physiological and pathological processes.^[^ ^72^, ^73^ ^]^ Consequently, a 10–20% alteration in the surface distribution of CD47/αvβ3 is predicted to modulate CD47–SIRPα checkpoint signaling in a manner consistent with fundamental principles governing membrane protein trafficking and surface stability.^[^ ^72^, ^73^ ^]^

In conclusion, this study delineates a mechanistic framework in which integrin αvβ3 and the CD47–SIRPα immune checkpoint engage in reciprocal stabilization at the plasma membrane of tumor cells, thereby potentiating immune escape. We further demonstrate that peptide‐based perturbation of this spatial organization significantly impairs checkpoint functionality and augments macrophage‐driven tumor cell clearance, with disproportionately strong efficacy in triple‐negative breast cancer.

## 4 Experimental Section

### Chemicals and Peptides

Cilengitide (HY‐16141), cyclo(─RGDfK) (HY‐P0023), RGDS (HY‐12290), and CWHM‐12 (HY‐18644) were purchased from MedChemExpress. Peptides PSFL‐NK13 and PSFLKV13 were synthesized by GL Biochemistry Co. Ltd. FN (8248) was purchased from ScienCell. EGTA (324626) and other inorganic salts and reagents were obtained from Sigma‐Aldrich.

### Cell Cultures

THP‐1, MCF‐7, HCT116, NCI‐H23, HL‐60, and A549 cells were cultured in RPMI‐1640 medium (C22400500BT, Gibco) supplemented with 10% fetal bovine serum (FBS, 10099‐141C, Gibco), 1% penicillin/streptomycin (C0222, Beyotime). MDA‐MB‐231 cells were cultured in L‐15 Medium (Leibovitz, L620KJ, BasalMedia) supplemented with 10% FBS, 1% penicillin/streptomycin. MDA‐MB‐453, 4T1, U87, and HEK293 cells were cultured in Dulbecco's modified Eagle medium (C11965500BT, Gibco) supplemented with 10% FBS, 1% penicillin/streptomycin. LoVo cells were cultured in F‐12K nutrient mixture (21127‐022, Gibco) supplemented with 10% FBS, 1% penicillin/streptomycin. HUVEC cells were cultured in ECM medium (1001, ScienCell) supplemented with 10% FBS, 1% endothelial cell growth supplement (1052, ScienCell), 1% penicillin/streptomycin. All cells were cultured in a humidified incubator containing 5% CO_2_ at 37 °C.

### Multiplexed Immunofluorescence of Tissue Samples from Breast Cancer Patients

Tissue microarray slides (Shanghai Otto Biotechnology Co., Ltd.) contained 159 formalin‐fixed, paraffin‐embedded samples from breast cancer patients and some normal tissue samples. Eight of the samples had normal or paraneoplastic tissue detached during processing, and the final data consisted of 119 breast cancer tissue samples and 32 normal/paraneoplastic tissue samples.^[^ ^74^ ^]^ All breast cancer tissue samples were collected on the day of surgery and prior informed consent was obtained, and the study was approved by the Human Research Ethics Committee of Zhejiang Taizhou Hospital.

Multiplexed tyramide signal amplification (TSA) immunofluorescence staining was performed using the Opal 7‐color Manual IHC Kit (NEL801001KT, PerkinElmer).^[^ ^75^ ^]^ Dewaxing and heat‐induced epitope retrieval was performed on slides at 100 °C for 20 min. Antibody application, detection, and TSA amplification were performed in four consecutive rounds according to the general procedure: the first round of staining was performed using mouse antihuman integrin αvβ3 antibody (520 nm, green, ab190147, Abcam) at a dose of 1/2000; the second round used mouse antihuman CD47 antibody (570 nm, red, 323102, Biolegend) at a dose of 1/100; and the third round used mouse antihuman CD68 antibody (650 nm, sky blue, PA014, Abcarta) at a ready‐to‐use price. The fourth round used Rabbit antihuman Pan‐CK antibody (690 nm, grayish purple, ab9377, Abcam) at a dose of 1/20. After labeling with TSA, a hot stripping step at 100 °C was used in the staining round and the antibody was removed after 10 min. Finally, the nuclei were counterstained with 0.33 µg mL^−1^ of DAPI (blue) for 15 min. The slides were scanned using the TissueFAXS Spectra (Meyer) multispectral imaging system (TissueGnostics StrataQuest7.0) and analyzed at the single‐cell level using StrataQuest analysis software. The StrataQuest analysis software allowed obtaining grayscale maps of the different channels. Individual cells could be identified and circled by applying a nucleus recognition algorithm to the DAPI channel image. The details were as follows: after the nuclei were identified according to DAPI, the valid nuclei were screened according to the fluorescence intensity and mapping area, i.e., the number of cells counted in the statistics. And αvβ3 and CD47 were fluorescently identified, i.e., αvβ3 (green)+, CD47 (red)+, and DAPI (blue), and then the fluorescence intensities of αvβ3 and CD47 were used as the horizontal and vertical coordinates to divide the double‐positive cells, respectively.

### RT‐qPCR

Total RNA was isolated and reverse transcribed using EZ‐press RNA purification kit (B0004‐plus, EZBioscience) and PrimeScript RT kit (RR047A, TaKaRa), respectively, according to the manufacturer's instructions.^[^ ^76^ ^]^ qPCR was performed using TB Green Premix Ex Taq (RR420A, TaKaRa) for qPCR; relative changes in gene expression were determined using the 2^−ΔΔCt^ method, and relative mRNA expression was normalized to GAPDH. The sequences of the primers are listed in Table S3 (Supporting Information).

### Western Blotting

Tumor cells were lysed in lysis buffer (P0013, Beyotime) containing 1% cocktail (B14001, Bimake).^[^ ^77^ ^]^ Cell lysates were cleared by centrifugation at 12 000 *g* for 15 min at 4 °C. The concentration of total protein was determined using the Enhanced BCA Protein Assay Kit (P0010, Beyotime). Protein sample buffer was added and the samples were boiled at 100 °C for 10 min. Then, 20 µg of protein from each sample was loaded onto a sodium dodecyl sulfate–polyacrylamide gel electrophoresis (SDS‐PAGE) gel and transferred to a polyvinylidene difluoride membrane. The membranes were incubated overnight at 4 °C with primary antibodies, secondary antibodies anti‐rabbit IgG HRP‐conjugated (1: 3000, 7074S, Cell Signaling Technology) and anti‐mouse IgG HRP‐conjugated (1: 3000, 7076S, Cell Signaling Technology) at 25 °C for 2 h. The primary antibodies used were GAPDH monoclonal antibody (1: 1,0000, 60004‐1‐Ig, Proteintech), CD47 antibody (1: 500, sc‐12730, Santa Cruz), integrin αv antibody (1: 1000, 4711S, Cell Signaling Technology), integrin β3 antibody (1: 1000, 13166S, Cell Signaling Technology). Immunolabeled proteins were detected by enhanced chemiluminescence (170‐5060, BIO‐RAD). And then, protein bands were imaged for densitometric analysis with ChemiDoc Touch (BIO‐RAD) and quantified.

### Cell Viability

Cell viability was measured using the MTT assay kit (C0009, Beyotime), following the manufacturer's protocol.^[^ ^78^ ^]^ Tumor cells were inoculated in 96‐well plates at a density of 1 × 10^5^ cells per well and cultured overnight at 37 °C. Next, cells were treated with cilengitide, Cyclo(─RGDfK), RGDS, or PSFL‐NK13 and incubated for 48 h. Then 10 µL of MTT (ST316, Beyotime) was added to each well and incubation was continued for 4 h. The absorbance was measured at 570 nm using a microplate reader (Varioskan LUX, Thermo). Cell viability was estimated by comparing the relative absorbance values with those of the untreated samples.

### Flow Cytometry

Tumor cells (1 × 10^5^ cells per well) were seeded in 12‐well plates and exposed to different concentrations of peptides, antibodies, or small molecules for 48 h.^[^ ^79^ ^]^ The supernatant was then removed and the cells were digested with 0.25% trypsin (25200‐072, Gibco). After washing, the cells were resuspended in 100 µL of PBS (ST476, Beyotime). For surface staining, cells were labeled with fluorescein isothiocyanate (FITC) antihuman αvβ3 antibody (304402, Biolegend) or APC‐CD47 (17‐0479‐42, Invitrogen). Antibodies used in this study were listed in Table S4 (Supporting Information). Cell suspensions were incubated with the appropriate antibodies for 30 min at room temperature in the dark, followed by a washing step to remove unlabeled antibodies. Flow cytometry analysis was performed using BD LSRFortessa (BD Biosciences) and analyzed by FlowJo software.

### Plasmids, siRNAs, and Transfections

Plasmids for ITGAV pLVX‐IRES‐Neo‐Flag, ITGB3 pCR‐Bluntll‐TOPO, and CD47 pOTB7 were purchased from YouBio, and the cDNAs were subcloned into the pcDNA3.1(+) vector to obtain pcDNA3.1–hITGAV–Flag, pcDNA3.1–hITGB3–Myc, and pcDNA3.1–hCD47–HA. Plasmids expressing HA–CD47Δ^26–32^, HA–CD47^Δ44–50^, HA–CD47^Δ51–55^, HA–CD47^Δ62–67^, HA–CD47^Δ69–73^, and HA–CD47^Δ80–85^, were mutagenized using the KOD‐plus kit (94090, Toyobo) modified from the pcDNA3.1–hCD47–HA plasmid (primer sequences for plasmid construction are listed in Table S5 (Supporting Information)). These plasmids were transfected in cells using Lipofectamine 3000 (L3000‐015, Invitrogen).

The siRNAs used in this study (see details below) were purchased from Shanghai GenePharma Co. Human‐specific siRNAs targeting αv, β3, α5, β1, β6, CD47, Talin‐1, and Kindlin‐2 were designed and synthesized by GenePharma,^[^ ^80^ ^]^ and the siRNA sequences are shown in Table S6 (Supporting Information). Lipofectamine RNAiMAX (13778075, Invitrogen) was used to transfect 100 nm of siRNA or nonsilencing siRNA (control siRNA) targeting these genes was transfected into tumor cells. The medium was changed after 6 h of transfection and the culture was continued for 48 h. The knockdown efficiency of RNA was evaluated by RT‐qPCR assay, and the overexpression level of target proteins was determined by Western blotting.

### shRNAs and Lentivirus Vector Transfection

Lentivirus packaging was made by GenePharma.^[^ ^81^ ^]^

To generate lentivirus expressing shRNA targeting human ITGAV and ITGB3, the following sequences were used: 5′‐CCCUCUGACAUUGAUUGUUTT‐3′ (shαv) and 5′‐CCAACAACCCACUGUAUAATT‐3′ (shβ3). Lentiviruses were used to achieve stable knockdown of integrin subunits αv or β3, as well as double knockdown of αvβ3, and to generate stable cell lines. Tumor cells were transduced with a lentiviral vector. Briefly, 1 × 10^5^ tumor cells were transfected with lentiviral vectors at 37 °C with 5 µg mL^−1^ polyethylene glycol (GenePharma) overnight. Transfected tumor cells were cultured in selection medium containing puromycin (ST551, Beyotime) for 2 weeks to select stable cell lines.

### THP‐1 Monocyte‐Derived M0 Macrophages and Peripheral‐Blood‐Monocyte (PBMC‐Derived Mφ Macrophages

A model of macrophage polarization was developed starting with the differentiation of human THP‐1 monocytes into M0 macrophages.^[^ ^82^ ^]^ Briefly, 5 × 10^5^ THP‐1 cells were inoculated in 12‐well plates and cultured with 200 ng mL^−1^ PMA (P8139, Sigma‐Aldrich) for 48 h to differentiate into macrophages.

For PBMC‐derived Mφ macrophages, anticoagulant‐treated fresh blood samples were collected from three healthy volunteers. PBMCs were purified by serial density gradients with Ficoll (P1644, Sigma‐Aldrich).^[^ ^83^ ^]^ PBMCs were then cultured in RPMI‐1640 medium + 10% FBS; unless otherwise stated, macrophages used for all in vitro phagocytosis assays were stimulated with 25 ng mL^−1^ of human recombinant M‐CSF (300‐25‐10 UG, PeproTech) on day 2 of differentiation until used on day 9.

### In Vitro Phagocytosis Assay—Phagocytosis Assay

Tumor cells, except for MDA‐MB‐231‐Cherry cells, were labeled with 2 µm of the Protonex Red 600 (21207, AAT Bioquest) for 30 min in the dark. The fluorescence of Protonex Red dyes increased dramatically with decreasing pH from neutral to acidic, according to the manufacturer's protocol.^[^ ^84^ ^]^ Tumor cells were labeled with 0.5 µm of the Protonex Green 500 (21215, AAT Bioquest) for 30 min in the dark. For the in vitro phagocytosis assay, 100 000 macrophages were placed in each well of a 24‐well culture plate. Add 100 000 pHrodo‐Red‐labeled tumor cells to serum‐free culture medium. The cells were gently centrifuged at 200 *g* for 5 min to promote timely settlement tumor cells to the same plane as the attached macrophages. THP‐1–M0‐phagocytosis test plates were then placed in an incubator at 37 °C and imaged at 0–36 h intervals. Whole‐cell phagocytosis was assessed on an inverted fluorescence microscope (DMI3000 B, Leica) and high‐resolution images were taken. For phagocytosis assays involving treatment with antibodies or peptides, after coculture, phagocytosis assays were stopped by placing plates on ice, centrifuged at 400 *g* for 5 min at 4 °C. Assays were analyzed by flow cytometry on an BD LSRFortessa and analyzed by FlowJo software, using a high‐throughput autosampler. Phagocytosis was measured as the number of pHrodo Red+ macrophages, quantified as a percentage of the total macrophages.

Table S4 (Supporting Information) summarizes the antibodies and isotype controls used in this study, and Figure S9H (Supporting Information) shows example gating.

### In Vitro Phagocytosis Assay—Bone‐Marrow‐Derived Macrophages

Bone marrow cells were isolated from the femurs of 6–8 week old NSG mice posteuthanasia via cervical dislocation.^[^ ^85^ ^]^ Harvested cells were centrifuged at 1000 rpm and cultured in complete RPMI medium containing 10% FBS, 1% l‐glutamine, 1% penicillin–streptomycin, and supplemented with 20 ng mL^−1^ recombinant M‐CSF. Cells were incubated at 37 °C with 5% CO_2_ for 7 days, with medium refreshed on days 2, 5, and 7. Macrophage differentiation was confirmed prior to downstream phagocytic analyses.

### In Vitro Phagocytosis Assay—Peritoneal Macrophages

To isolate peritoneal macrophages, 6–8 weeks old NSG mice were euthanized by cervical dislocation.^[^ ^86^ ^]^ A 5 mL aliquot of complete medium was injected into the peritoneal cavity and allowed to dwell for 5 min. Exudates were aspirated using a 10 mL syringe and centrifuged at 1000 rpm. Cell pellets were resuspended in complete medium (10% FBS, 1% glutamine, 1% penicillin–streptomycin) and allowed to adhere overnight prior to experimentation.

### Fluo‐4 AM Fluorescence for Intracellular Ca^2+^ Analysis

Tumor cells were placed in 12‐well plates, and incubated for 48 h at 37 °C. The intracellular concentration of free Ca^2+^ was measured using the fluorescent Ca^2+^ probe Fluo‐4 AM (S1060, Beyotime).^[^ ^87^ ^]^ Cells were incubated in PBS with 2 µm Fluo‐4 AM at 37 °C for 30 min in the dark according to the protocol of the kit, followed by three washes with PBS; 500 µL of PBS containing 1% FBS was added and incubated for 30 min in the dark at room temperature. Fluorescence intensity of Fluo‐4 was assessed by flow cytometry (BD LSRFortessa) with an excitation wavelength of 488 nm and an emission wavelength of 520 nm.

### CD47–SIRPα/αvβ3 Interaction Model, Enhanced Conformational Sampling, and BRET Assays—Z Dock

1) for the human CD47–SIRPα (PDB No. 2JJT) and human αvβ3 (PDB No. 1JV2) structures, the missing side chains in the structures were filled, hydrogen was added, and energy minimization was optimized; 2) the prepared CD47–SIRPα complex and αvβ3 were used as initial structures and the default parameters of Z‐dock were selected (angle step, 6°; Z‐rank to obtain 2000 vertex positions; filter position cutoff, 10 Å).^[^ ^88^ ^]^ Based on the underlying understanding of αvβ3 and CD47–SIRPα interactions (Figures S6B and S14, Supporting Information): the upper region of αvβ3 away from the cell membrane, including the extracellular structural domain of CD47, and also the transmembrane region of αvβ3 and CD47 were excluded during the exhaustive matching of in silico Z‐docking; 3) clustering of the 2000 top positions (RMSD cutoff, 10 Å, interface cutoff, 10 Å); 4) based on the conformation of the specific interaction between αvβ3 and CD47–SIRPα and the underlying understanding of pairwise recognition involving macrophages and tumor cells, the 2000 top positions after clustering were manually selected to obtain the best CD47–SIRPα/αvβ3 interaction model for subsequent conformational sampling and mutagenesis and Co‐IP confirmation.

### CD47–SIRPα/αvβ3 Interaction Model, Enhanced Conformational Sampling, and BRET Assays—CMD Simulations

CMD was run using Desmond, a high‐performance dynamic computing program from D. E. Shaw,^[^ ^89^ ^]^ and optimized for the CD47–SIRPα/αvβ3 model. 1) The αvβ3 and CD47–SIRPα complexes obtained from Z‐Dock were preprocessed to fix the bonding information and add hydrogen atoms, and the OPLS 2005 all‐atomic force field was based on constraint minimization to be achieved. 2) The establishment of a simulation system for CD47–SIRPα/αvβ3 complex: the SPC model was chosen as the water molecule model, a cube box with a buffer size of 10 Å was selected, and 150 mm NaCl was added to simulate the salt concentration and charge under physiological conditions; through Desmond's default parameters and optimization process, convergence and finite energy minimization based on the OPLSA all‐atom force field system was performed. 3) The combination was chosen for *NPT* with a temperature of 300 K, pressure of 1 bar, and simulation duration of ≈300 ns. During the simulation, the conformations were continuously extracted and observed to see if they were optimized to a reasonable degree to yield the most reasonable interaction mode between αvβ3 and CD47–SIRPα.

### CD47–SIRPα/αvβ3 Interaction Model, Enhanced Conformational Sampling, and BRET Assays—REST Enhanced Sampling

1) Solve (REST) was selected in the Template Method Options panel and the Number of Copies text box was specified as 32; 2) the temperature in the temperature range text box screen was set to range from 300 to 372 K; 3) “Replica Exchange” was used to generate a trajectory for each replica and view the results in the “Replica Exchange review” panel, and simulation duration of ≈30 ns.

### CD47–SIRPα/αvβ3 Interaction Model, Enhanced Conformational Sampling, and BRET Assays—BRET‐Based Analysis of CD47/αvβ3 Interactions

BRET, a noninvasive and highly quantitative method for probing protein–protein interactions in live cells, relied on the transfer of energy from a bioluminescent donor (luciferase) to a proximal fluorescent acceptor.^[^ ^55^ ^]^ To interrogate the spatial proximity and interaction dynamics between CD47 and the αvβ3 integrin complex, CD47 was tagged at its C‐terminus with the donor Rluc8, while ITGαv and ITGβ3 subunits were each tagged at their C‐termini with eYFP. Cotransfection with varying donor‐to‐acceptor ratios revealed significant BRET signals, confirming energy transfer between CD47–Rluc8 and both ITGαv–eYFP and ITGβ3–eYFP, and thereby validating heterodimeric complex formation under physiological expression conditions.

### Co‐Immunoprecipitation

MDA‐MB‐231 cells or HEK293 cells were incubated with Pierce IP lysis buffer (87787, Thermo Fisher) and protease inhibitor cocktail (B14001, Bimake) at 4 °C for 15 min.^[^ ^90^ ^]^ After centrifugation at 12 000 *g* for 15 min at 4 °C, supernatant MDA‐MB‐231 cells were prepared for immunoprecipitation and incubated with Pierce Protein A/G magnetic beads (Pierce Classic Magnetic IP/Co‐IP Kit, 88804, Thermo Fisher) and anti‐CD47 antibody (323102, Biolegend), or mouse IgG1 (G3A1) isotype control (5415S, Cell Signaling Technology) and incubated overnight at 4 °C. The magnetic bead–antigen–antibody complexes were magnetically separated and washed 3 times with IP lysis/wash buffer. The complexes were added to 100 µL of lane labeled sample buffer and boiled for 10 min. The supernatant of HEK293 cells was incubated with mouse mAb IgG XP isotype control (magnetic bead conjugate, 5873, CST) or anti‐Flag magnetic beads (B26101, Bimake) overnight at 4 °C. Magnetic‐bead‐based immunoprecipitation was performed according to the manufacturer's instructions. After washing 3 times with buffer, the magnetic beads were incubated with 1× sample loading buffer and boiled for 10 min, and the supernatant was then analyzed by SDS‐PAGE and immunoblotting.

### In Vivo Tumor‐Growth Experiments

Mice were housed under standard pathogen‐free conditions and all animal experiments were performed according to protocols approved and authorized by the Animal Welfare and Ethics Committee of China Pharmaceutical University. 6–8 weeks old female nude mice were injected with 2 × 10^6^ of MDA‐MB‐231‐shNC or MDA‐MB‐231‐shαv cells at the fourth inguinal mammary glands. Tumor size was measured every two days after transplantation (8–10 mice per group), and tumor volume (mm^3^) was defined as follows: π × (length) × (width)^2^/6. Mice were killed 4 weeks after injection and orthotopic tumors were harvested for further study.^[^ ^91^ ^]^

MDA‐MB‐231‐shNC cells (2 × 10^6^) suspension was injected into the fourth mammary fat pad in 6–8 weeks old female nude mice. 6–8 weeks old female nude mice were injected subcutaneously with HCT116 cells (1 × 10^6^). 6–8 weeks old female BALB/c mice were injected with 4T1 cells (1 × 10^6^) suspension into the fourth mammary fat pad. Tumor growth was routinely measured using standard calipers, and tumor volume (mm^3^) was calculated using the formula π × (length) × (width)^2^/6. When the volume of mice reached ≈100 mm^3^, they were randomly divided into two or four groups and treated with the peptide PSFL‐NK13.^[^ ^92^ ^]^ For the MDA‐MB‐231 model, 0.9% saline or PSFL‐NK13 (0.5, 2, and 8 mg kg^−1^ intravenously) was given every two days; for the HCT116 and 4T1 models, 0.9% saline or PSFL‐NK13 (2 mg kg^−1^ intravenously) was given once daily. Tumor‐bearing mice were sacrificed, tumors were extracted, weighed, manually homogenized and diluted in PBS. Tumors were then processed for flow cytometry.

### In Vivo Tumor‐Growth Experiments—Survival Assay

To assess the therapeutic efficacy of PSFL‐NK13, 6–8 weeks old NSG mice (NOD.Cg‐PrkdcscidIl2rgem1Smoc, NM‐NSG‐001; Shanghai Model Organisms Center, Inc.) were subcutaneously implanted with 4 × 10⁶ MDA‐MB‐231 cells in the left flank (100 µL injection volume).^[^ ^93^ ^]^ Upon tumor establishment (≈200 mm^3^), animals were randomized and treated with either 2 or 8 mg kg^−1^ PSFL‐NK13 via peritumoral injection every 48 h. Tumor dimensions were recorded bidaily using precision calipers. Mice were euthanized upon reaching predefined ethical endpoints—tumor diameter >20 mm or volume >2000 mm^3^—and survival was analyzed using Kaplan–Meier estimates. Tumor volume was calculated as follows: π × (length) × (width)^2^/6.

### In Vivo Tumor‐Growth Experiments—Macrophage Depletion Assay

NSG mice (6–8 weeks old) were similarly inoculated with 4 × 10⁶ MDA‐MB‐231 cells in 100 µL into the left flank.^[^ ^94^, ^95^ ^]^ Upon tumor volumes reaching ≈200 mm^3^, mice were assigned to four treatment arms: macrophage depletion agent‐control, macrophage depletion agent‐PSFL‐NK13, PBS‐control, and PBS‐PSFL‐NK13. Mice received 8 mg kg^−1^ PSFL‐NK13 via peritumoral injection every other day and 100 µL of the macrophage depletion agent via intraperitoneal injection every third day. Tumor dimensions were measured with calipers, and tumor growth curves were generated. An identical protocol was employed for BALB/c mice (6–8 weeks old) subcutaneously implanted with 4 × 10⁶ 4T1 cells.

### Fluorescence‐Activated Cell Sorting (FACS) of Tumor Samples

Grinding the same portion of tumor tissue (FSTR070, Beyotime) and single‐cell suspensions of tumor samples were obtained. 1 mL of 1× red blood cell (RBC) lysis buffer (420301, Biolegend) was added and lysed for 5 min according to the manufacturer's instructions.^[^ ^96^ ^]^ The samples were then resuspended in FACS buffer at a concentration of 1 million cells per mL and the antibody plates were stained for 30 min at room temperature, followed by two washes with FACS buffer. The antibody plates, their fluorophores and the purpose of use are listed in Table S4 (Supporting Information). Flow cytometry was performed on a BD LSRFortessa analyzer, and all flow cytometry data obtained here were analyzed with FlowJo.

### Hemagglutination Assay

Blood samples from three healthy volunteers were collected, treated with anticoagulant, and mixed with human lymphocyte isolation medium (P1644, Sigma‐Aldrich).^[^ ^97^ ^]^ The anticoagulant‐treated fresh blood was centrifuged and the supernatant was removed, and after separating and washing twice (centrifugation at 400 *g* for 20 min), RBCs were mixed with phosphate buffer to generate a 2.5% v/v cell suspension. Then, 50 µL of RBCs were added to a 96‐well round‐bottom plate and mixed with 50 µL of various amounts of antibodies or peptides. Hemagglutination was defined by the red floc in the supernatant, and no significant change was defined by the colorless supernatant and the sinking of whole RBCs.

### Tube Formation Assay

48‐well plates were coated with 120 µL of Matrigel substrate (354234, Corning) per well and incubated for 1 h at 37 °C.^[^ ^98^ ^]^ HUVECs were seeded at a density of 4 × 10^4^ cells and pretreated with 50 ng mL^−1^ VEGF (100‐20‐10 UG, PeproTech) for 10 min. Then, different concentrations of cilengitide or PSFL‐NK13 were added to the culture medium of HUVECs for 6 h. The capillary‐like structures formed by HUVECs were recorded by microscopy and the number of tubes was counted.

### Statistical Analysis

Data were expressed as mean ± standard error of the mean (s.e.m.). All experiments were performed with at least 3 independent experiments. Statistical analyses were performed as described in each corresponding figure legend. Differences between two groups were assessed by unpaired Student's *t*‐test, and differences between multiple groups were assessed by one‐way ANOVA and Dunnett's or Bonferroni post‐hoc test, and *p* < 0.05 was considered statistically significant.

## Conflict of Interest

The authors declare no conflict of interest.

## Author Contributions

P.‐C.Y., C.‐X. Y., W.‐Z.D., and C.‐Y.H. contributed equally to this work. Y.‐Z.F. and Y.Y. designed the project; P.‐C.Y., C.‐Y.H., Y.‐F.Q., D.L., J.‐B.Y., and Y.‐Z.F. performed cell culture, cell viability, siRNAs and shRNA transfections, in vitro phagocytosis assay, flow cytometry, western blotting; Y.‐Z.F. and Y.Y. performed clinical tissues analysis; P.‐C.Y., C.‐X.Y., and Y.‐Z.F. performed tube formation assay and hemagglutination assay; W.‐Z.D., C.‐Y.H., Y.‐F.Q., D.L., Q.Z., and J.B.Y. performed animal experiments; C.‐Y.H., X.Z., and D.P.W. did the truncation of CD47; P.C. and Y.Y. did CMD and REMD simulations; Y.Y. performed peptide design; P.‐C.Y., Y.‐Z.F., and Y.Y., analyzed data; P.‐C.Y., Y.‐Z.F., Y.Y. wrote the paper. All authors discussed the results and commented on the paper.

## Supporting information



Supporting Information

## Data Availability

Research data are not shared.
