# Supplementary material for: Cancer Immunotherapy via Disruption of Integrin αvβ3 and CD47 Costabilization on Cancer Cell Surface
Source: Adv Sci (Weinh). 2025 Oct 30;13(2):e01602. doi: 10.1002/advs.202501602 (PMC12786276; doi:10.1002/advs.202501602)
Supplement: Supplementary file 1 — Supporting Information [file ADVS-13-e01602-s001.pdf]

Supplementary figures S1-21

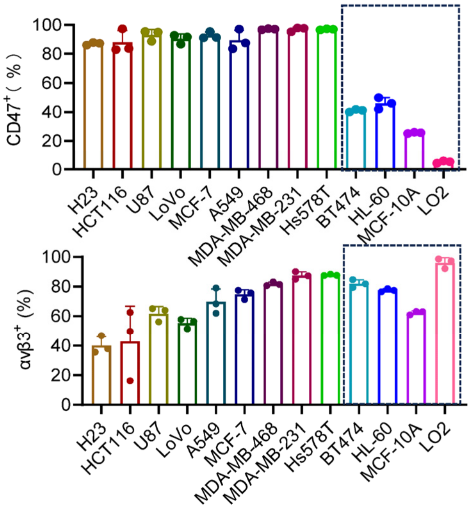

Supplementary Figure S1. Quantitative flow cytometric profiling of αvβ3 and CD47 surface expression across diverse human cell lines. A cohort comprising ten tumor cell lines and three non-malignant counterparts was analyzed to assess surface levels of integrin αvβ3 and CD47. Composite expression data for CD47 (top panel) and ITG αvβ3 (bottom panel) are displayed as mean ± standard error of the mean (s.e.m.), derived from three independent biological replicates.

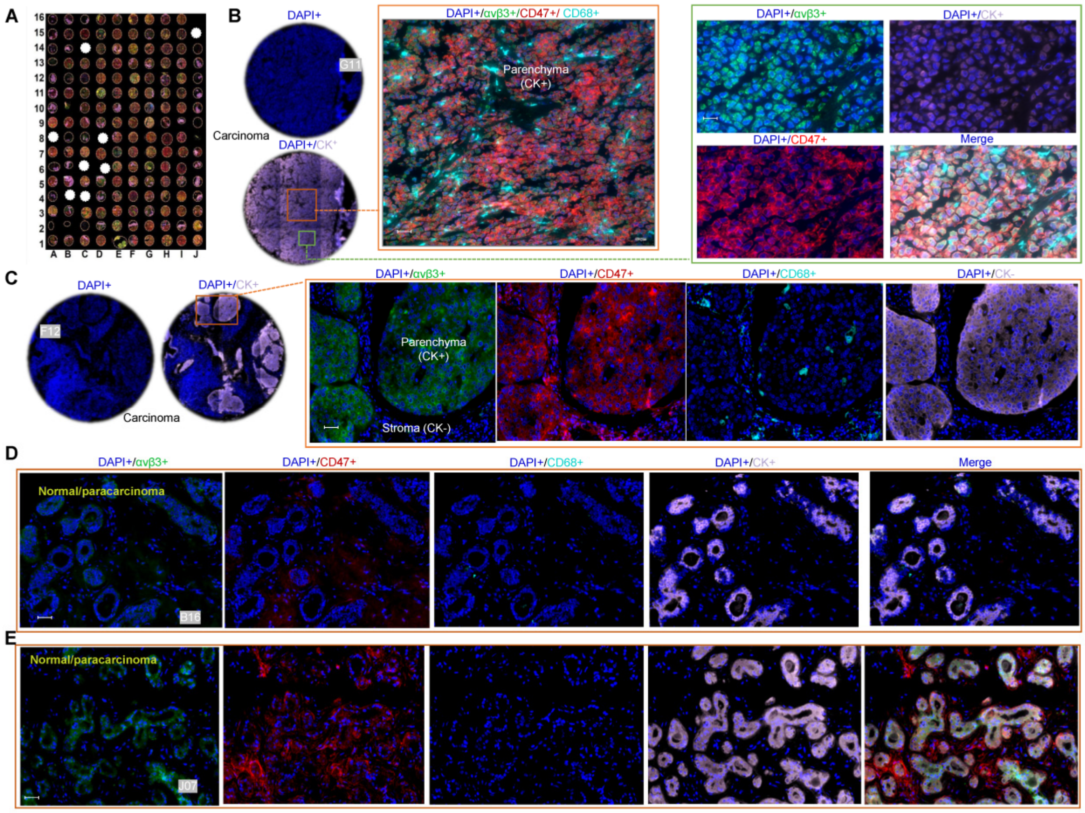

Supplementary Figure S2. Multiplex fluorescence immunohistochemical staining in 119 breast cancer clinical samples and 32 corresponding paracancerous tissues or normal tissues. (A) Schematic representation of tissue microarrays (TMA) comprising clinical breast cancer tissue samples and paired paracancerous or normal tissues. (**B**, **C**) Multiplex fluorescence immunohistochemical staining and imaging of DAPI+ (blue), αvβ3+ (green), CD47+ (red), CD68+ (sky blue), and CK+ (grayish purple) in breast cancer tissues, with magnified area of the section shown in the right panel. Scale bar = 50 μm. (**D**, **E**) Simultaneous localization of multiplex fluorescent immunohistochemical staining and imaging in normal/paraneoplastic tissues, with merged images shown in the right panel. Scale bar = 50 μm.

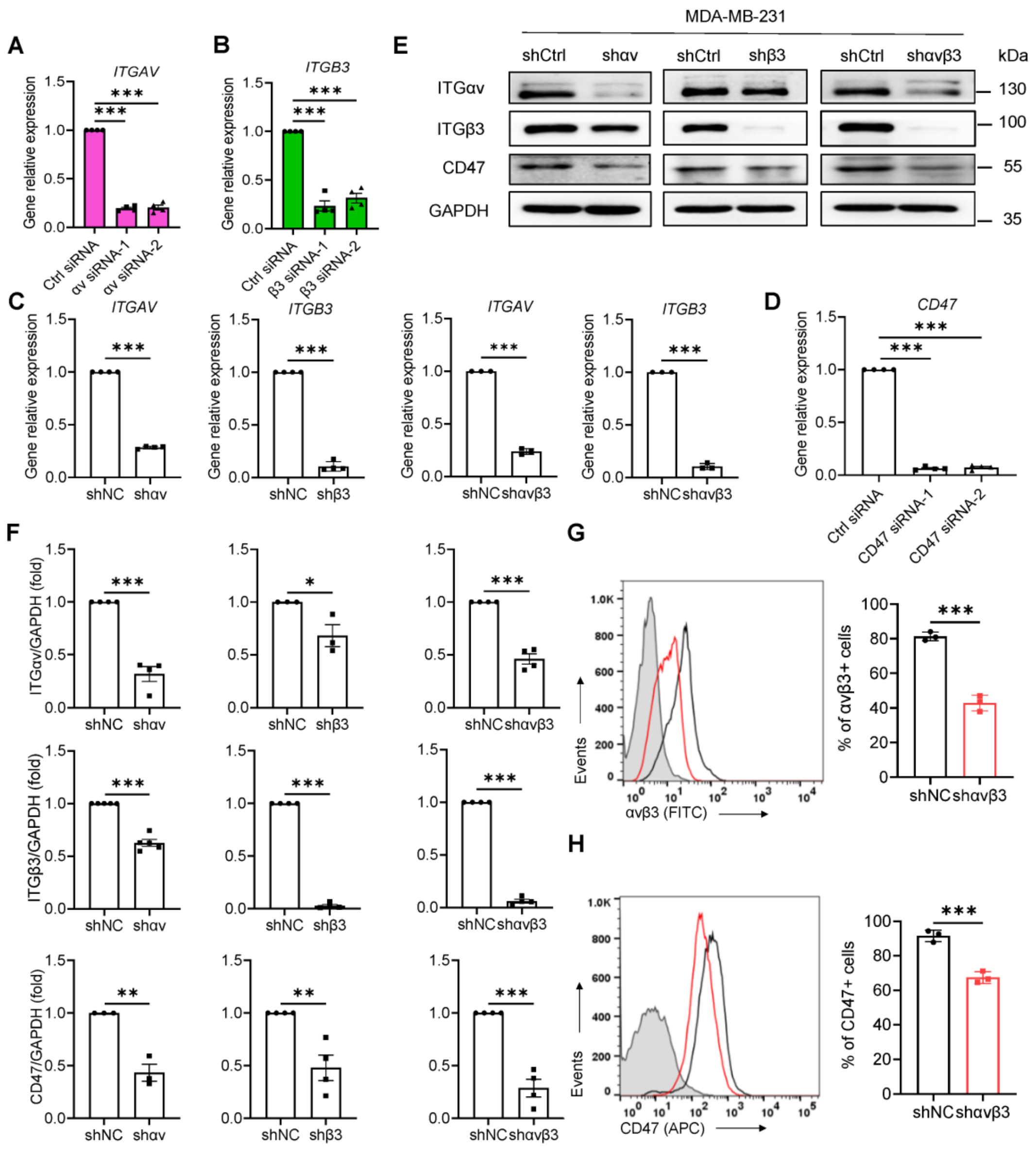

Supplementary Figure S3. Knockdown of αvβ3 reduces CD47 mRNA and protein expression. (**A**, **B**) RT-qPCR analysis of ITGAV and ITGB3 mRNA levels in MDA-MB-231 cells after siRNA-mediated knockdown of integrin subunits αv or β3 for 48 hours. (**C**) RT-qPCR analysis of ITGAV and ITGB3 mRNA levels in MDA-MB-231 cells after stable knockdown of integrin subunits αv or β3, and lentiviral double knockdown of αvβ3. (**D**) RT-qPCR analysis of CD47 mRNA levels in MDA-MB-231 cells 48 hours post-siRNA-mediated CD47 knockdown. (**E**, **F**) Western blot analysis (E) and pooled data of ITGαv, ITGβ3, and CD47 protein expression levels (F). Data are presented as induction rates of GAPDH control in triplicate. (**G, H**) Flow cytometry analysis of CD47 and

αvβ3 surface expression on MDA-MB-231 cells following stable double knockdown of integrin αvβ3 by lentivirus, with pooled data of cell surface markers αvβ3 and CD47. All data are expressed as mean ± s.e.m, n = 3-4 independent experiments; $^{*}P < 0.05$, $^{**}P < 0.01$, $^{***}P < 0.001$ versus control, unpaired t test (C, F, G and H); one-way ANOVA with Dunnett's post-hoc test (A, B and D); (A), ($F_{(2, 9)} = 806.8$, $P < 0.0001$), (B), ($F_{(2, 9)} = 101.8$, $P < 0.0001$). (D), $F_{(2, 9)} = 4849$, $P < 0.0001$.

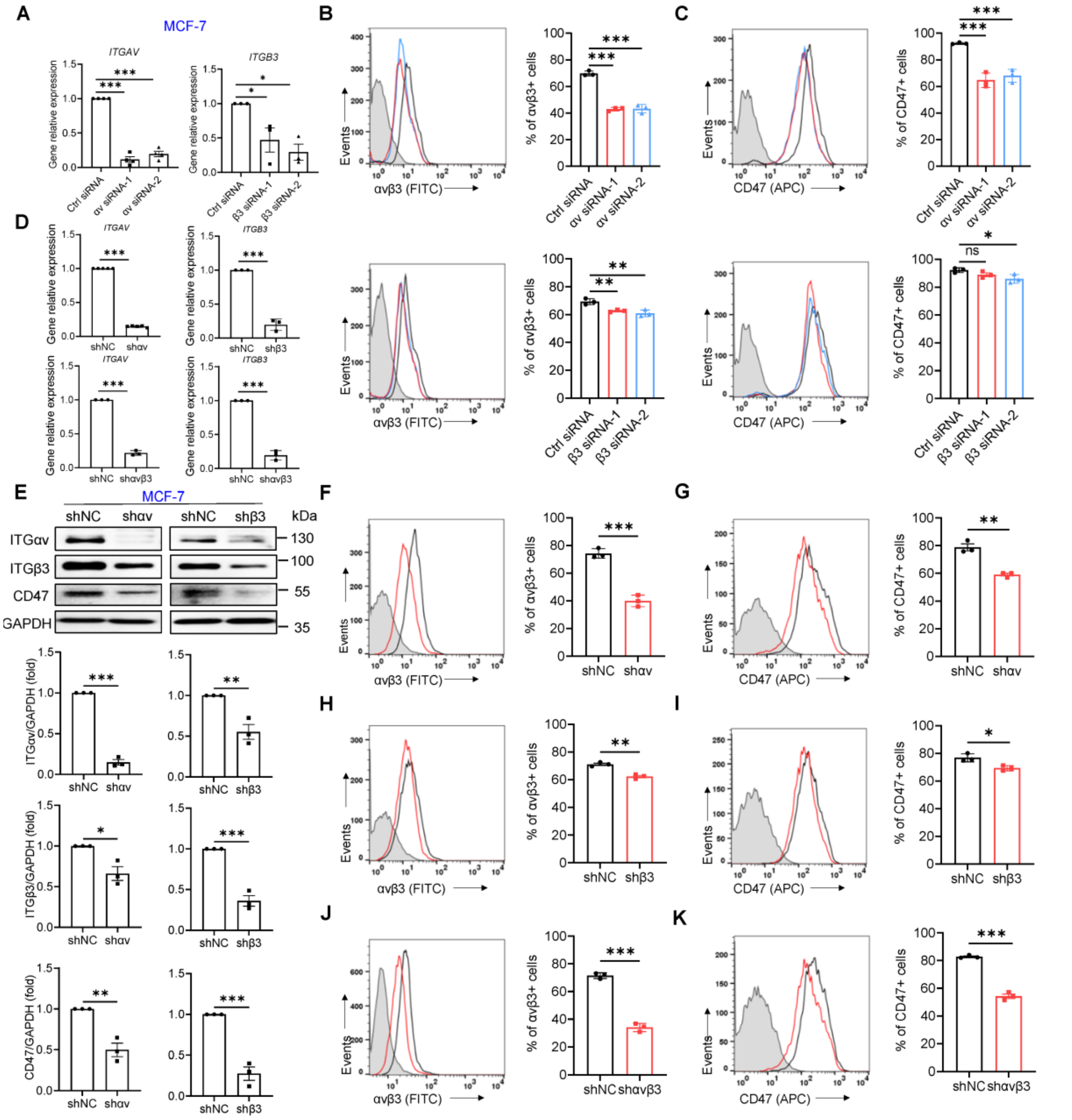

Supplementary Figure S4. Knockdown of αvβ3 reduces CD47 surface expression in MCF-7 Cells. (**A**) RT-qPCR analysis of ITGAV and ITGB3 mRNA expression in MCF-7 cells 48 hours post-siRNA knockdown of αv or β3 integrin subunits. (**B**, **C**) Flow cytometry analysis of αvβ3 and CD47 surface expression in MCF-7 cells 48 hours post-siRNA-mediated knockdown of αv and β3 subunits, with pooled data. (**D**) RT-qPCR analysis of ITGAV and ITGB3 mRNA expression in MCF-7 cells after stable knockdown of αv or β3 integrin subunits and double knockdown of αvβ3 via lentivirus. (**E**) Western blot analysis and pooled data of ITGαv, ITGβ3, and CD47 protein expression levels. The histograms depict GAPDH induction rates in triplicate. (**F-K**) Flow cytometry analysis

of αvβ3 and CD47 surface expression in MCF-7 cells following stable knockdown of αv subunit, β3 subunit, and αvβ3 via lentivirus, with pooled data. All data are expressed as mean ± s.e.m, n = 3-4 independent experiments; $^{*}P < 0.05$, $^{**}P < 0.01$,$^{***}P < 0.001$ versus control, unpaired t test (D-K); one-way ANOVA with Dunnett's post-hoc test (A-C); (A), ($F_{(2, 9)} = 225.2$, $P < 0.0001$), ($F_{(2, 6)} = 9.192$, $p = 0.0149$), (B), ($F_{(2, 6)} = 139.6$, $P < 0.0001$), ($F_{(2, 6)} = 15.94$, $p = 0.0040$); (C), ($F_{(2, 6)} = 38.05$, $p = 0.0004$), ($F_{(2, 6)} = 4.726$, $p = 0.0586$). ns, not significant.

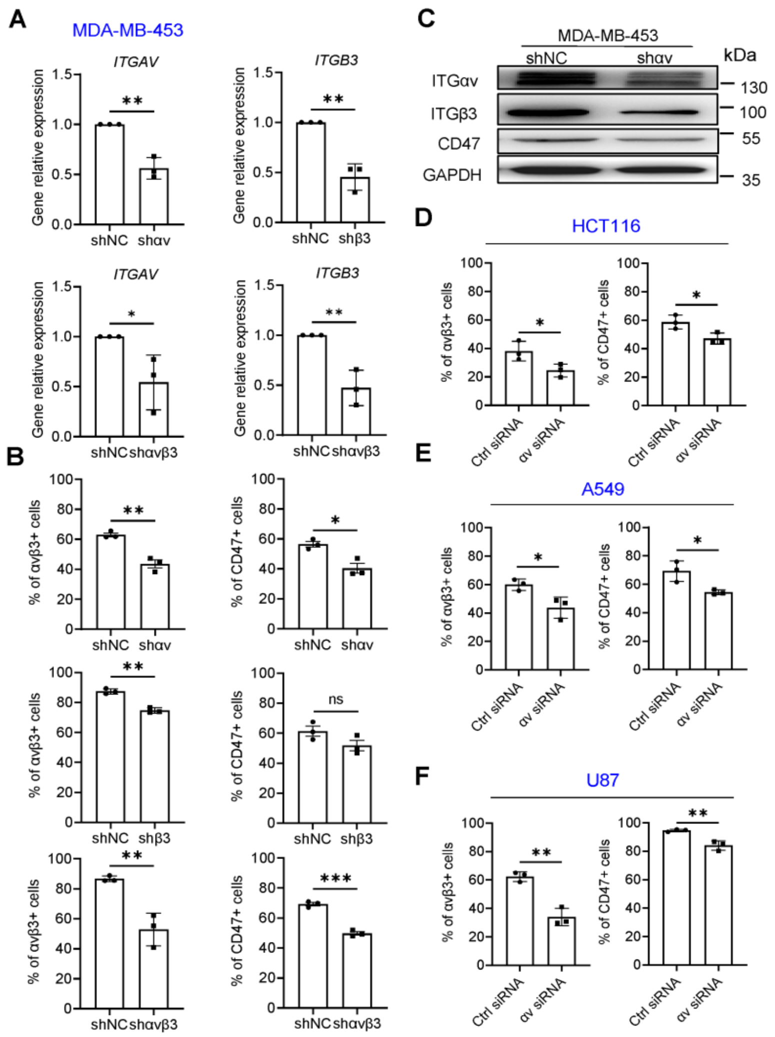

Supplementary Figure S5. αvβ3 knockdown reduces CD47 surface expression in various cancer cells. (**A**) RT-qPCR analysis of ITGAV and ITGB3 mRNA expression in MDA-MB-453 cells post-stable knockdown of αv or β3 integrin subunits, and double knockdown of αvβ3 via lentivirus. (**B**) Flow cytometry analysis of αvβ3 and CD47 surface expression in MDA-MB-453 cells following stable knockdown of αv subunit, β3 subunit, and αvβ3 via lentivirus, with pooled data. (**C**) Western blot analysis of ITGαv, ITGβ3, and CD47 protein expression levels. (**D-F**) Flow cytometry analysis of αvβ3 and CD47 surface expression on HCT116, A549, and U87 cells 48 hours post-siRNA-mediated αv subunit knockdown, with pooled data. All data are expressed as mean ± s.e.m, n = 3 independent experiments; $^{*}P < 0.05$, $^{**}P < 0.01$, unpaired t-test compared to control (A, B and D-F); ns, not significant.

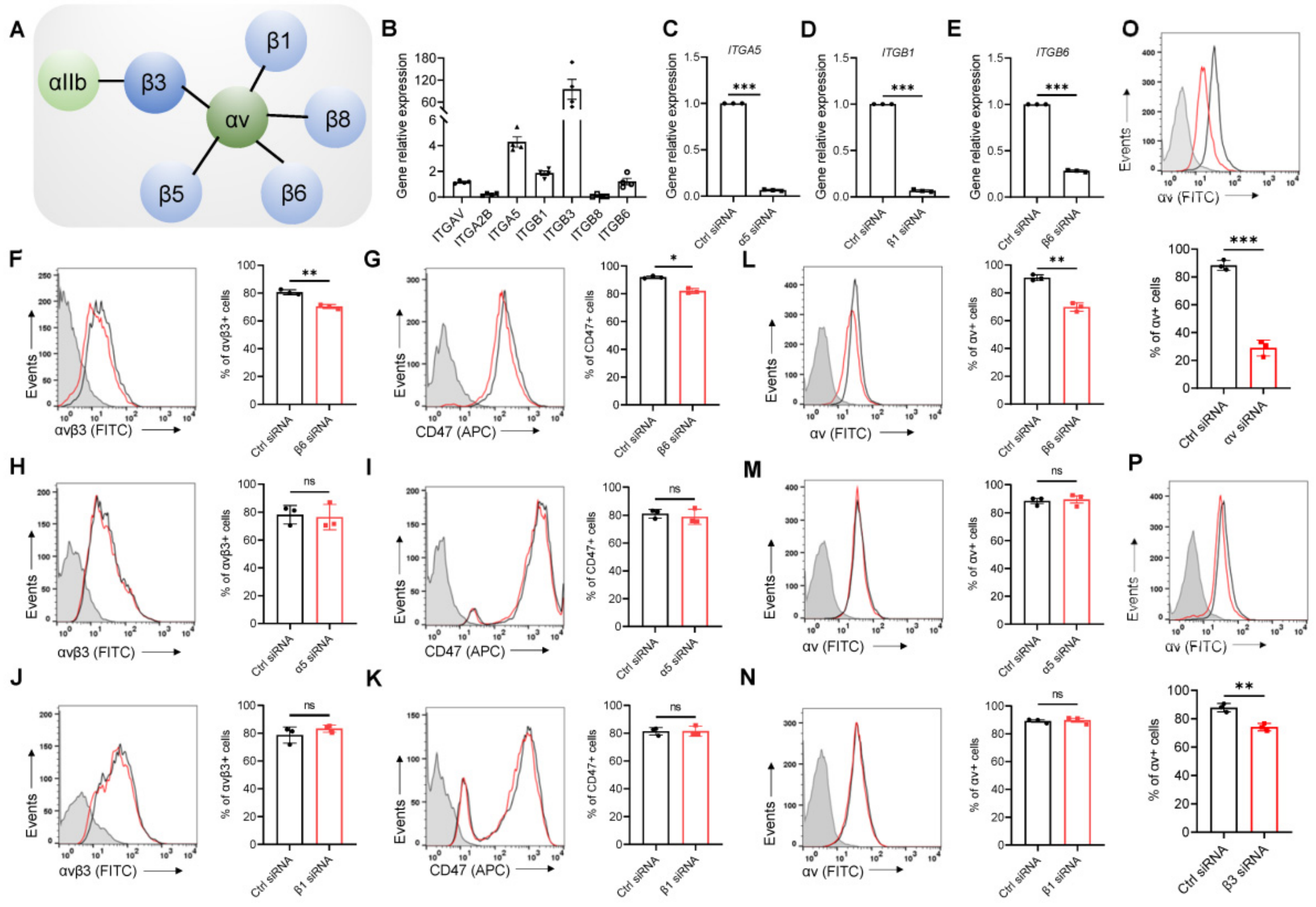

Supplementary Figure S6. Effects of knockdown of other integrin subunits on CD47 surface expression. (**A**) Schematic representation of integrin subunits associated with αv or β3 subunits. (**B**) RT-qPCR analysis of ITGAV, ITGA2B, ITGA5, ITGB1, ITGB3, ITGB6, and ITGB8 mRNA expression in MDA-MB-231 cells.

(**C-E**) RT-qPCR analysis of ITGA5, ITGB1, and ITGB6 mRNA expression in MDA-MB-231 cells post-siRNA-mediated knockdown. (**F-K**) Flow cytometry analysis of αvβ3 and CD47 surface expression in MDA-MB-231 cells post-siRNA-mediated knockdown of β6, α5, and β1 subunits, with pooled data. (**L-P**) Flow cytometry analysis of αv surface expression in MDA-MB-231 cells post-siRNA-mediated knockdown of β6, α5, β1, αv, and β3 subunits, with pooled data. All data are expressed as mean ± s.e.m, n = 3-4 independent experiments; $^{*}P < 0.05$, $^{**}P < 0.01$, $^{***}P < 0.001$ compared to control, unpaired t-test (C-N); ns, not significant.

A

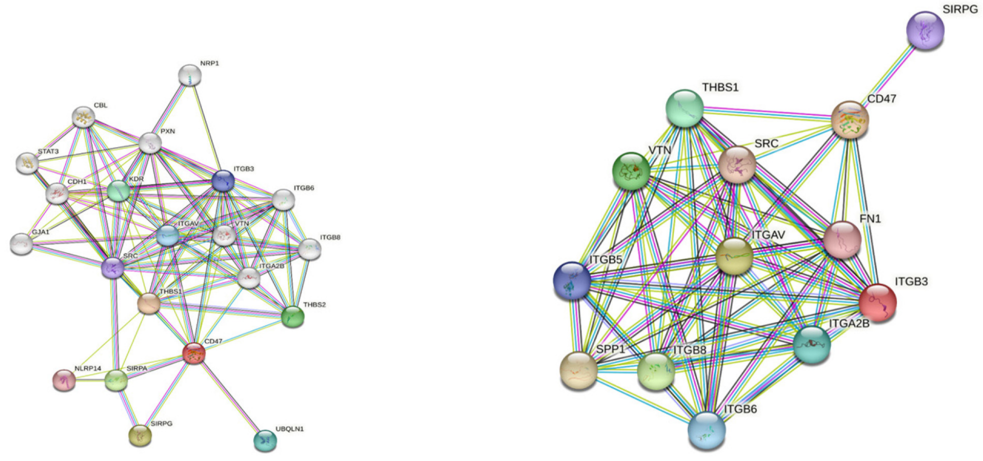

B

node1	node2	node2_string_id	Co-expression	Experimentally determined interaction	Database annotated	Automated text mining	Combined score
CD47	SIRPA	9606.ENSP00000382941	0.062	0.89	0.8	0.995	0.999
CD47	THBS1	9606.ENSP00000260356	0	0.213	0.9	0.989	0.999
CD47	SIRPG	9606.ENSP00000305529	0	0.328	0.8	0.994	0.999
CD47	THBS2	9606.ENSP00000355751	0	0	0.8	0.973	0.994
CD47	KDR	9606.ENSP00000263923	0	0	0	0.986	0.987
CD47	UBQLN1	9606.ENSP00000365576	0.049	0.476	0	0.958	0.977
CD47	ITGAV	9606.ENSP00000261023	0.062	0.213	0.9	0.604	0.966
CD47	ITGB3	9606.ENSP00000452786	0.056	0	0.9	0.66	0.965
CD47	SRC	9606.ENSP00000362680	0	0	0.9	0.569	0.955
CD47	ITGAM	9606.ENSP00000441691	0.059	0	0.9	0.52	0.95
CD47	NLRP14	9606.ENSP00000299481	0	0	0	0.942	0.942
CD47	CD59	9606.ENSP00000379191	0.061	0	0.9	0.441	0.942
CD47	ITGA2B	9606.ENSP00000262407	0	0	0.9	0.387	0.936
CD47	PTPN11	9606.ENSP00000340944	0.062	0	0.6	0.635	0.851
CD47	VTN	9606.ENSP00000226218	0	0	0	0.645	0.645
CD47	FN1	9606.ENSP00000346839	0.058	0	0	0.473	0.482

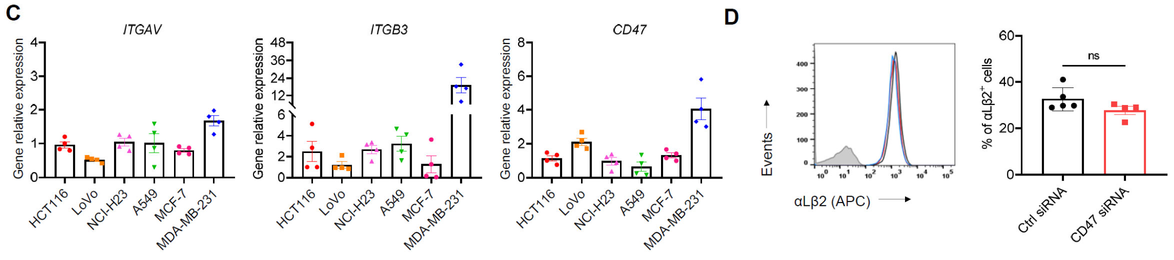

Supplementary Figure S7. STRING-based protein–protein interaction (PPI) analyses provide limited insight into the molecular crosstalk between CD47, αvβ3 integrin, and SIRPα, and do not adequately model their integration into the CD47–SIRPα immune checkpoint pathway. **(A)** A PPI network was constructed using CD47, ITGAV, and ITGB3 as input nodes within the STRING database. Network parameters were configured as follows: full network mode was selected, with edges defined by evidence types. Active interaction sources included text mining, curated databases, experimental data, gene neighborhood, gene fusion, co-expression, and co-occurrence. An interaction confidence threshold of 0.400 was set (medium confidence), and the number of interactors was limited to 20. Proteins are

represented as nodes, with colored connecting lines signifying distinct evidence categories (fusion: red; neighborhood: green; co-occurrence: blue; experimental: purple; text mining: yellow; database: light blue; co-expression: black). **(B)** Interaction confidence table listing the top 16 protein–protein associations, derived from the tandem export file and ranked by STRING score. **(C)** RT-qPCR analysis of ITGAV, ITGB3, and CD47 mRNA levels across a panel of tumor cell lines. Expression data are represented as mean ± s.e.m. from four biological replicates. Correlation between transcript abundance and corresponding cell surface localization of αvβ3 and CD47 is weak or inconsistent. **(D)** Silencing of CD47 via siRNA in MDA-MB-231 cells does not significantly alter the surface expression of αLβ2, as determined by flow cytometry at 48 hours post-transfection. Data are shown as mean ± s.e.m. with statistical analysis performed using unpaired t-test; ns: not statistically significant.

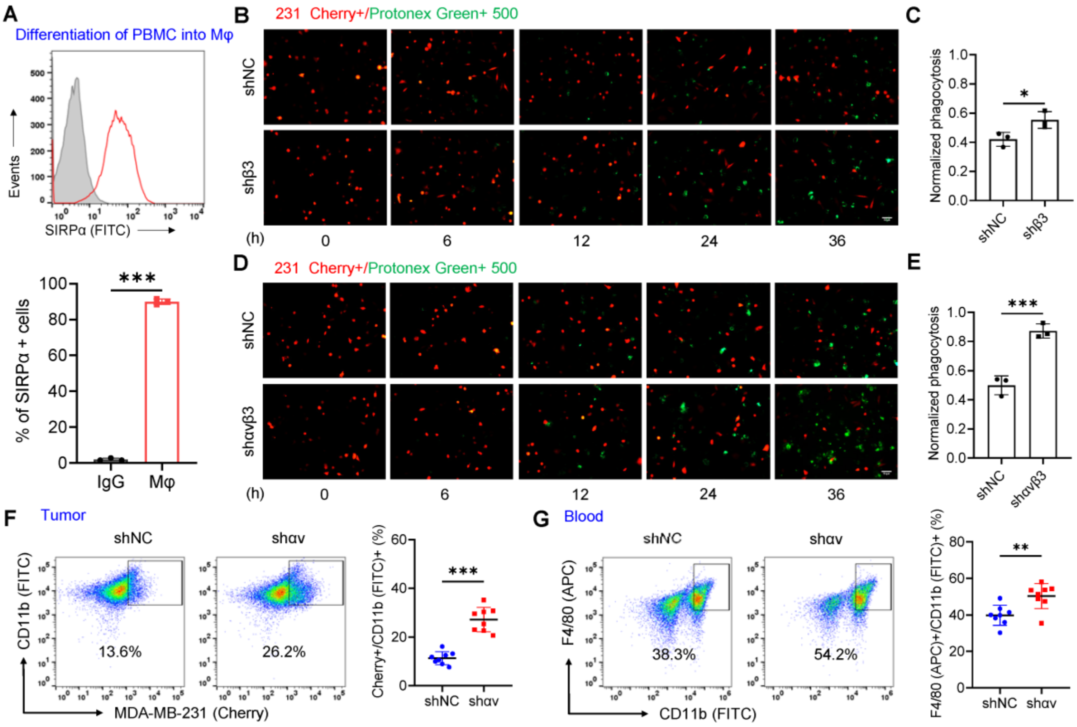

Supplementary Figure S8. Knockdown of αvβ3 enhances macrophage phagocytosis of MDA-MB-231 Cells. (**A**) Peripheral mononuclear blood cells (PBMC)-derived Mφ macrophages were labeled and analyzed by flow cytometry for 9 days to assess SIRPα expression, along with pooled data for the cell surface marker SIRPα. (**B-E**) Representative fluorescence photomicrographs depict phagocytic activity (B, D) and pooled data of mean fluorescence intensity signals in confocal images (C, E). siRNA-mediated knockdown of the integrin β3 subunit or αvβ3 within 0-36 hours enhances phagocytosis analysis of MDA-MB-231-Cherry cells by THP-1-derived macrophages. Scale bar = 50 µm. (**F**) Representative flow cytometry analysis displays the frequency of MDA-MB-231 shNC tumors versus MDA-MB-231 shαv tumors in all macrophage infiltrates 33 days post-transplantation, with macrophage infiltration quantified as MDA-MB-231(cherry)+/CD11b(FITC)+. Similar results were observed in 8 other independent experiments. (**G**) Representative flow cytometry analysis of F4/80+/CD11b+ macrophages in peripheral blood mononuclear cells (PBMCs), with quantification of macrophage ratio assessed as F4/80(APC)+/CD11b(FITC)+ by flow cytometry. Similar results were observed in 8 other

independent experiments. All data are expressed as mean ± s.e.m; $^{*}P < 0.05$, $^{**}P < 0.01$, $^{***}P < 0.001$ versus control, unpaired t-test (A, C, E, F and G).

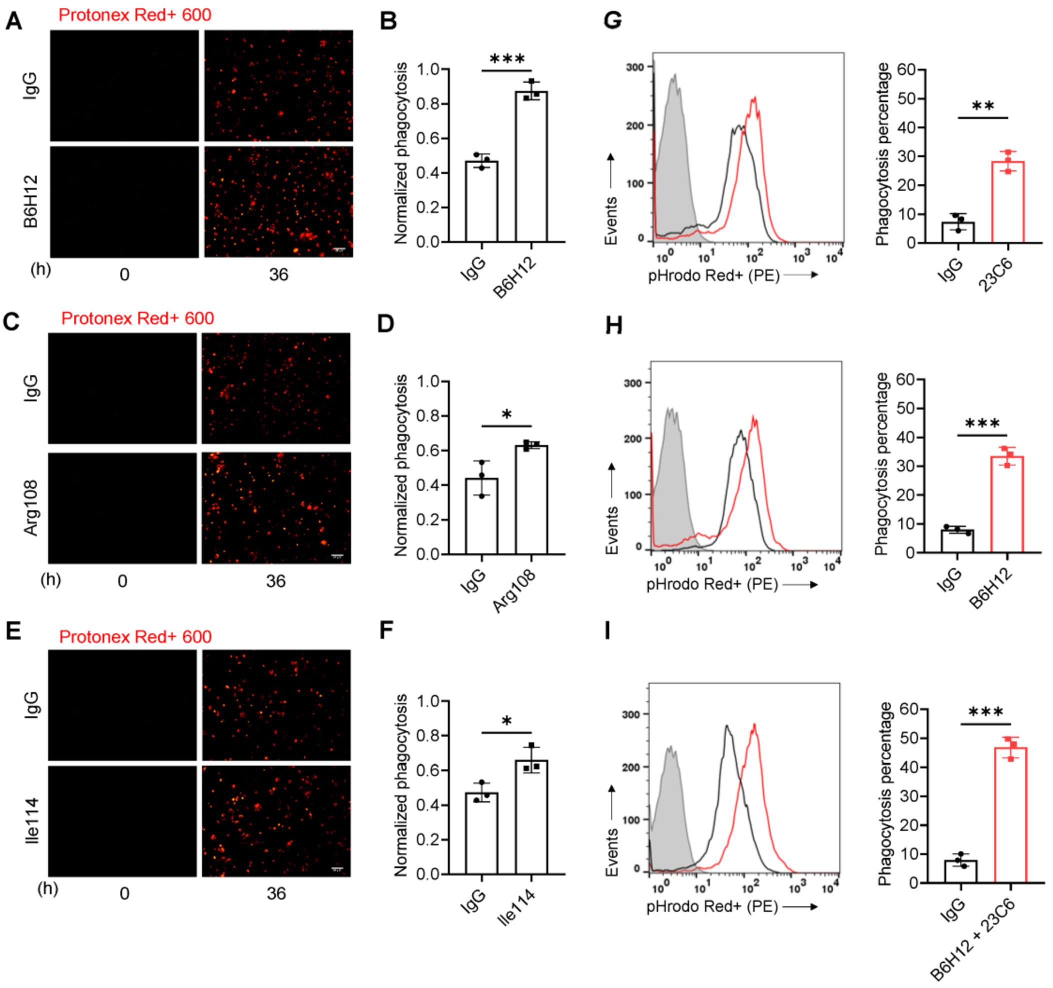

Supplementary Figure S9. Integrin αvβ3 and CD47 antibodies enhance tumor phagocytosis by macrophages. (**A**, **B**) Representative fluorescence microscopy images depict in vitro phagocytosis of MDA-MB-231 cells by macrophages (Protonex Red+, red) in the presence of IgG control or anti-CD47 antibody (B6H12, 10 μg/ml) (A) after 36 hours of co-culture, with statistical analysis of mean fluorescence intensity signal in confocal images (B). Scale bar = 50 μm. (**C-F**) Representative fluorescence microscopy images show macrophages (Protonex Red+, red) phagocytosing MDA-MB-231 cells after 36 hours of co-culture in the presence of IgG control, integrin αv antibody Arg108 (10 μg/ml) (C), or integrin β3 antibody Ile114 (10 μg/ml) (E). Statistical analysis of mean fluorescence intensity signal in the confocal images was performed (D and F). Scale bar = 50 μm. (**G-I**) Flow cytometry-based measurements of co-cultured THP-1-derived macrophages in the presence of αvβ3

antibody (23C6, 10 μg/ml), CD47 antibody (B6H12, 10 μg/ml), or both αvβ3 antibody (23C6, 10 μg/ml) and CD47 antibody (B6H12, 10 μg/ml) on MDA-MB-231 cells by phagocytosis (red line), compared to IgG control (black line). All data are expressed as mean ± s.e.m., n = 3 independent experiments; $^{*}p < 0.05$, $^{**}p < 0.001$, $^{***}p < 0.001$ compared to control, unpaired t-test (B, D, F, G, H and I).

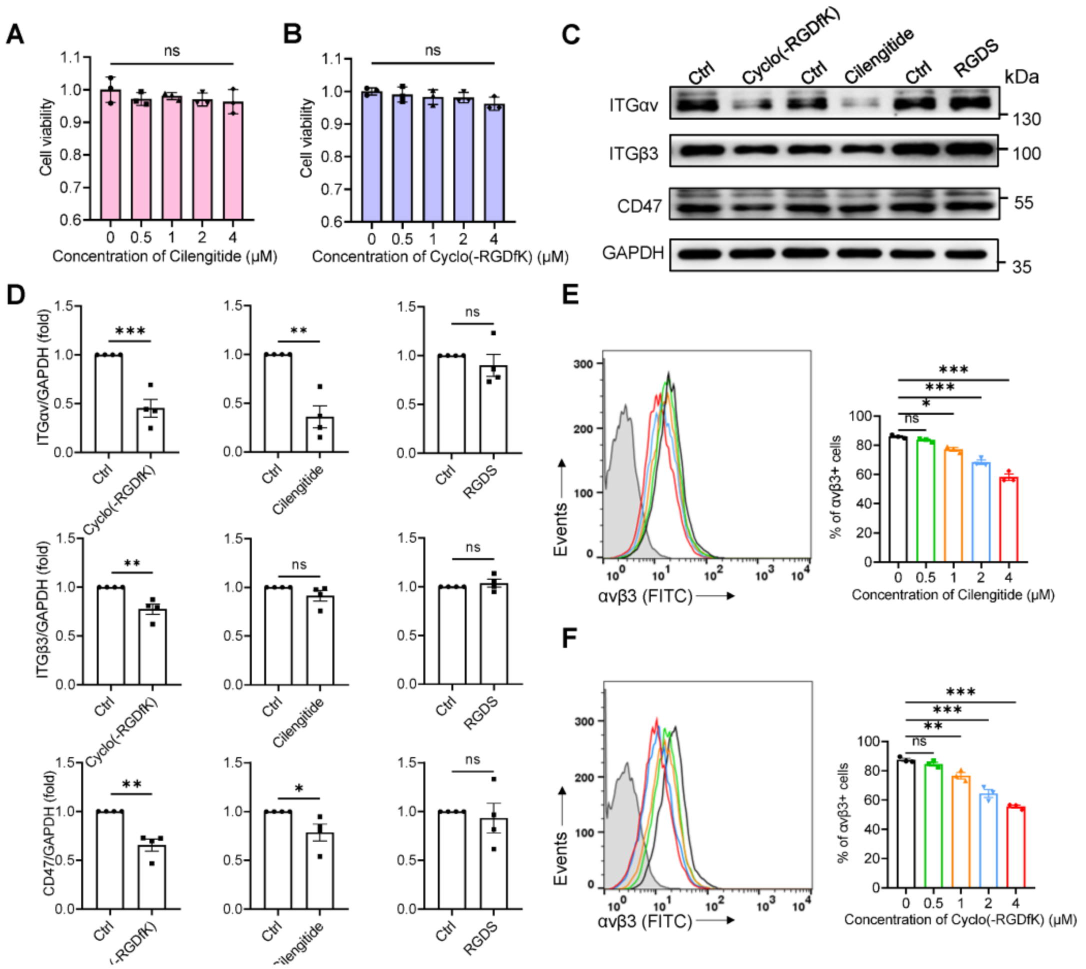

Supplementary Figure S10. Reduction of CD47 expression by integrin αvβ3-specific inhibitors. (A, B) MTT assay determining the viability of MDA-MB-231 cells after 48 hours of exposure to varying concentrations of cilengitide (A) and cyclo(-RGDfK) (B) (0, 0.5, 1, 2, 4 μM). (C, D) Western blot analysis (C) and pooled protein expression data of ITGαv, ITGβ3, and CD47 (**C**, **D**) Western blot analysis (C) and pooled protein expression data of ITGαv, ITGβ3, and CD47 (D). MDA-MB-231 cells were treated with cilengitide, cyclo(-RGDfK), and RGDS (4 μM) for 48 hours. (**E**, **F**) Flow cytometry analysis of αvβ3 surface expression in MDA-MB-231 cells treated with different concentrations of αvβ3 inhibitors, cilengitide, and cyclo(-RGDfK) (0, 0.5, 1, 2, 4 μM) for 48 hours, with pooled data. All data are presented as mean ± s.e.m, n = 3-4 independent experiments; $^{*}P < 0.05$, $^{**}P < 0.001$, $^{***}P < 0.001$ versus control, unpaired t test (D), one-way ANOVA with Dunnett's post-hoc test (A, B, E and F); (A),

(F (4, 10) = 0.7900, $p = 0.5575$), (B), (F (4, 10) = 1.621, $p = 0.2439$); (E), (F (4, 10) = 69.60, $P < 0.0001$),

(**F**), (F (4, 10) = 55.60, $P < 0.0001$); ns, not significan

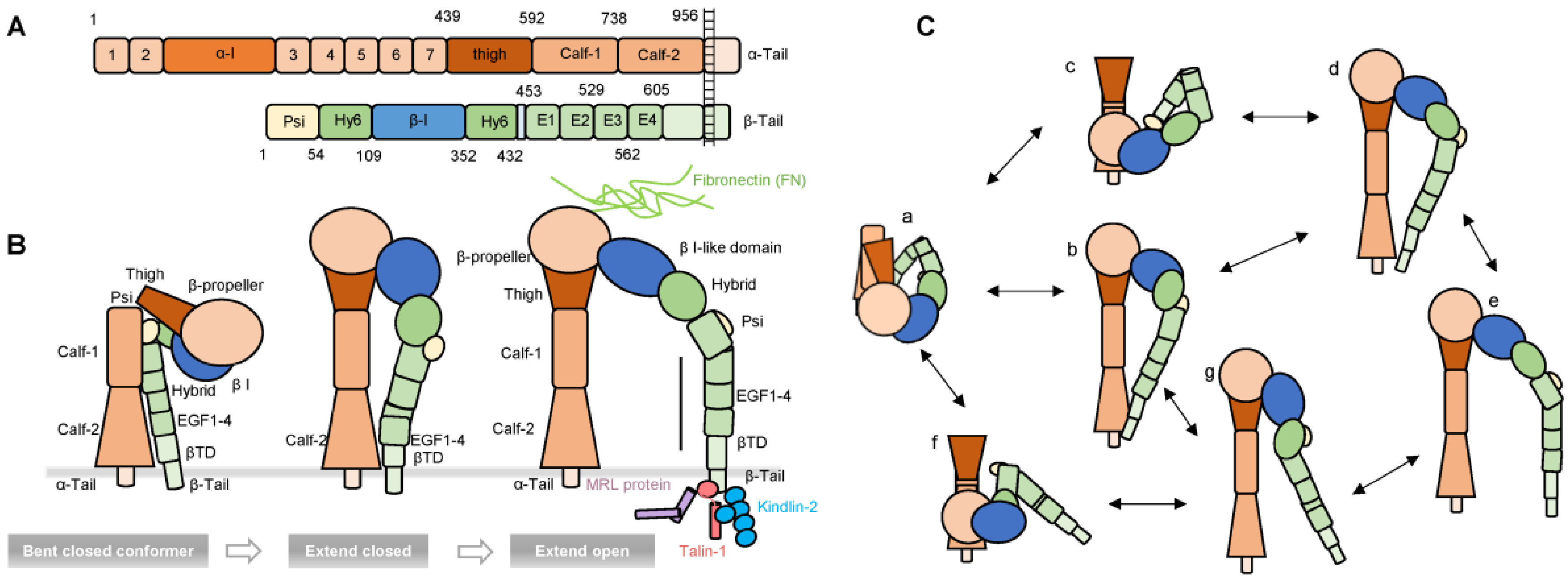

Supplementary Figure S11. Overview of integrin αvβ3 conformation and structural domain composition. (**A**) Domain organization of integrin α and β subunits. (**B**) Schematic representation of the three states/conformations of integrins. 1. Non-bound/inactive resting state. In this state, integrins are in a bent conformer with tightly linked transmembrane and cytoplasmic regions. 2. Extended closed conformation. This is an intermediate conformation that is active but has low affinity for external ligands. 3. Extended open conformation. Activation with high affinity is marked by separation of the cytoplasmic leg domains. (**C**) Intermediate states during structure determination indicating pathways likely stimulated by extracellular ligand binding and intracellular subunit segregation signals. The upper pathway is

probably stimulated by extracellular ligand binding; the lower pathway is stimulated by intracellular α and β subunit transmembrane domains by intracellular signals.

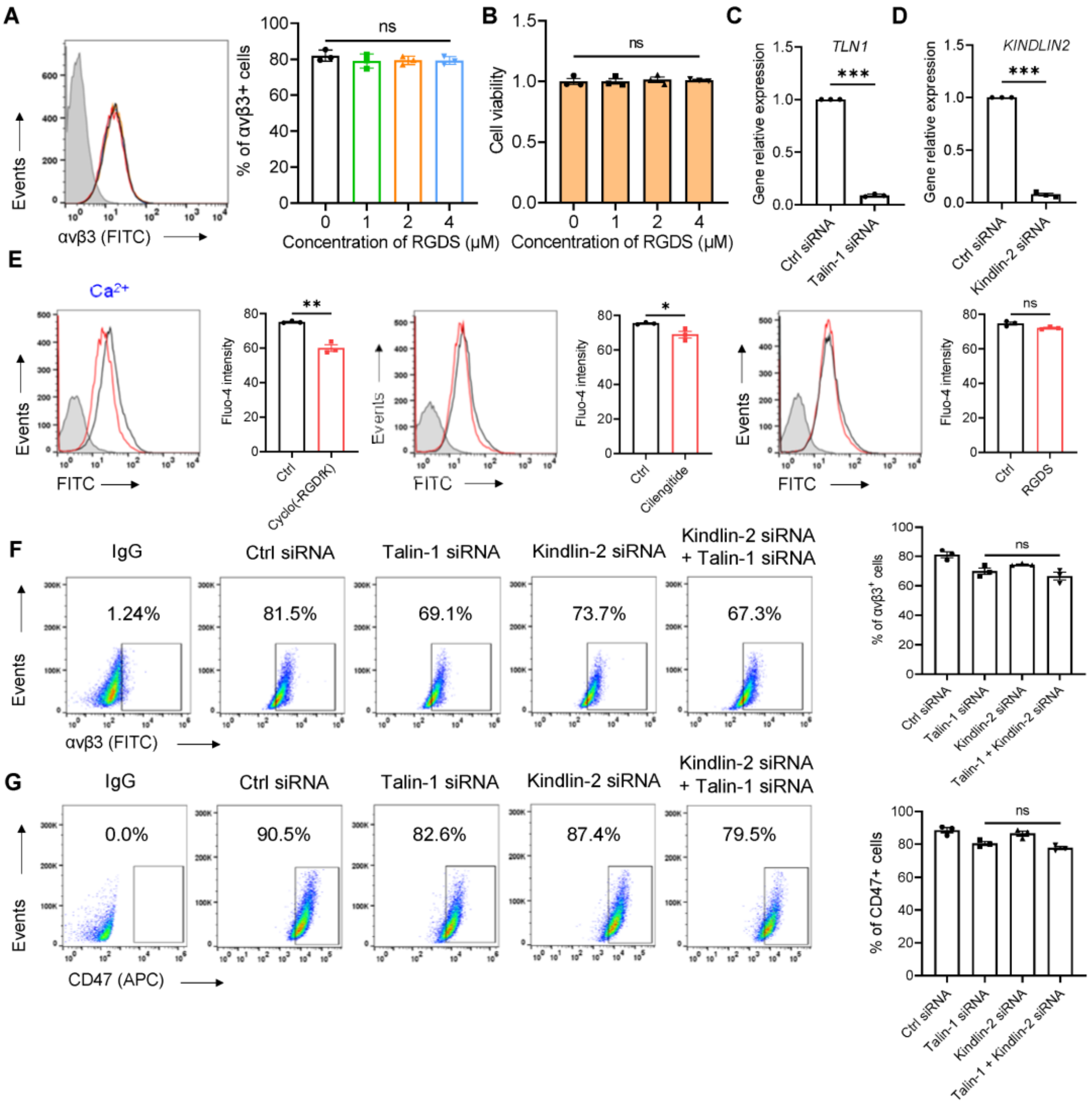

Supplementary Figure S12. Impact of blocking αvβ3 activation on surface expression of αvβ3 and CD47 on MDA-MB-231 Cells. (**A**) Flow cytometry analysis of αvβ3 surface expression on MDA-MB-231 cells treated with various concentrations of integrin αvβ3 inhibitor RGDS (0, 1, 2, 4 μM) for 48 hours, with pooled data. **(B)** Viability of MDA-MB-231 cells measured by MTT after treatment with different concentrations of RGDS (0, 1, 2, 4 μM) for 48 hours. (**C**) RT-qPCR analysis of TLN1 mRNA expression in MDA-MB-231 cells post Talin-1 siRNA-mediated knockdown. (**D**) RT-qPCR analysis of KINDLIN2 mRNA expression in MDA-MB-231 cells post Kindlin-2 siRNA-mediated knockdown. (**E**) Flow cytometry analysis of intracellular calcium level alterations in MDA-MB-231 cells treated with

cyclo(-RGDfK), cilengitide, and RGDS (4 μM) for 48 hours. (**F**, **G**) Flow cytometry analysis of αvβ3 and CD47 surface expression in MDA-MB-231 cells 48 hours after combined Talin-1 and Kindlin-2 siRNA-mediated knockdown, with pooled data of cell surface markers αvβ3 and CD47. All data are expressed as mean ± s.e.m., n = 3 independent experiments; $^{*}P < 0.05$, $^{**}P < 0.001$, $^{***}P < 0.001$ versus control, unpaired t test (C-D). one-way ANOVA with Dunnett's post-hoc test (A, B); (A), (F (3, 8) = 0.6635, p = 0.5974), (B), (F (4, 10) = 0.5846, p = 0.6811), (F), (F (3, 8) = 10.15, p = 0.0042), (G), (F (3, 8) = 14.41, p = 0.0014). ns, not significant.

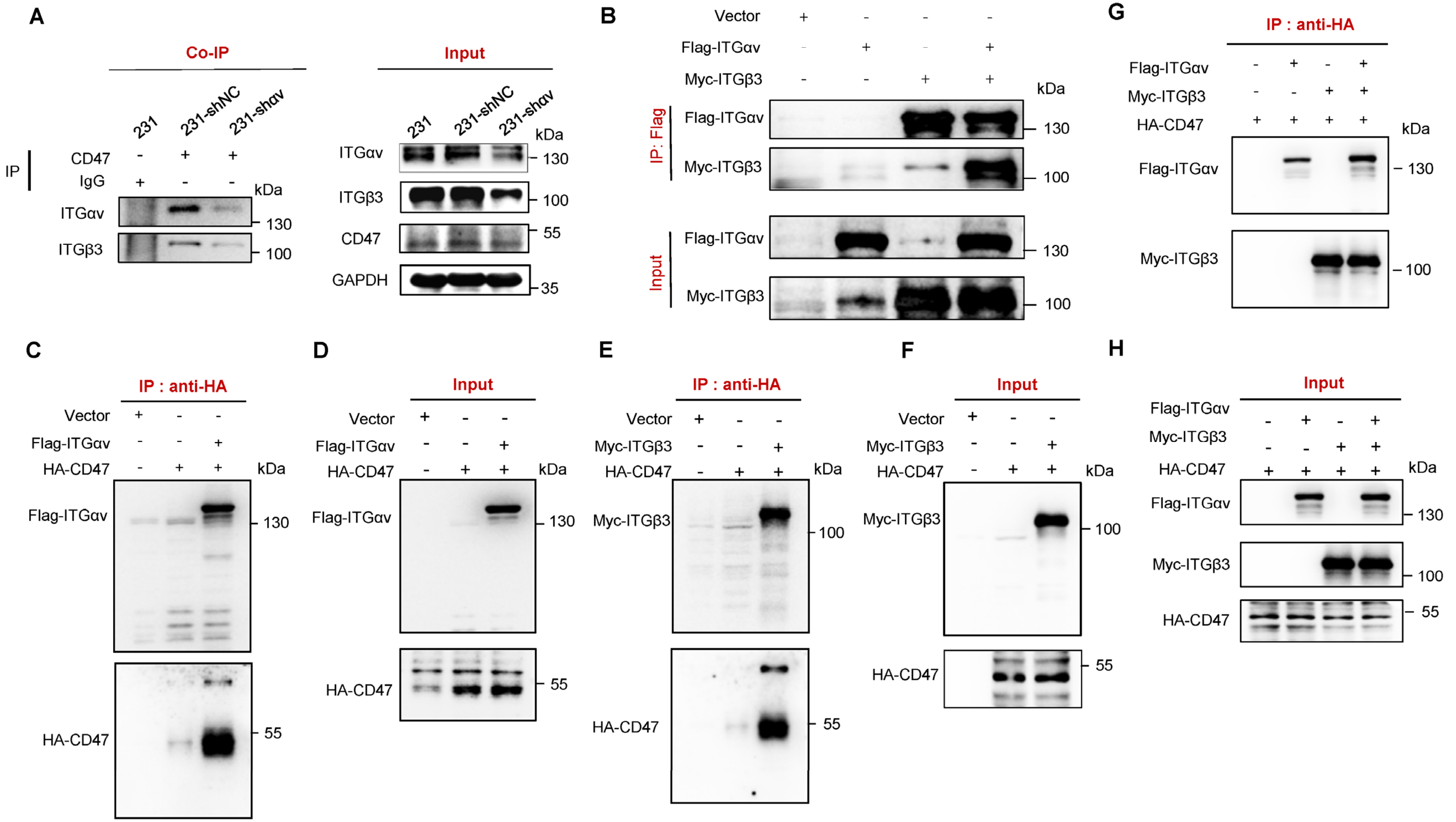

Supplementary Figure S13. Interactions between CD47 and integrin αvβ3. (**A**) Immunoprecipitation of MDA-MB-231 shNC and shαv cell lysates using control IgG antibody and CD47 antibody revealed a reduction in CD47 binding to αvβ3 upon shαv knockdown compared to control shNC. (**B**) Co-transfection of HEK293

cells with plasmids expressing Flag-ITGαv and Myc-ITGβ3 resulted in the precipitation of both Flag-ITGαv and Myc-ITGβ3 with Flag antibodies. (**C-H**) Co-transfection of HEK293 cells with plasmids expressing Flag-ITGαv or Myc-ITGβ3 and HA-CD47 led to the precipitation of both Flag-ITGαv and Myc-ITGβ3 with HA antibody in three independent experiments.

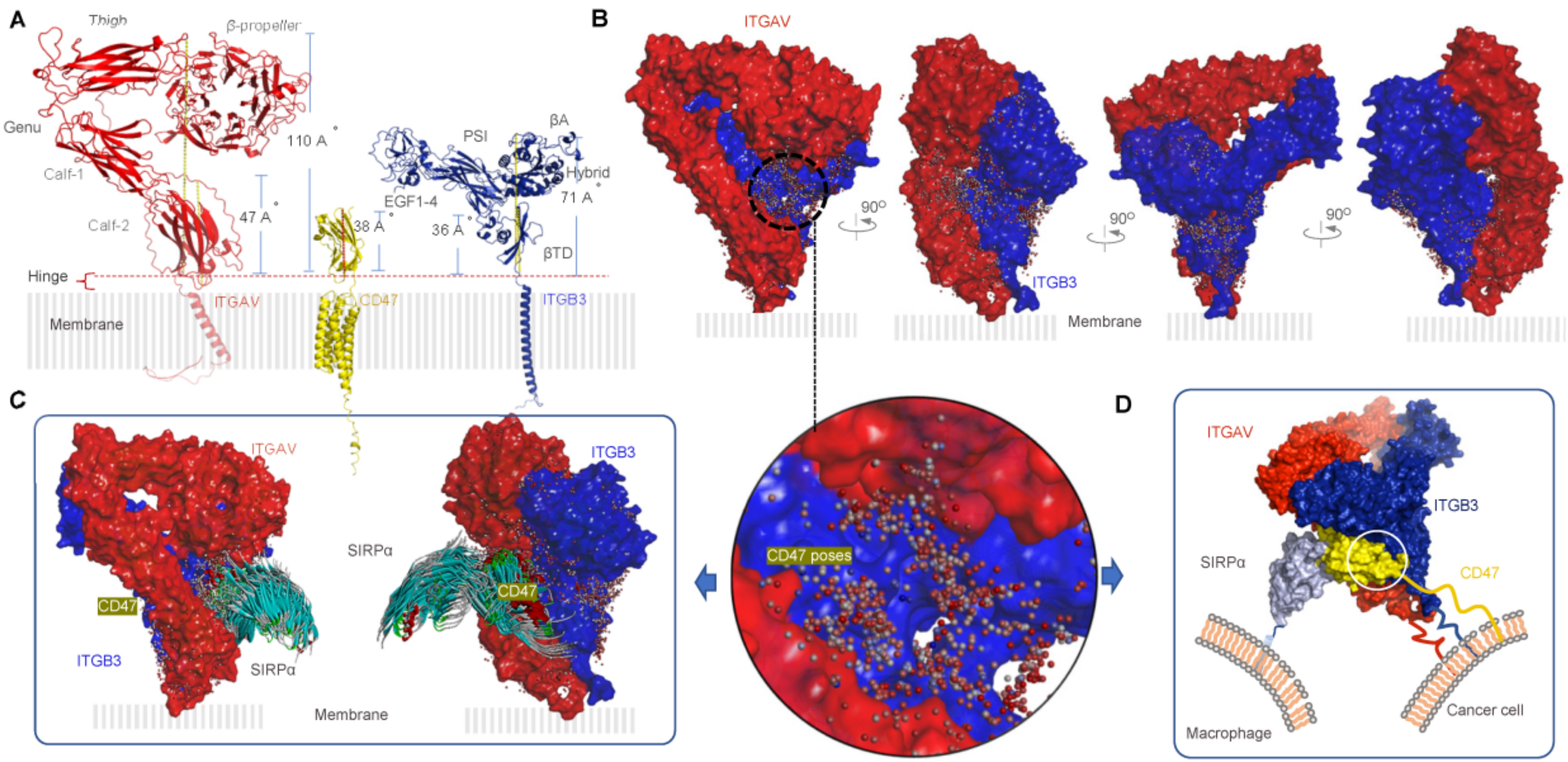

Supplementary Figure S14. Interaction model of CD47 and integrin αvβ3. (**A**) Three-dimensional architecture of CD47 and integrin αvβ3 with relative sizes of extracellular structural domains (ECDs). (**B-D**) Development of CD47 (yellow)/integrin αvβ3 (αv subunit in red and β3 subunit in blue) interaction models. Models were generated through clustering analysis of 2000 possible binding poses using *Z-dock* (B), followed by manual selection (C) to obtain a plausible interaction pattern

(D) of CD47/αvβ3-ECD between macrophage SIRPα and tumor cells.

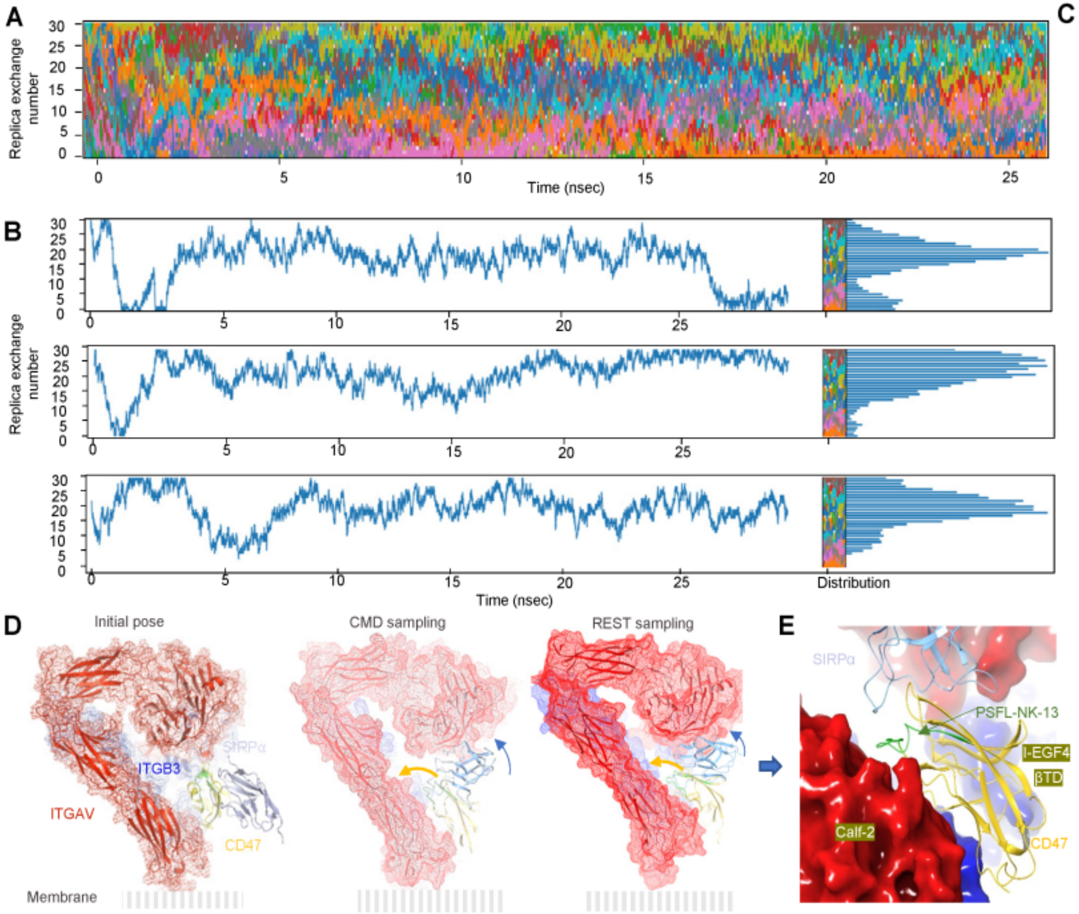

C

Replica Pairs (K)	Total Exchanges	No. Accepts	No. Rejects	Accept Ratio
T00-T01	13789	4443	9346	0.322
T01-T02	8131	2650	5481	0.326
T02-T03	9860	3139	6721	0.318
T03-T04	9365	3125	6240	0.334
T04-T05	9504	3183	6321	0.335
T05-T06	9462	3134	6328	0.331
T06-T07	9471	3108	6363	0.328
T07-T08	9463	3108	6355	0.328
T08-T09	9497	3177	6320	0.335
T09-T10	9465	3129	6336	0.331
T10-T11	9570	3225	6345	0.337
T11-T12	9373	3272	6101	0.349
T12-T13	9501	3183	6318	0.335
T13-T14	9544	3359	6185	0.352
T14-T15	9324	3230	6094	0.346
T15-T16	9658	3317	6341	0.343
T16-T17	9383	3261	6122	0.348
T17-T18	9453	3217	6236	0.340
T18-T19	9492	3466	6026	0.365
T19-T20	9354	3247	6107	0.347
T20-T21	9531	3411	6120	0.358
T21-T22	9494	3241	6253	0.341
T22-T23	9385	3384	6001	0.361
T23-T24	9542	3423	6119	0.359
T24-T25	9505	3403	6102	0.358
T25-T26	9431	3267	6164	0.346
T26-T27	9444	3238	6206	0.343
T27-T28	9499	3318	6181	0.349
T28-T29	9672	3439	6233	0.356
T29-T30	8288	2962	5326	0.357
T30-T31	13632	5105	8527	0.374

Supplementary Figure S15. Optimization of the CD47/αvβ3-ECD interaction model. (**A-C**) Optimization of the CD47/αvβ3-ECD interaction model using Replica Exchange with Solute Tempering (REST) enhanced conformational sampling (temperature span 300-372K, 32 replicates (A), with exchange probabilities above 0.3

(B), and sufficient exchange between individual replicates, exemplified by replicates 3, 16, and 30 (C)). (**D**) After optimization by 400-ns CMD or REST, CD47 is further approached to the cavity formed by αv of Calf-2 and β3 of IEGF-4 and βTD. (**E**) In the optimized model, CD47-ECD is inserted obliquely at the interface of the structural domains of the αv subunit Calf-2 and the I-EGF4 and βTD of the β3 subunit. αvβ3 is represented by red and blue van der Waals surfaces, while CD47 and SIRPα are represented by yellow and sky-blue cartoons, respectively. PSFL-NK13$^{M46\text{-}K57}$ (green cartoon) is located at the interface formed by the αv subunit of Calf-2 and the β3 subunit of I-EGF4 and βTD.

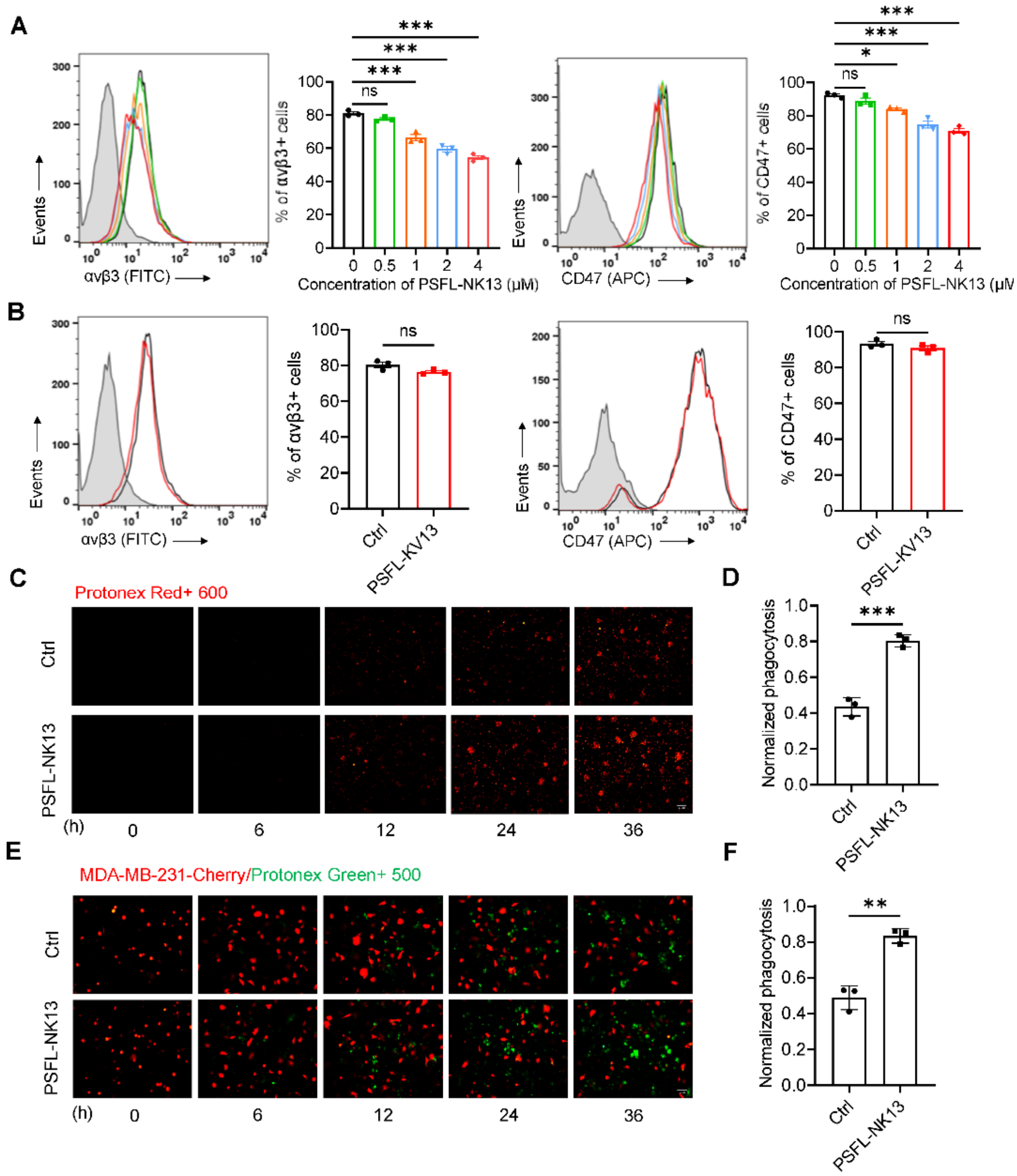

Supplementary Figure S16. PSFL-NK13 modulates CD47 and αvβ3 expression and enhances tumor phagocytosis by macrophages. (**A**) Flow cytometry analysis of αvβ3 and CD47 surface expression on MDA-MB-231 cells treated with varying concentrations of PSFL-NK13 peptide (0, 0.5, 1, 2, and 4 μM) for 48 hours, with data summary. (**B**) Flow cytometry analysis of αvβ3 and CD47 surface expression on MDA-MB-231 cells treated with PSFL-KV13 peptide (4 μM) for 48 hours, with pooled data. (**C**, **D**) Representative fluorescence microscopy images depicting in vitro phagocytosis of MDA-MB-231 cells by macrophages (Protonex Red+, red) after 36 hours of co-culture with control or PSFL-

NK13 (4 μM). (**E**, **F**) Representative fluorescence microscopy images illustrating in vitro phagocytosis of MDA-MB-231 cells (Cherry+, red) by macrophages (Protonex green+, green) after 36 hours of co-culture with control or PSFL-NK13 peptide (4 μM). Scale bar = 50 μm. (**G**) Flow cytometry analysis and pooled data of αvβ3 (LM609) surface expression on MDA-MB-231 cells treated with PSFL-NK13 (4 μM) for 48 hours. (**H**) Flow cytometry analysis of altered intracellular calcium levels in MDA-MB-231 cells treated with PSFL-NK13 peptide (4 μM) for 48 hours, with pooled data. All data are expressed as mean ± s.e.m., n = 3 independent experiments; $^{*}P < 0.05$, $^{**}P < 0.001$, $^{***}P < 0.001$ versus control, unpaired t test (B, D, F, G and H), one-way ANOVA with Dunnett's post-hoc test (A); (A), (F (4, 10) =70.37, $P < 0.0001$), (F (4, 10) =35.53, $P < 0.0001$). ns, not significant.

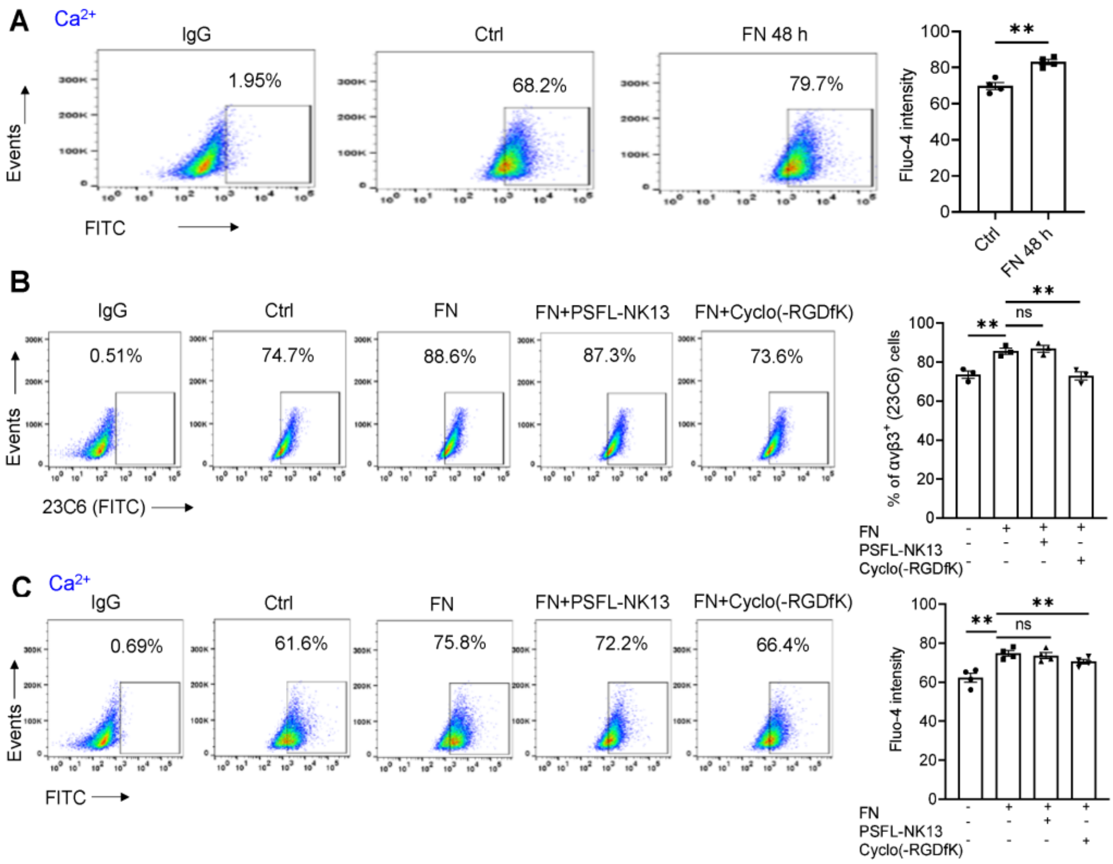

Supplementary Figure S17. PSFL-NK13 does not affect fibronectin-mediated αvβ3 activation (**A**) Flow cytometry analysis of intracellular calcium level alterations in MDA-MB-231 cells treated with FN (20 μg/ml) for 48 hours. (**B**) Flow cytometry analysis of αvβ3 (using 23C6) surface expression in MDA-MB-231 cells treated with FN (20 μg/ml), either alone or in combination with PSFL-NK13 (4 μM), or with cyclo(-RGDfK) (4 μM) for 48 hours. (**C**) Flow cytometry analysis of intracellular calcium level changes in MDA-MB-231 cells treated with FN (20 μg/ml), alone or in combination with PSFL-NK13 (4 μM), or with cyclo(-RGDfK) (4 μM) for 48 hours. All data are expressed as mean ± s.e.m., n = 3 independent experiments; $^{**}P < 0.001$ versus control, unpaired t test (A, B). one-way ANOVA with Dunnett's post-hoc test (C); (C), (F (3, 12) =11.51, $p = 0.0008$). ns, not significant.

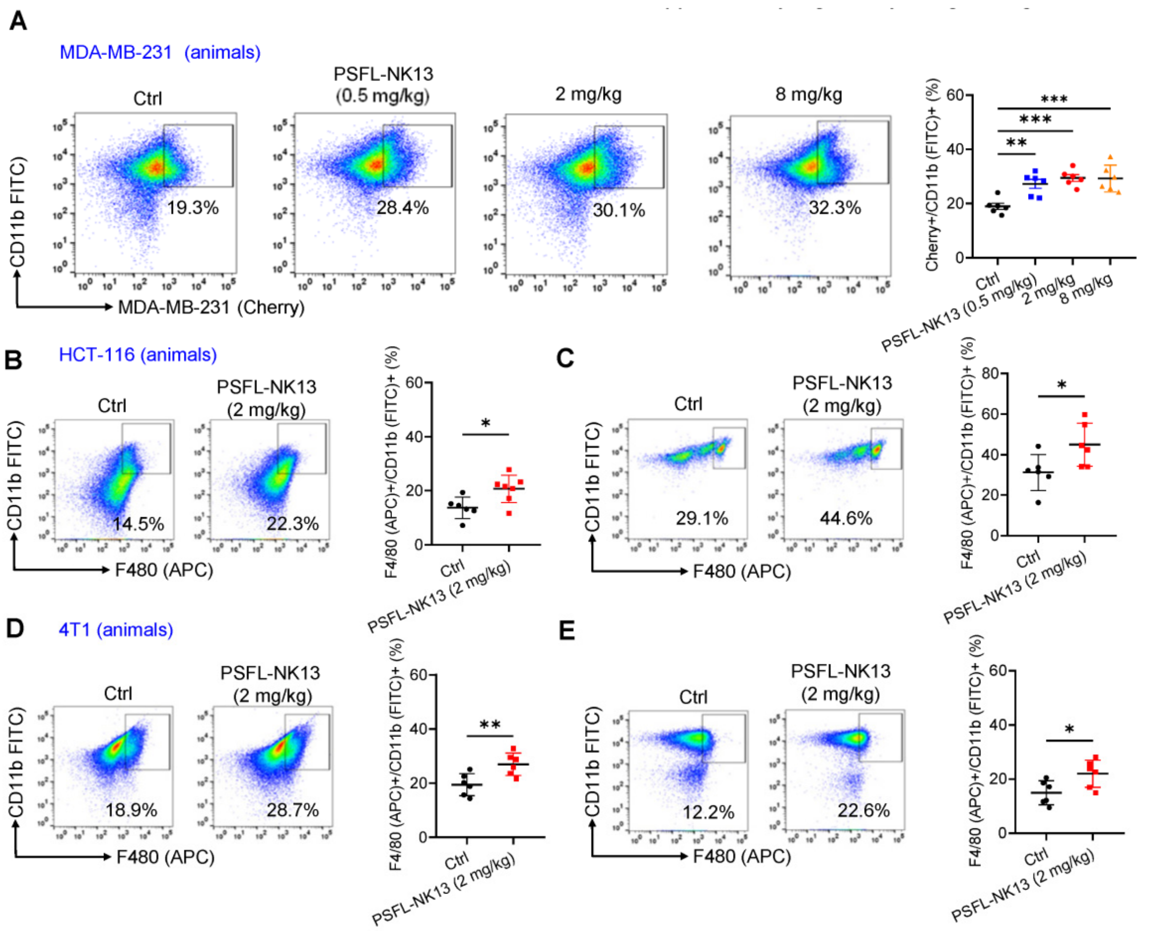

Supplementary Figure S18. PSFL-NK13 enhances macrophage infiltration *in vivo*. (**A**) Representative flow cytometry plots illustrating macrophage phagocytosis in Cherry+ MDA-MB-231 shNC tumors (6 mice per group), with quantification of macrophage infiltration assessed as MDA-MB-231(Cherry)+/CD11b(FITC)+. (**B**, **C**) Representative flow cytometry analysis of F4/80+/CD11b+ macrophages in HCT116 tumors (B) and PBMCs (C), with quantification of macrophage ratio assessed as F4/80(APC)+/CD11b(FITC)+ by flow cytometry. (**D**, **E**) Representative flow cytometry analysis of F4/80+/CD11b+ macrophages in 4T1 tumors (D) and PBMCs (E), with quantification of macrophage proportion assessed as F4/80(APC)+/CD11b(FITC)+ by flow cytometry. All data are expressed as mean ± s.e.m., n = 6 independent experiments; $^{*}P < 0.05$, $^{**}P < 0.001$, $^{***}P < 0.001$ versus control, unpaired t-test (B-E). one-way ANOVA with Dunnett's post-hoc test (A); (A), (F (3, 20) =10.19, $p = 0.0003$).

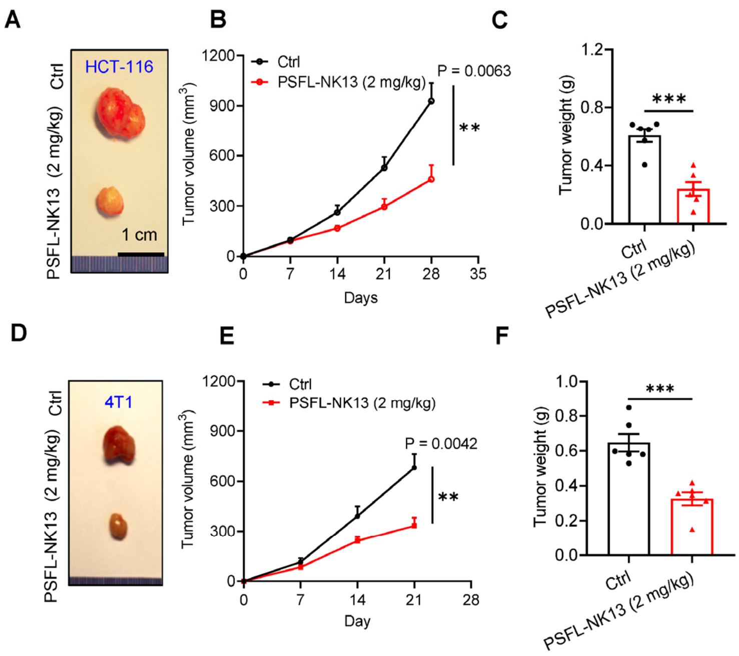

Supplementary Figure S19. Suppression of HCT-116 and 4T1 developments using PSFL-NK13 (**A-C**) Female nude mice were subcutaneously implanted with right dorsal allogeneic HCT116 cells (1 × 10^6), and tumor growth rates in mice (6 mice per group) treated with or without PSFL-NK13 were compared. Image represents 6 independent experiments (A). Scale bar = 1 cm. Tumor growth kinetics of nude mice were compared for 28 days (6 mice per group). Tumor volume (B) and weight (C) were measured after three weeks of PSFL-NK13 treatment (2 mg/kg). (**D-F**) 4T1 breast cancer cells (1 × 10^6) were implanted into the mammary fat pad of BALB/c mice, and tumor growth rates in mice (6 mice per group) treated with or without PSFL-NK13 were compared. Image represents 6 independent experiments (D). Scale bar = 1 cm. After two weeks of PSFL-NK13 treatment (2 mg/kg), tumor volume (E) and weight (F) were measured. All data are expressed as mean ± s.e.m; $^{*}P < 0.05$, $^{**}P < 0.001$, $^{***}P < 0.001$ versus control, unpaired t test (B, C, E and F).

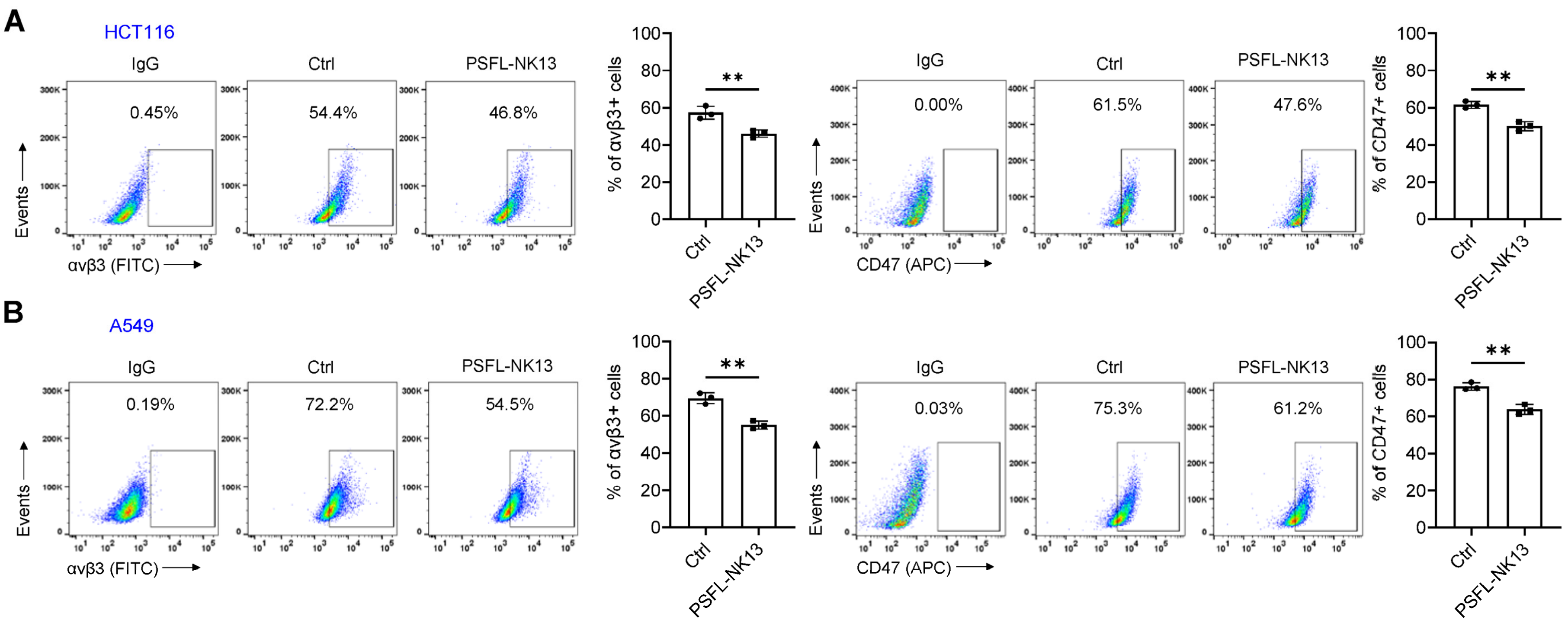

Supplementary Figure S20. PSFL-NK13 reduces CD47 and αvβ3 surface expression in various tumor cells. (**A-B**) Flow cytometry analysis of αvβ3 and CD47 surface expression on HCT116 (A) and A549 (B) treated with PSFL-NK13 peptide (4 μM) for 48 hours, along with pooled data. All data are expressed as mean ± s.e.m., n = 3 independent experiments; $^{**}P < 0.001$, $^{***}P < 0.001$ versus control, unpaired t test.

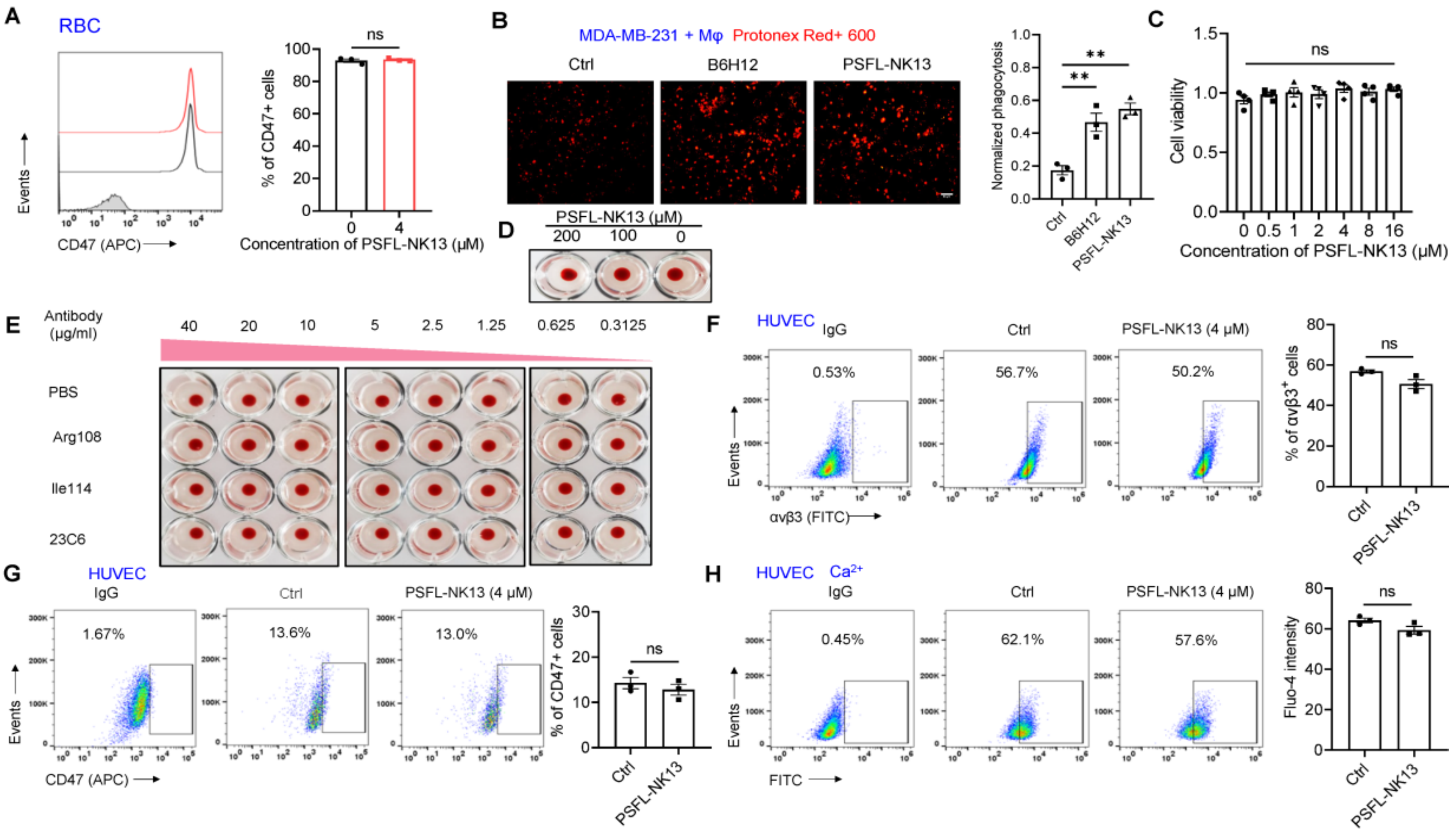

Supplementary Figure S21. PSFL-NK13 does not induce erythrocyte aggregation or phagocytosis by macrophages. (**A**) Flow cytometry analysis of CD47 surface expression on RBCs treated with PSFL-NK13 peptide (4 μM) for 48 hours, along with summary data. (**B**) Representative images of live cell microscopy phagocytosis

assays depicting pHrodo-red+ MCF-7 cells treated with CD47 antibody (B6H12, 10 μg/ml) or PSFL-NK13 (4 μM) for 36 hours, accompanied by quantitative analysis of mean fluorescence intensity signal in confocal images. Scale bar = 50 μm. (**C**) Viability of MDA-MB-231 cells measured by MTT assay at various concentrations of PSFL-NK13 peptide (0, 0.5, 1, 2, 4, 8, and 16 μM) for 48 hours. (**D**) Erythrocyte suspensions inoculated in 96-well circular (U) bottom plates and incubated with PSFL-NK13 peptide (100 and 200 μM). (**E**) Erythrocyte suspensions inoculated in 96-well circular (U) bottom plates and incubated with αv antibody (Arg108), β3 antibody (Ile114), or αvβ3 (23C6). Antibodies were diluted in two gradients from 40 μg/ml to 0.3125 μg/ml. (**F**, **G**) Flow cytometry analysis of αvβ3 (F) and CD47 (G) surface expression on HUVEC cells treated with PSFL-NK13 peptide (4 μM) for 48 hours, along with pooled data. (**H**) Flow cytometry analysis of intracellular calcium level changes in HUVEC cells treated with PSFL-NK13 peptide (4 μM) for 48 hours. All data are expressed as mean ± s.e.m., n = 3-4 independent experiments; $^{**}P < 0.001$ versus control, unpaired t test (A, F, G, H); one-way ANOVA with Dunnett's post-hoc test (B, C), (B), ($F(2, 6) = 22.46$, $p = 0.0016$), (C), ($F(6, 21) = 1.113$, $p = 0.3883$). ns, not significant.

Supplementary Tables 1-6

Table S1. The surface expression of αvβ3 and CD47 in tumor cell lines knocked down integrin αv or β3

Cells		Ctrl siRNA	αv siRNA-1	αv siRNA-2	Influenced percentage (%) of αv siRNA-1	Influenced percentage (%) of αv siRNA-2	Ctrl siRNA	β3 siRNA-1	β3 siRNA-2	Influenced percentage (%) of β3 siRNA-1	Influenced percentage (%) of β3 siRNA-2
MDA-MB-231	αvβ3	85.7 ± 0.3	49.1 ± 1.7	50.0 ± 3.5	42.7 ± 1.8	41.7 ± 3.9	87.3 ± 2.0	76.3 ± 2.3	76.8 ± 0.8	12.5 ± 0.9	11.9 ± 1.2
	CD47	96.2 ± 1.0	72.1 ± 1.4	73.3 ± 2.0	25.0 ± 0.7	23.8 ± 1.3	94.3 ± 1.1	87.6 ± 1.5	86.2 ± 1.8	7.1 ± 2.5	8.5 ± 2.8
MCF 7	αvβ3	69.8 ± 1.9	42.9 ± 1.4	43.3 ± 3.1	38.5 ± 1.7	38.1 ± 3.3	69.3 ± 1.4	62.6 ± 0.8	60.9 ± 2.4	9.6 ± 1.7	12.2 ± 0.9
	CD47	92.3 ± 0.7	64.7 ± 5.5	68.0 ± 4.9	30.0 ± 2.4	26.4 ± 4.8	92.2 ± 1.7	89.0 ± 2.3	86.0 ± 3.2	3.6 ± 1.1	6.7 ± 2.1
HCT 116	αvβ3	38.1 ± 6.9	24.5 ± 4.6		35.6 ± 4.4						
	CD47	58.7 ± 4.9	47.1 ± 3.9		19.7 ± 2.3						
A549	αvβ3	59.9 ± 4.0	43.7 ± 7.4		27.4 ± 8.0						
	CD47	69.3 ± 7.3	54.4 ± 1.8		21.0 ± 6.3						
U87	αvβ3	62.5 ± 3.4	33.9 ± 6.1		45.8 ± 7.8						
	CD47	94.6 ± 0.9	84.0 ± 3.4		11.2 ± 2.8						

Table S2. The surface expression of αvβ3 and CD47 in MDA-MB-231 cells

	αvβ3 expression (%)	Influenced percentage (%)	/αv (%)	CD47 expression (%)	Influenced percentage (%)	/αv (%)
Ctrl siRNA	81.4 ± 3.7			93.8 ± 1.8		
Talin-1 siRNA	70.9 ± 1.5 *	12.9 ± 2.2	34.3	85.6 ± 1.2 *	8.8 ± 0.5	37.9
Ctrl	81.9 ± 0.3			91.6 ± 1.6		
EGTA	69.7 ± 2.2 **	14.8 ± 2.4	39.4	81.2 ± 1.9 *	11.3 ± 0.6	48.7
Ctrl	64.8 ± 3.4			83.4 ± 1.5		
FN	77.2 ± 3.2 ***	19.3 ± 2.7	51.3	90.5 ± 1.7 **	8.5 ± 0.5	36.6
Ctrl siRNA	81.5 ± 3.2			91.0 ± 1.4		
αv siRNA	50.9 ± 3.2 ***	37.6 ± 2.5		69.9 ± 3.7 **	23.2 ± 2.9	

Table S3. Primer sequences for the RT-qPCR

Names		*Sequence (5' to 3')*
ITGAV	Forward	5’- ATCTGTGAGGTCGAAACAGGA -3’
	Reverse	5’- TGGAGCATACTCAACAGTCTTTG -3’
ITGB3	Forward	5’- GTGACCTGAAGGAGAATCTGC -3’
	Reverse	5’- CCGGAGTGCAATCCTCTGG -3’
CD47	Forward	5’- AGAAGGTGAAACGATCATCGAGC -3’
	Reverse	5’- CTCATCCATACCACCGGATCT -3’
SIRPA	Forward	5’- GGCCTCAACCGTTACAGAGAA -3’
	Reverse	5’- GTTCCGTTCATTAGATCCAGTGT -3’
ITGA2B	Forward	5’-GATGAGACCCGAAATGTAGGC-3’
	Reverse	5’- GTCTTTTCTAGGACGTTCCAGTG -3’
ITGA5	Forward	5’- GGCTTCAACTTAGACGCGGAG -3’
	Reverse	5’- TGGCTGGTATTAGCCTTGGGT -3’
ITGB1	Forward	5’- CCTACTTCTGCACGATGTGATG -3’
	Reverse	5’- CCTTTGCTACGGTTGGTTACATT -3’
ITGB6	Forward	5’- TCCATCTGGAGTTGGCGAAAG -3’
	Reverse	5’- TCTGTCTGCCTACACTGAGAG -3’
ITGB8	Forward	5’- ACCAGGAGAAGTGTCTATCCAG -3’
	Reverse	5’- CCAAGACGAAAGTCACGGGA -3’
TLN1	Forward	5’- GACGATGCAGTTTGAGCCG -3’
	Reverse	5’- GGGTCATCATCTGACAGAAAGAG -3’
KINDLIN2	Forward	5’- TCTGACCATGCTCTCTGGTG -3’
	Reverse	5’- AGTTCTTCGGGGTGTCTGATA -3’
GAPDH	Forward	5’- TTGGTATCGTGGAAGGACT -3’
	Reverse	5’- GGATGATGTTCTGGAGAGC -3’

Table S4. Antibodies used in this study

Name	Company	Catalog number	Assay
CD47 Antibody	Santa Cruz	sc-12730	WB, Phagocytosis
integrin αv Antibody	Cell Signaling Technology	4711S	WB, Phagocytosis
integrin β3 Antibody	Cell Signaling Technology	13166S	WB, Phagocytosis
GAPDH Monoclonal Antibody	Proteintech	60004-1-Ig	WB
anti-rabbit IgG HRP-conjugated	Cell Signaling Technology	7074S	WB
anti-mouse IgG HRP-conjugated	Cell Signaling Technology	7076S	WB
Purified anti-human CD47 Antibody	Biolegend	323102	IP
Purified anti-human αvβ3 Antibody	Biolegend	304402	IP, Phagocytosis
Anti-Flag magnetic beads	Bimake	B26101	IP
Anti-HA magnetic beads	Bimake	B26101	IP
Mouse mAb IgG XP® Isotype Control	Cell Signaling Technology	5873	IP
Pierce™ Anti-c-Myc Magnetic Beads	Thermo Fisher	88842	IP
Pierce™ Anti-c-HA Magnetic Beads	Thermo Fisher	88836	IP
HA-Tag (C29F4) Rabbit mAb	Cell Signaling Technology	3724S	IP
DYKDDDDK Tag Antibody (Binds to same epitope as Sigma's Anti-FLAG® M2 Antibody)	Cell Signaling Technology	2368	IP
HA Tag Monoclonal antibody	Proteintech	66006-2-Ig	IP
MYC tag Polyclonal antibody	Proteintech	16286-1-AP	IP
DYKDDDDK tag Polyclonal antibody (Binds to FLAG® tag epitope)	Proteintech	20543-1-AP	IP
FITC anti-human αvβ3 Antibody	Biolegend	304402	FACS
FITC anti-human αv Antibody	Biolegend	327908	FACS
CD47 Monoclonal Antibody	Invitrogen	17-0479-42	FACS
PE anti-human CD172α (SIRPα) Antibody	Biolegend	372103	FACS
Anti-Integrin alpha V beta 3 Antibody	Abcam	ab190147	FACS, Phagocytosis
Goat anti-Mouse IgG FITC	Absin	abs20012	FACS
PerCP/Cyanine5.5 anti-mouse CD45	Biolegend	103132	FACS
APC anti-mouse F4/80	Biolegend	123116	FACS
FITC anti-mouse/human CD11b	Biolegend	101206	FACS

Table S5. Primer sequences for the plasmid construction

Names	Sequence (5′ to 3′)
AV-F	5’-CAAGCTGGCTAGCGTTATGGCTTTTCCGCCGCGG-3’
AV-R	5’-TCACTTGTCATCGTCATCCTTGTAGTCAGTTTCTGAGTTTCCTTCACCATTTTCAT-3’
3.1-AV-F	5’-CAAGGATGACGATGACAAGTGAGAATTCTGCAGATATCCAGCACAGTG-3’
3.1-AV-R	5’-CGGAAAAGCCATAACGCTAGCCAGCTTGGGTCTC-3’
B3-F	5’-GAGACCCAAGCTGGCTAGCTGTGGATATCTGCAGAATTCGC-3’
B3-R	5’-TCCTCTTCAGAGATGAGTTTCTGCTCAGTGCCCCGGTACGTGATATTGGT-3’
3.1-B3-F	5’-AGAAACTCATCTCTGAAGAGGATCTGTGAGAATTCTGCAGATATCCAGCACAGTG-3’
3.1-B3-R	5’-GCGAATTCTGCAGATATCCAACGCTAGCCAGCTTGGGTCTC-3’
CD47-F	5’-CAAGCTGGCTAGCGTTGCCACCATGTGGCCCCTGGTAG-3’
CD47-R	5’-TCATGCATAATCCGGAACATCATACGGATAGTTATTCCTAGGAGGTTG-3’
3.1-CD47-F	5’-CGTATGATGTTCCGGATTATGCATGAGAATTCTGCAGATATCCAGCACAGTG-3’
3.1-CD47-R	5’-CACATGGTGGCAACGCTAGCCAGCTTGGGTCT-3’
CD47Δ26-32-F	5’-GCTCAGCTACTATTTAATAAAACATGTAATGATACTGTCGTCATTCC-3’
CD47Δ26-32-R	5’-GGAATGACGACAGTATCATTACATGTTTTATTAAATAGTAGCTGAGC-3’
CD47Δ44-50-F	5’-CTGTCGTCATTCCATGCTTTGTTACTACTGAAGTATACGTA-3’
CD47Δ44-50-R	5’-TACGTATACTTCAGTAGTAACAAAGCATGGAATGACGACAG-3’
CD47Δ51-55-F	5’-GTTACTAATATGGAGGCACAAAACGTAAAGTGGAAATTTAAAGGAAG-3’
CD47Δ51-55-R	5’-CTTCCTTTAAATTTCCACTTTACGTTTTGTGCCTCCATATTAGTAAC-3’
CD47Δ62-67-F	5’-CTGAAGTATACGTAAAGTGGAAATTTAAATTTGATGGAGCTCTAAACAAGTC-3’
CD47Δ62-67-R	5’-GACTTGTTTAGAGCTCCATCAAATTTAAATTTCCACTTTACGTATACTTCAG-3’
CD47Δ69-73-F	5’-GATATTTACACCTTTAAGTCCACTGTCCCC-3’
CD47Δ69-73-R	5’-GGGGACAGTGGACTTAAAGGTGTAAATATC-3’
CD47Δ80-85-F	5’-CTAAACAAGTCCACTGTCCCCACTATTGAAGTCTCACAATTACTAAAAG-3’
CD47Δ80-85-R	5’-CTTTTAGTAATTGTGAGACTTCAATAGTGGGGACAGTGGACTTGTTTAG-3’

Table S6. Nucleotide sequences of siRNA

Names	*Species*		*Sequence (5' to 3')*
av siRNA-1	*Homo sapiens*	Forward	5'-GCAGGUCUCAGUGUCUCUATT-3'
		Reverse	5'-UAGAGACACUGAGACCUGCTT-3'
av siRNA-2	*Homo sapiens*	Forward	5'-CCCUCUGACAUUGAUUGUUTT-3'
		Reverse	5'-AACAAUCAAUGUCAGAGGGTT-3'
β3 siRNA-1	*Homo sapiens*	Forward	5'-CCAACAACCCACUGUAUAATT-3'
		Reverse	5'-UUAUACAGUGGGUUGUUGGTT-3'
β3 siRNA-2	*Homo sapiens*	Forward	5'-GUGUCACCAUACAUGUAUATT-3'
		Reverse	5'-UAUACAUGUAUGGUGACACTT-3'
CD47 siRNA-1	*Homo sapiens*	Forward	5'-GGAUCAGCUCAGCUACUAUTT-3'
		Reverse	5'-AUAGUAGCUGAGCUGAUCCTT-3'
CD47 siRNA-2	*Homo sapiens*	Forward	5'-CCUCUUUGAAGAUGGAUAATT-3'
		Reverse	5'-UUAUCCAUCUUCAAAGAGGTT-3'
β1 siRNA	*Homo sapiens*	Forward	5'-GAGGCUCCAAAGAUAUAAATT-3'
		Reverse	5'-UUUAUAUCUUUGGAGCCUCTT-3'
β6 siRNA	*Homo sapiens*	Forward	5'-GUGGAUUUGAUGCAAUUAUTT-3'
		Reverse	5'-AUAAUUGCAUCAAAUCCACTT-3'
a5 siRNA	*Homo sapiens*	Forward	5'-CACCCGAAUUCUGGAGUAUTT-3'
		Reverse	5'-AUACUCCAGAAUUCGGGUGTT-3'
Talin-1 siRNA	*Homo sapiens*	Forward	5'-GAGAUGAGGAGUCUACUAUTT-3'
		Reverse	5'-AUAGUAGACUCCUCAUCUCTT-3'
Kindlin-2 siRNA	*Homo sapiens*	Forward	5'-GCCCAGGACUGUAUAGUAATT-3'
		Reverse	5'-UUACUAUACAGUCCUGGGCTT-3'
Ctrl siRNA	*Homo sapiens*	Forward	5'-UUCUCCGAACGUGUCACGUTT-3'
		Reverse	5'-ACGUGACACGUUCGGAGAATT-3'
